# Benzodiazepine-Free Cardiac Anesthesia for Reduction of Postoperative Delirium: A Cluster Randomized Crossover Trial

**DOI:** 10.1001/jamasurg.2024.6602

**Published:** 2025-01-29

**Authors:** Jessica Spence, P. J. Devereaux, Shun-Fu Lee, Frédérick D’Aragon, Michael S. Avidan, Richard P. Whitlock, C. David Mazer, Nicolas Rousseau-Saine, Raja Ramaswamy Rajamohan, Kane O. Pryor, Rael Klein, Edmund Tan, Matthew J. Cameron, Emily Di Sante, Erin DeBorba, Mary E. Mustard, Etienne J. Couture, Raffael Pereira Cezar Zamper, Michael W. Y. Law, George Djaiani, Tarit Saha, Stephen Choi, Peter Hedlin, D. Ryan Pikaluk, Wing Lam, Alain Deschamps, Chinthanie F. Ramasundarahettige, Jessica Vincent, William F. McIntyre, Simon J. W. Oczkowski, Braden J. Dulong, Christopher Beaver, Shelley A. Kloppenburg, Andre Lamy, Eric Jacobsohn, Emilie P. Belley-Côté

**Affiliations:** 1 Population Health Research Institute, Hamilton, Ontario, Canada; 2 Department of Anesthesia and Critical Care, McMaster University, Hamilton, Ontario, Canada; 3 Department of Health Research Methods, Evidence, and Impact, McMaster University, Hamilton, Ontario, Canada; 4 World Health Research Trust, Hamilton, Ontario, Canada; 5 Department of Medicine, Cardiology, McMaster University, Hamilton, Ontario, Canada; 6 Departement d’anesthésiologie, Université de Sherbrooke, Sherbrooke, Québec, Canada; 7 Department of Anesthesia, Washington University at St Louis School of Medicine, St Louis, Missouri; 8 Department of Surgery, Cardiac Surgery, McMaster University, Hamilton, Ontario, Canada; 9 Department of Anesthesia and Li Ka Shing Knowledge Institute, St. Michael’s Hospital, Toronto, Ontario, Canada; 10 Departments of Anesthesiology and Pain Medicine, Physiology, and Pharmacology and Toxicology, University of Toronto, Toronto, Ontario, Canada; 11 Département d’anesthésie, Institut de cardiologie de Montréal, Université de Montréal, Montréal, Québec, Canada; 12 Department of Anesthesia, St Paul’s Hospital, University of British Columbia, Vancouver, British Columbia, Canada; 13 Department of Anesthesiology, Weill Cornell Medical College, New York, New York; 14 Department of Anesthesia, Vancouver General Hospital, University of British Columbia, Vancouver, British Columbia, Canada; 15 Department of Anesthesia and Critical Care Medicine, Queen Elizabeth II Health Sciences, Dalhousie University, Halifax, Nova Scotia, Canada; 16 Department of Anesthesia, Jewish General Hospital, McGill University, Montreal, Québec, Canada; 17 St Michael’s Hospital, Toronto, Ontario, Canada; 18 Institut universitaire de cardiologie et de pneumologie de Québec - Université Laval, Québec, Québec, Canada; 19 Department of Anesthesia, University of Western Ontario, London, Ontario, Canada; 20 Department of Anesthesiology, Royal Columbian Hospital and Department of Anesthesiology, Pharmacology & Therapeutics, University of British Columbia, Vancouver, British Columbia, Canada; 21 Department of Anesthesiology and Pain Medicine, University of Toronto, Toronto, Ontario, Canada; 22 Department of Anesthesia, Queen’s University, Kingston, Ontario, Canada; 23 Department of Anesthesia, Sunnybrook Health Sciences Centre, Toronto, Ontario, Canada; 24 Department of Anesthesiology and Pain Medicine, University of Toronto, Toronto, Ontario, Canada; 25 Department of Anesthesia, University of Saskatchewan, Saskatoon, Saskatchewan, Canada; 26 Department of Anesthesia, University of Alberta, Mazankowski Alberta Heart Institute, Edmonton, Alberta, Canada; 27 Department of Medicine, Critical Care, McMaster University, Hamilton, Ontario, Canada; 28 Department of Medicine, Cardiology and Critical Care, McMaster University, Hamilton, Ontario, Canada; 29 Departments of Anesthesia and Perioperative Medicine and Medicine, Critical Care, University of Manitoba, Winnipeg, Manitoba, Canada

## Abstract

**Importance:**

Delirium is common after cardiac surgery and associated with adverse outcomes. Intraoperative benzodiazepines may increase postoperative delirium but restricting intraoperative benzodiazepines has not yet been evaluated in a randomized trial.

**Objective:**

To determine whether an institutional policy of restricted intraoperative benzodiazepine administration reduced the incidence of postoperative delirium.

**Design, Setting, and Participants:**

This pragmatic, multiperiod, patient- and assessor-blinded, cluster randomized crossover trial took place at 20 North American cardiac surgical centers. All adults undergoing open cardiac surgery at participating centers during the trial period were included through a waiver of individual patient consent between November 2019 and December 2022.

**Intervention:**

Institutional policies of restrictive vs liberal intraoperative benzodiazepine administration were compared. Hospitals (clusters) were randomized to cross between the restricted and liberal benzodiazepine policies 12 to 18 times over 4-week periods.

**Main Outcomes and Measures:**

The primary outcome was the incidence of delirium within 72 hours of surgery as detected in routine clinical care, using either the Confusion Assessment Method–Intensive Care Unit or the Intensive Care Delirium Screening Checklist. Intraoperative awareness by patient report was assessed as an adverse event.

**Results:**

During the trial, 19 768 patients (mean [SD] age, 65 [12] years; 14 528 [73.5%] male) underwent cardiac surgery, 9827 during restricted benzodiazepine periods and 9941 during liberal benzodiazepine periods. During restricted periods, clinicians adhered to assigned policy in 8928 patients (90.9%), compared to 9268 patients (93.2%) during liberal periods. Delirium occurred in 1373 patients (14.0%) during restricted periods and 1485 (14.9%) during liberal periods (adjusted odds ratio [aOR], 0.92; 95% CI, 0.84-1.01; *P* = .07). No patient spontaneously reported intraoperative awareness.

**Conclusions and Relevance:**

In intention-to-treat analyses, restricting benzodiazepines during cardiac surgery did not reduce delirium incidence but was also not associated with an increase in the incidence of patient-reported intraoperative awareness. Given that smaller effect sizes cannot be ruled out, restriction of benzodiazepines during cardiac surgery may be considered. Research is required to determine whether restricting intraoperative benzodiazepines at the patient level can reduce the incidence of postoperative delirium.

**Trial Registration:**

ClinicalTrials.gov Identifier: NCT03928236

## Key Points

**Question:**

Does an institutional policy restricting benzodiazepines during cardiac anesthesia reduce the incidence of postoperative delirium?

**Findings:**

In this cluster randomized crossover trial including 19 768 patients at 20 hospitals, there was no statistically significant difference in delirium when comparing restrictive with liberal benzodiazepine administration. No patients reported intraoperative awareness.

**Meaning:**

At the institutional level, both liberal and restrictive approaches to intraoperative benzodiazepine administration for cardiac anesthesia may be considered; research is required to determine whether restricting intraoperative benzodiazepines at the patient level can reduce the incidence of postoperative delirium.

## Teaser

This cluster randomized crossover trial evaluates the effect of restricting intraoperative benzodiazepine administration on incidence of postoperative delirium.

## Introduction

Postoperative delirium, a common complication of cardiac surgery, is associated with increased morbidity, mortality, and health care costs.^1^ Low-quality evidence suggests that patients receiving benzodiazepines in the intensive care unit (ICU) after cardiac surgery may be at elevated risk of delirium. Intensive care guidelines recommend avoiding benzodiazepines postoperatively.^2^ In contrast, due to the belief that they may protect against intraoperative awareness,^3^ benzodiazepines are administered in the operating room during cardiac surgery to 60% to 89% of patients.^3,4^ However, practice varies across centers and anesthesiologists.^3,4^

Individual patient efficacy trials help establish whether an intervention has the desired effect when applied to a selected population under optimal conditions following detailed protocols.^5^ Such trials often fail to determine how well the intervention or treatment works in routine clinical practice, where conditions vary. Pragmatic trials test interventions under the conditions and settings where they would be used should the trial support them.^5^

Cardiac surgery is performed in select institutions performing high volumes of surgery to reduce complications and increase efficiency. The surgical care of patients in these specialized centers is undertaken using standardized procedures that optimize outcomes, including standard perioperative care pathways.^6^ Because cardiac surgical care is organized using standard operating policies, a pragmatic way to test the effect of benzodiazepine use on outcomes is to vary the policy regarding intraoperative benzodiazepine use at a center, as if it were a standard care pathway. We undertook the Benzodiazepine-Free Cardiac Anesthesia (B-Free) trial to determine whether an institutional policy of restricted intraoperative benzodiazepine administration, compared to a policy of liberal use, would reduce the incidence of delirium after cardiac surgery. Our objective was to ascertain the effectiveness of restricting benzodiazepines in routine clinical care.

## Methods

### Study Design

The trial protocol is in Supplement 1. B-Free was a pragmatic, multiple-period, cluster randomized crossover trial conducted in 20 North American cardiac surgical centers. Details of the trial design have been published.^7^ We chose a cluster randomized design because our intervention—an institutional policy—was applied at the level of the hospital (ie, cluster). We incorporated multiple crossovers to gain statistical power,^8^ having shown that this was feasible without loss of adherence.^9^ We obtained research ethics board approval at participating centers before commencing the trial. In accordance with the Tri-Council Policy Statement,^10^ we obtained ethical approval for waived individual patient consent. We notified patients (through a letter of information) that their hospital was studying alternate policies regarding cardiac anesthesia medications, that anonymized data were being collected from their medical record, and that they could opt out of having their information included.

### Participants

We included North American cardiac surgical centers if 95% or more of cardiac anesthesiologists agreed to treat patients according to allocated benzodiazepine policy and patients were assessed for postoperative delirium at least every 12 hours after cardiac surgery using either the Confusion Assessment Method-ICU^11^ or the Intensive Care Delirium Screening Checklist.^12^ We included patients in analyses if they underwent cardiac surgery at a participating center during the trial; percutaneous procedures and secondary operations during the same admission were excluded. Research staff identified patients through surgical rosters and postoperative ICU admissions. The trial took place from November 18, 2019, to December 11, 2022. On 2 occasions, we placed the trial on hold due to the impact of the COVID-19 pandemic on cardiac surgical activities. Sites resumed the trial when feasible based on pandemic impact.

### Randomization and Blinding

An independent statistician randomized hospitals to use 1 of 2 benzodiazepine policies for each of twelve to eighteen 4-week crossover periods, with randomization blocked in periods of 2 to minimize period effects (eFigure 1 in Supplement 2). A randomization schedule for the proposed sample size, including additional sites and periods to account for unforeseen circumstances, such as withdrawals and trial extensions, was created in advance of the beginning of the trial. Sites were notified of the subsequent period’s policy during the last week of the preceding period. Cardiac anesthesiologists were not blinded as this was infeasible. Clinical staff responsible for postoperative care and delirium assessment were not notified that the trial was occurring, nor of the randomized allocation. Patients and statisticians were blinded to intraoperative benzodiazepine policy.

### Procedures

We compared 2 minimal-risk, hospital-based, cardiac anesthesia policies. The restricted benzodiazepine policy mandated no benzodiazepines during cardiac surgery. The liberal benzodiazepine policy mandated the administration of at least 0.03 mg/kg ideal body weight (60 kg in female patients, 70 kg in male patients) midazolam equivalent during cardiac surgery. Both policies allowed discretionary deviations when the treating anesthesiologist believed benzodiazepines were mandated or contraindicated (Intervention Arm Policies in eMethods in Supplement 2). Cardiac anesthesiologists were notified of policy allocation by email and signage on anesthetic machines. All trial data were obtained as part of routine clinical care and extracted from hospital records. Patients were monitored until hospital discharge.

### Outcomes

The primary outcome was postoperative delirium, as detected in routine care, up to 72 hours after surgery using either the Confusion Assessment Method-ICU^11^ or the Intensive Care Delirium Screening Checklist^12^ (Outcome Definitions in eMethods in Supplement 2). Secondary outcomes included ICU length of stay, hospital length of stay, and all-cause in-hospital mortality (Outcome Definitions in eMethods in Supplement 2). We collected intraoperative awareness by patient self-report as an adverse event. Post hoc, we evaluated the effect of the restrictive benzodiazepine policy on the total number of assessments documenting the presence of delirium within 72 hours after surgery.

### Statistical Analysis

A priori, we determined that to have greater than 80% statistical power to demonstrate a 15% relative risk reduction in the incidence of delirium (assuming an incidence of 13.9%, an intracluster correlation coefficient of 0.02, and a type I error of 5%), we would need to include 16 hospitals with an average annual case volume of 1000 patients completing twelve 4-week crossover periods. Due to concerns about the pandemic impact on cardiac surgery case volumes and uncertainty in the assumed intracluster correlation coefficient, we re-estimated the sample size without unblinding during our interim analysis. This interim analysis to assess efficacy and safety was completed by an independent data safety monitoring board using a modified Haybittle-Peto rule after enrolling 70% of patients.

Trial sample size re-estimation is described in the eMethods in Supplement 2. Based on data from 11 222 patients, we determined that the original sample size would be underpowered due to a smaller projected cluster size and a higher-than-assumed intracluster correlation coefficient. To maintain statistical power greater than 80%, we increased the number of periods per hospital where feasible, with a final sample size of 9 hospitals completing 18 crossover periods, 2 hospitals completing 17 crossover periods, and 9 hospitals completing 12 crossover periods.

The primary analyses followed the intention-to-treat principle, with participants analyzed according to the institutional policy in place when they underwent surgery. Analyses took place at the individual patient level but accounted for clustering. We included all patients fulfilling inclusion criteria in analyses, including those who were not assessed for delirium during the 72 hours following surgery, assuming these patients did not have delirium. To analyze the primary, secondary, and post hoc outcomes, we used a logistic mixed model for binary outcomes, a linear mixed model for continuous outcomes, and a mixed negative binomial model for nonnormal continuous outcomes (ie, count data), including a fixed-effects term for period as categorical, and using random effects to account for within-period intracluster correlation coefficient and an autocorrelation matrix to introduce an exponential decay in the strength of correlation over time. We adjusted for the following baseline covariates: age, sex, emergency surgery, history of heavy alcohol consumption, and history of home benzodiazepine use in all models. We assessed heterogeneity of the treatment effect across centers using an interaction term. We performed prespecified subgroup analyses of the primary outcome for age and preoperative history of benzodiazepine and/or heavy alcohol use. Post hoc, we performed subgroup analyses comparing centers using the Confusion Assessment Method-ICU for delirium assessment with those using the Intensive Care Delirium Screening Checklist and evaluated the effect of season, number of periods completed, and local case volume. We tested for subgroup effects by including an interaction term in the primary analysis model.

We completed several post hoc analyses. To evaluate the effect of nonadherence to allocated policy, we completed a sensitivity analysis excluding patients allocated to the restricted policy who received intraoperative benzodiazepines and patients allocated to the liberal policy who did not receive intraoperative benzodiazepines. To evaluate whether preoperative benzodiazepine administration impacted our results, we completed an analysis excluding patients in the restricted periods who received benzodiazepines within 24 hours before cardiac surgery, within the primary analysis population and the population including only those treated according to allocated intraoperative policy. To evaluate the impact of patients with missing delirium assessments, we completed a sensitivity analysis excluding them.

We report the standardized mean difference (SMD) between treatment allocations for baseline characteristics and surgical and anesthetic details. For binary outcomes, we report adjusted odds ratios (aORs). For continuous outcomes, we report mean differences. For count data, we report adjusted relative risks (aRRs). For all outcomes, we report 95% CIs. We considered an SMD greater than 0.10 to indicate covariate imbalance^13^ and a 2-sided *P* value <.05 to indicate statistical significance. We did not adjust for multiplicity. We performed analyses using SAS version 9.4 for UNIX (SAS Institute).

## Results

**Figure 1.**
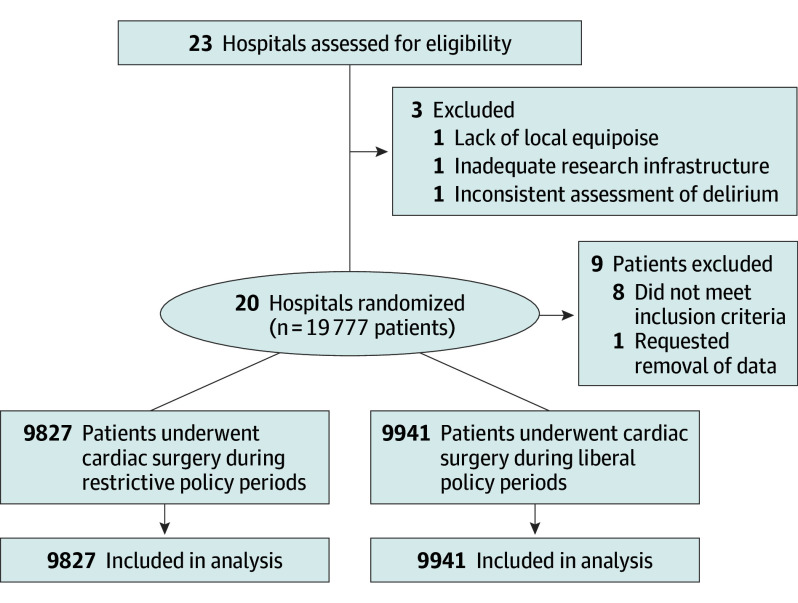
Recruitment, Policy Allocation, and Patient Flow in the B-Free Trial

We approached 23 potentially eligible hospitals; 20 were randomized. The trial took place from November 18, 2019, until December 11, 2022. During the trial, 19 769 patients fulfilled inclusion criteria, and 1 patient requested removal of their data. The mean (SD) age of the 19 768 included patients was 65 (12) years; 14 528 patients were male (73.5%) and 5240 (26.5%) were female. Of those included in analyses, 9827 underwent cardiac surgery during restrictive benzodiazepine periods and 9941 underwent cardiac surgery during liberal benzodiazepine periods (Figure 1). Overall, 18 901 patients (95.6%) were assessed for delirium (9380/9827 [95.5%] during restricted periods and 9521/9941 [95.8%] during liberal periods; SMD, 0.02) (eTable 4 in Supplement 2). Table 1 summarizes patient and surgical characteristics. Cluster characteristics and surgical procedures are described in eTables 5 and 6 in Supplement 2. A total of 1549 patients (7.8%) underwent emergency surgery. A minority had a history of home benzodiazepine use (1389 [7.2%]) or heavy alcohol use (933 [4.7%]) or had delirium at the time of surgery (95 [0.5%]). Benzodiazepines were given within 24 hours before surgery to 3172 patients (16.0%). Most patients (18 806 [95.1%]) underwent cardiac surgery with the use of cardiopulmonary bypass; mean (SD) cardiopulmonary bypass time was 116 (58) minutes.

**Table 1.** Baseline Characteristics, Surgical Details, Anesthetic Management, and Sedation in the Intensive Care Unit

Characteristic	No. (%)		Standardized mean difference
	Restricted benzodiazepine policy (n = 9827)	Liberal benzodiazepine policy (n = 9941)	
Baseline characteristics and surgical details			
Age, mean (SD), y	65.0 (11.7)	64.9 (11.8)	<0.01
Sex			
Male	7203 (73.3)	7325 (73.7)	<0.01
Female	2624 (26.7)	2616 (26.3)	<0.01
Emergency procedure	747 (7.6)	802 (8.1)	0.02
History of home benzodiazepine use	664 (7.0)	725 (7.5)	0.02
History of heavy alcohol use	448 (4.6)	485 (4.9)	0.01
Received benzodiazepines within 24 h prior to surgery	1436 (14.6)	1736 (17.5)	0.08
Delirium at the time of surgery	42 (0.4)	53 (0.5)	0.01
Surgical procedure			
Single valve repair/replacement	1725 (17.6)	1734 (17.4)	<0.01
Isolated coronary artery bypass graft	4765 (48.5)	4896 (49.3)	0.02
Other cardiac surgery	3337 (34.0)	3311 (33.3)	0.01
Use of cardiopulmonary bypass	9366 (95.3)	9440 (95.0)	0.02
Cardiopulmonary bypass time, mean (SD), min	116 (58)	117 (58)	<0.01
Aortic cross-clamp time, mean (SD), min	88 (47)	88 (48)	<0.01
Intraoperative anesthetic medications			
Received intraoperative benzodiazepines	899 (9.1)	9268 (93.2)	3.11
Dose in all patients, median (IQR), mg^a^	0.0 (0.0-0.0)	4.0 (2.5-5.0)	1.75
Dose in patients receiving intraoperative benzodiazepine, mean (SD), mg^a^	4.1 (3.0)	4.1 (2.2)	0.03
Received intraoperative opioids	9789 (99.6)	9895 (99.6)	0.01
Dose in patients receiving intraoperative opioids, mean (SD), µg^b^	1351 (905)	1322 (916)	0.03
Received intraoperative propofol	9622 (97.9)	9560 (96.2)	0.10
Dose in patients receiving intraoperative propofol bolus, median (IQR), mg	100 (50-140)	80 (50-120)	0.27
Dose in patients receiving intraoperative propofol infusion, median (IQR), mg	424 (115-830)	391 (91-788)	0.03
Total dose in patients receiving any intraoperative propofol, median (IQR), mg	500 (185-929)	445 (148-859)	0.06
Received intraoperative ketamine	3112 (31.7)	2739 (27.6)	0.09
Dose in patients receiving intraoperative ketamine, mean (SD), mg	52.6 (58)	51.5 (35)	0.02
Received intraoperative etomidate	425 (4.3)	293 (3.0)	0.07
Dose in patients receiving intraoperative etomidate, mean (SD), mg	15 (7)	14 (6)	0.14
Received intraoperative dexmedetomidine	1051 (10.7)	927 (9.3)	0.05
Dose in patients receiving intraoperative dexmedetomidine, median (IQR), µg	96 (50-167)	100 (55-172)	0.01
Medications received in the intensive care unit up to 72 h after surgery			
Postoperative benzodiazepines	1012 (10.3)	1102 (11.1)	0.03
Postoperative dexmedetomidine	1374 (14.0)	1388 (14.0)	<0.01

^a^ Midazolam equivalents (detailed in eTable 3 in Supplement 2).

^b^ Fentanyl equivalents (detailed in eTable 2 in Supplement 2).

Table 1 details medications given for anesthesia and within 72 hours postoperatively in the ICU. During restricted periods, clinicians adhered to assigned policy in 8928 patients (90.9%) compared to 9268 (93.2%) of patients during liberal periods. Benzodiazepines were administered intraoperatively to 899 patients (9.1%) treated during restricted periods and 9268 (93.2%) treated during liberal periods. Among patients who received intraoperative benzodiazepines, the mean (SD) dose was 4.1 (2.3) mg in midazolam equivalents.

Patients in the restrictive arm received higher doses of etomidate and propofol; this difference was not meaningful clinically (mean [SD] etomidate, 15 [7] mg compared to 14 [6] mg; SMD, 0.14; median [IQR] propofol, 100 [50-140] mg compared to 80 [50-120] mg; SMD, 0.27). Beyond this difference, nonbenzodiazepine anesthetic management was similar between groups. Overall, 2410 patients (10.7%) received benzodiazepines and 2762 (14.0%) received dexmedetomidine within 72 hours after cardiac surgery, with no significant difference between restricted and liberal policy periods.

**Figure 2.**
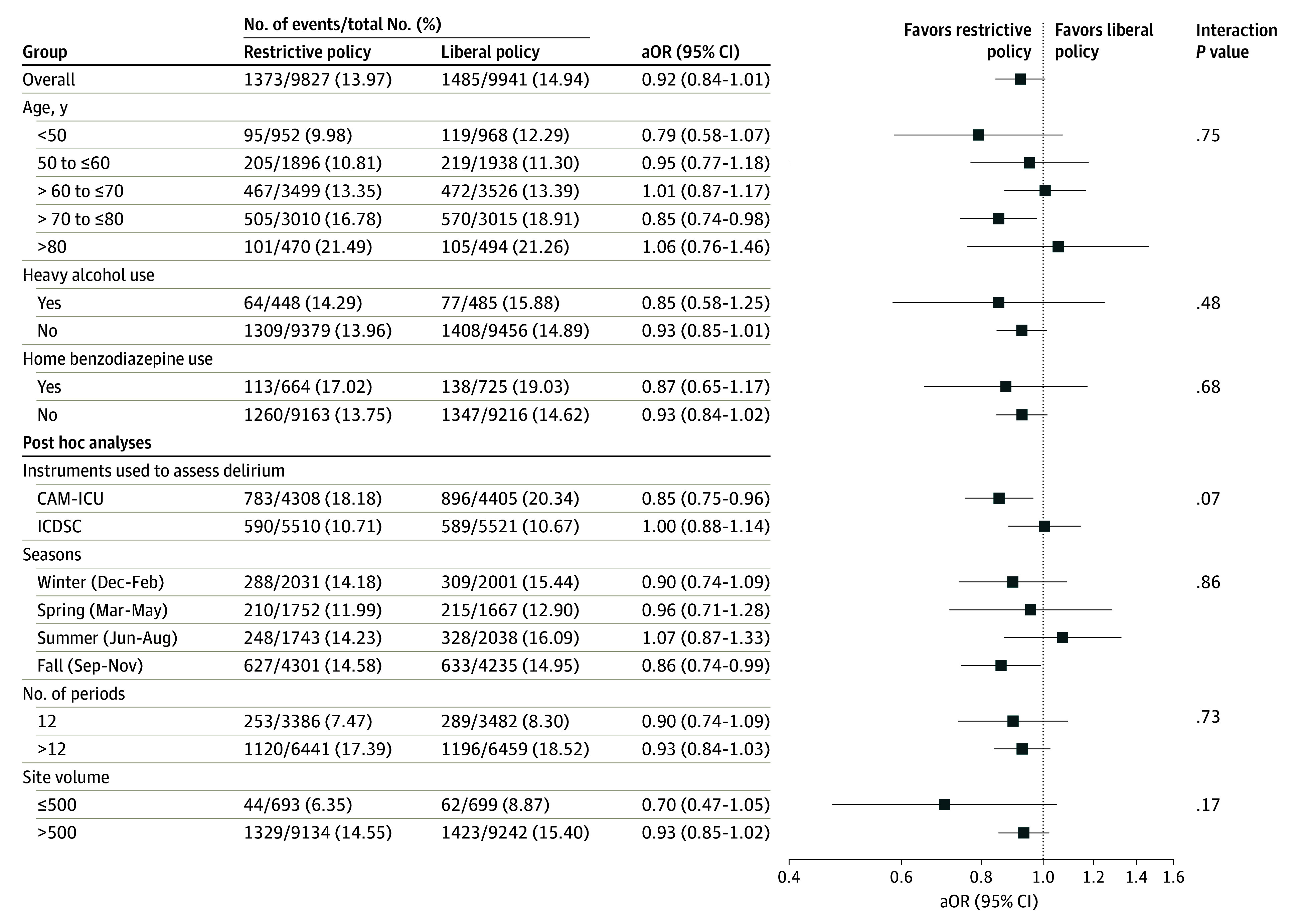
A Priori–Specified Subgroup Analyses for Delirium up to 72 Hours Adjusted odds ratios (aORs) were adjusted for age, sex, urgency of surgery, history of heavy alcohol consumption, and history of home benzodiazepine use. CAM-ICU indicates Confusion Assessment Method–Intensive Care Unit; ICDSC, Intensive Care Delirium Screening Checklist.

Delirium occurred within the first 72 hours after cardiac surgery in 1373 patients (14.0%) during restrictive benzodiazepine periods and in 1485 patients (14.9%) during liberal benzodiazepine periods (aOR, 0.92; 95% CI, 0.84-1.01; *P* = .07) (Table 2). The treatment effect across centers was similar (eFigure 2 and eTable 7 in Supplement 2). In prespecified subgroup analyses, the effect of the intervention on the primary outcome was consistent (Figure 2).

**Table 2.** Effect of Restricted vs Liberal Intraoperative Benzodiazepine Policy on the Primary and Secondary Outcomes

Outcome	Restricted benzodiazepine policy (n = 9827)	Liberal benzodiazepine policy (n = 9941)	Summary estimate (95% CI)	*P* value	ICC	Absolute difference of proportions (95% CI)
Delirium up to 72 h after cardiac surgery, No. (%)^a^	1373 (14.0)	1485 (14.9)	aOR, 0.92 (0.84 to 1.01)	.07	0.06	0.97 (0.01 to 2.0)
Intensive care length of stay, LSM (95% CI), d^a^	3.5 (2.8 to 4.5)	3.5 (2.7 to 4.5)	Mean difference, 0.04 (−0.12 to 0.27)	.68	0.09	NA
Hospital length of stay, LSM (95% CI), d^a^	12.6 (11.5 to 14.0)	12.7 (11.5 to 14.1)	Mean difference, −0.05 (−0.53 to 0.31)	.81	0.05	NA
In-hospital mortality, No. (%)^a^	298 (3.0)	272 (2.7)	aOR, 1.13 (0.95 to 1.34)	.16	0.008	NA

Abbreviations: aOR, adjusted odds ratio; ICC, intracluster correlation coefficient; LSM, least squares mean; NA, not applicable.

^a^ Analyses adjusted for age (years), sex, urgency of surgery (emergency vs elective), history of heavy alcohol consumption, and history of home benzodiazepine use.

Table 2 presents secondary and safety outcomes. Length of stay in the ICU was similar during restrictive and liberal policy periods (adjusted mean difference, 0.04 days; 95% CI, −0.12 to 0.27; *P* = .68). Hospital length of stay and in-hospital mortality were similar between arms. No patient reported intraoperative awareness.

### Post Hoc Analyses

During the 72 hours after surgery, patients who had surgery during restricted policy periods had fewer assessments documenting delirium presence compared to those who had surgery during liberal policy periods (aRR, 0.88; 95% CI, 0.79-0.97; *P* = .02) (Table 3; eFigure 3 in Supplement 2). When considering only patients treated according to the intraoperative benzodiazepine policy in place at the time of surgery, 1219 patients (13.7%) in the restrictive arm developed delirium compared to 1372 (14.8%) in the liberal arm (aOR, 0.90; 95% CI, 0.82-0.99) (Table 3). The results were consistent when excluding patients treated during restrictive periods who received benzodiazepines within 24 hours before cardiac surgery, within both the primary analysis population and the population limited to those treated according to intraoperative benzodiazepine policy (Table 3). Removing missing assessments did not affect results (eTable 8 in Supplement 2). No significant subgroup effects for method of delirium assessment, season, number of periods completed, or local case volume were detected (Figure 2).

**Table 3.** Post Hoc and Sensitivity Analyses^a^

Outcome	Restricted benzodiazepine policy	Liberal benzodiazepine policy	Summary estimate (95% CI)	*P* value
**Primary analysis population**				
No.	8928	9268	NA	NA
No. of assessments documenting delirium presence up to 72 h after cardiac surgery, median (IQR)	1 (1-3)	2 (1-3)	aRR, 0.88 (0.79-0.97)	.02
**Population limited to patients treated according to intraoperative policy**				
No.	8928	9268	NA	NA
Delirium up to 72 h after cardiac surgery, No. (%)	1219 (13.7)	1372 (14.8)	aOR, 0.90 (0.82-0.99)	.02
**Population removing patients in the restrictive arm who received benzodiazepines within 24 h before surgery**				
No.	8391	9941	NA	NA
Delirium up to 72 h after cardiac surgery, No. (%)	1149 (13.7)	1485 (14.9)	aOR, 0.88 (0.81-0.97)	.01
**Population limited to patients treated according to intraoperative policy and removing patients in the restrictive arm who received benzodiazepines within 24 h before surgery**				
No.	7659	9268	NA	NA
Delirium up to 72 h after cardiac surgery, No. (%)	1024 (13.4)	1372 (14.8)	aOR, 0.87 (0.79-0.96)	.005

Abbreviations: aOR, adjusted odds ratio; aRR, adjusted risk ratio; NA, not applicable.

^a^ Analyses adjusted for age, sex, urgency of surgery (emergency vs elective), history of heavy alcohol consumption, and history of home benzodiazepine use.

## Discussion

In this cluster randomized crossover trial of 19 768 patients at 20 hospitals, an institutional policy of restrictive vs liberal intraoperative benzodiazepine use did not reduce the incidence of delirium within 72 hours of cardiac surgery. Based on the width of the 95% CIs, the data were consistent with a treatment effect (ie, the difference in the adjusted odds of delirium at 72 hours between the restricted and liberal periods) ranging from 16% lower to 1% higher, which was smaller than the 15% relative risk reduction that our trial was powered to detect. In post hoc analyses, a restrictive benzodiazepine policy decreased the number of assessments documenting delirium presence and, when considering only patients treated according to allocated intraoperative policy, the adjusted odds of delirium were reduced by 10% (95% CI, 0.82-0.99). A further reduction in delirium occurred when considering only patients in the restrictive arm who did not receive benzodiazepines within 24 hours before surgery, regardless of whether the restrictive intraoperative benzodiazepine policy was applied.

Delirium is a common and potentially preventable clinical syndrome that occurs after an acute insult.^1^ Patients undergoing cardiac surgery are at high risk of delirium, with an incidence that ranges from 10% to 50% depending on the method and timeframe of assessment.^14,15^ The lived experience of delirium for patients and their families has been shown to be associated with the development of posttraumatic stress syndrome.^16^ Delirium is associated with loss of independence, increased morbidity and mortality, and health care resource consumption.^1,15^ In the longer term, incident delirium has been associated with cognitive decline and dementia.^1,15^ Because of its impact on patients and systems, researchers and clinicians have identified the prevention and treatment of delirium after cardiac surgery as a research priority.^17,18^ A meta-analysis^19^ of 4 randomized clinical trials found that multicomponent nonpharmacologic strategies reduced the incidence of delirium (220 events; OR, 0.56; 95% CI, 0.42-0.76; *I*^2^, 0%). No other intervention has been found to consistently prevent or treat delirium. To our knowledge, no trial has evaluated the effect of restricting intraoperative benzodiazepines, whether at the patient or institutional level, on postoperative delirium.

Many clinicians believe benzodiazepines increase the risk of delirium, and this is reflected in guideline recommendations. The Society for Critical Care Medicine recommends against benzodiazepines for sedation in critically ill adults requiring mechanical ventilation. This recommendation stems from delirium data from 4 randomized clinical trials (1007 patients total) comparing benzodiazepines with dexmedetomidine (225 events; RR 1.23; 95% CI, 0.92-1.67; low-quality evidence).^2^ Based on observational evidence and expert opinion, the American Geriatric Society recommends that benzodiazepines be avoided in adults older than 65 years because of their association with delirium, cognitive decline, and falls.^20^ Expert opinion informs the American Society of Anesthesiology Perioperative Brain Health Initiative’s recommendation to avoid benzodiazepines in patients older than 65 years due to their association with postoperative cognitive decline and delirium.^21^

Despite guideline recommendations, most cardiac anesthesiologists use benzodiazepines intraoperatively in patients at risk of delirium.^3,4,22^ The persistent intraoperative administration of benzodiazepines may be due to limitations in the existing evidence. A systematic review and meta-analysis^23^ of randomized clinical trials found the risk of delirium after cardiac and noncardiac surgery ranged from 10% lower to 127% higher when perioperative benzodiazepines were given (n = 1352; 284 events; RR, 1.43; 95% CI, 0.9-2.3; *P* = .13). The confidence in this effect estimate was very low due to the high risk of bias of included studies (ie, most were small and single-center trials), heterogeneity in effect, and confidence intervals that included both possible benefit and harm.^23^

While our trial did not identify an increase in the risk of postoperative delirium with intraoperative benzodiazepine exposure, in contrast to the available evidence, it was rigorous and powered to detect a 15% relative risk reduction in delirium. The observed effect size was smaller (8% to 13%) and the trial was not powered to detect an effect of this magnitude. The confidence interval around the primary effect estimate includes both the possibility of no important effect and a small reduction in delirium with benzodiazepine restriction.

To our knowledge, no meta-analysis of randomized clinical trials has demonstrated that benzodiazepines have a significant relationship with delirium. This is likely because delirium is multifactorial and the relative contribution of benzodiazepine exposure to the development of delirium is small. At the individual patient level, the importance of a small relative risk reduction is unclear. Considering the event rates observed in our sensitivity analysis evaluating only patients treated according to allocated intraoperative policy, benzodiazepines would need to be restricted in 91 patients to prevent 1 case of postoperative delirium.

### Strengths and Limitations

Strengths of our trial include a large and representative sample and greater than 90% adherence to the policies evaluated. We used an innovative and pragmatic trial design integrating our research question into routine clinical care, with results that are generalizable to the broad population of adults undergoing cardiac surgery. However, this pragmatic approach did not allow us to control for perioperative benzodiazepine administration, a limitation that may have diminished the observed effect size, as suggested by sensitivity analyses limited to patients who did not receive preoperative benzodiazepines. Our pragmatic approach similarly prevented formal assessment for intraoperative awareness; some cases of intraoperative awareness may not have been detected as we relied on patient self-report.^24^ Further, the observed delirium incidence was lower than that reported in many studies,^15^ which could be explained by the short observation period and the proportion of patients that could not be evaluated due to sedation.^25,26^ There was also a wide range of delirium incidence across centers, although the effect of the restrictive benzodiazepine policy was similar.

## Conclusions

In intention-to-treat analyses, a policy restricting the use of benzodiazepines during cardiac surgery did not reduce delirium incidence but was also not associated with an increase in patient-reported intraoperative awareness. Given that smaller effect sizes cannot be ruled out, restricting benzodiazepines during cardiac surgery may be considered. Research is required to determine whether restricting intraoperative benzodiazepines at the patient level can reduce the incidence of postoperative delirium.

## Notes

Supplement 1. Trial protocol and statistical analysis plan

Supplement 2. eAppendix. Study groups eMethods eTable 1. Assumed, Observed, and Recalculated Sample Size Calculations eTable 2. Conversion of opioids to Fentanyl equivalents eTable 3. Conversion of benzodiazepines to Midazolam equivalents eFigure 1. Randomization schedule for participating clusters eTable 4. Delirium assessments by treatment allocation by day eTable 5. Cluster level Characteristics eTable 6. Detailed surgical characteristics eFigure 2. Results for the incidence of delirium up to 72 hours after cardiac surgery by center eTable 7. Results for the incidence of delirium up to 72 hours after cardiac surgery by center reporting which scale used eFigure 3. Number of assessments documenting the presence of delirium per 12 hours after cardiac surgery by intervention allocation eTable 8. Sensitivity analysis excluding patients who were not assessed for delirium

Supplement 3. B-Free Investigators and the Canadian Perioperative Anesthesia Clinical Trials Group

Supplement 4. Data sharing statement
