# Supplementary material for: Benzodiazepine-Free Cardiac Anesthesia for Reduction of Postoperative Delirium: A Cluster Randomized Crossover Trial
Source: JAMA Surg. 2025 Jan 29;160(3):286–94. doi: 10.1001/jamasurg.2024.6602 (PMC11780505; doi:10.1001/jamasurg.2024.6602)
Supplement: Supplement 1. — Trial protocol and statistical analysis plan [file jamasurg-e246602-s001.pdf]

Supplemental Material

This supplement contains the following items:

1. Original protocol, final protocol, summary of changes

Benzodiazepine-free Cardiac Anesthesia

for Reduction of Postoperative Delirium

(B-Free)

NOTE TO FILE

DATE: January 24, 2024

RE: Benzodiazepine-free Cardiac Anesthesia for Reduction of Postoperative Delirium (B-Free) Trial

A draft Protocol was written in November 2018. This draft version (v1.0, dated 2018-11-25) was not finalized, as the investigator continued to refine content throughout study start-up. Due to the changes in exclusion criteria, sample size, adding the process for data collection, further defining the different policies, and study outcomes, this draft version of the Protocol was abandoned, and a new Protocol was written and finalized (v2.0, dated 2019-02-20). This version of the Protocol was submitted to the Research Ethics Board and changes were requested.

A revision was made to Protocol v2.0 to incorporate feedback. This resulted in the revised Protocol v3.0 (dated 2019-04-25) that was the first protocol version granted ethical approval. The changes made from v2.0 to v3.0 included: updating the study objectives to include postoperative delirium assessments up to 72 hours, updating the inclusion criteria to hospitals with a minimum of 500 cardiac cases per year, specifying the acceptable assessment methods for delirium, including the expected number of hospitals and number of patients, further defining the study intervention to describe exceptions for benzodiazepine use, and updating the primary outcome to specify the percentage of a patients with delirium assessed during the initial 72 hours following cardiac surgery.

Name of Principal Investigator: Dr. Jessica Spence

DocuSigned by:

Jessica Spence

Signature: Signer Name: Jessica Spence

Signing Reason: I approve this document

Signing Time: 2024-01-24 | 3:01:51 PM EST

Date: 2024-01-24 | 3:01:54 PM EST

E06CC11CE8984853983B0EAD7101A574

Clinical Trial Protocol

Benzodiazepine-free Cardiac Anesthesia for Reduction of Postoperative Delirium (B-Free)

Final Version 3.0
2019-04-25

This protocol is the confidential intellectual property of the Sponsor and B-Free Steering Committee.
Acceptance implies an agreement not to disclose information contained herein that is not otherwise publicly available, with the exception that it may be disclosed to an Research Ethics Board (REB) for the purpose of obtaining approval to conduct the study.
The REB is requested and expected to maintain confidentiality.
This document may not be used or published without the consent of consent of the Sponsor or Steering Committee.

INVESTIGATOR'S AGREEMENT

I, ____, the investigator, have examined this protocol:

B-Free

and I have fully discussed the objectives of this trial and the contents of this amended protocol with the B-Free Coordinating Center representative(s) from the Population Health Research Institute.

I agree to conduct the study according to this protocol and to comply with its requirements, subject to ethical and safety considerations.

I agree to comply with the International Council for Harmonisation Tripartite Guideline on Good Clinical Practice (GCP) and applicable regulations/guidelines and all locally applicable laws.

I agree to ensure that the confidential information contained in this document will not be used for any purpose other than the evaluation or conduct of the clinical investigation without the prior written consent of the sponsor.

Investigator Name: ____________________________________

Investigator Signature: ______________________ **Date:** ______________

PROTOCOL APPROVAL

Signature: Date:

2019-04-25

Jessica Spence, MD FRCPC
McMaster University, Hamilton Health Sciences
Hamilton, ON Canada

TRIAL LEADERSHIP AND MANAGEMENT

**Coordinating Center**	Population Health Research Institute (PHRI)<br>A Joint Institute of Hamilton Health Sciences and McMaster University<br><br>David Braley Cardiac, Vascular and Stroke Research Institute<br>Hamilton General Hospital<br>237 Barton Street East<br>Hamilton, ON L8L 2X2 Canada<br><br>B-FREE@phri.ca
**Principal Investigator**	**Jessica Spence MD FRCPC**<br>McMaster University, Hamilton Health Sciences<br>Hamilton, ON Canada
**Co-Principal Investigator**	**Eric Jacobsohn MBChB MPHE FRCPC**<br>University of Manitoba,<br>Winnipeg, MB Canada
**Steering Committee Chair**	**Stuart Connolly MD FRCPC**<br>McMaster University, Hamilton Health Sciences<br>Population Health Research Institute (PHRI)<br>Hamilton, ON Canada
**Study Sponsor**	Hamilton Health Sciences, through its Population Health Research Institute<br>237 Barton Street East<br>Hamilton, ON L8L 2X2 Canada

TABLE OF CONTENTS

ABBREVIATIONS ... 6
STUDY SYNOPSIS ... 7
1. Introduction and Rationale ... 9
2. Objectives ... 12
 2.1 Primary Objective ... 12
 2.2 Secondary Objectives ... 12
3. Study Design ... 12
 3.1 Type of Study ... 12
 3.2 Expected Number of Clusters and Patients ... 12
 3.3 Method of Intervention Allocation ... 13
 3.4 Methods for Protecting Against Bias ... 13
 3.5 Duration of Each Intervention Period ... 13
 3.6 Duration of Follow-up ... 13
4. Criteria for Inclusion of a Hospital ... 13
5. Benzodiazepine policies to be studied ... 13
6. Study Outcomes ... 14
 6.1 Primary Outcome ... 14
 6.2 Secondary Outcomes ... 14
 6.3 Outcome Definitions ... 14
7. Statistical Considerations ... 15
 7.1 Analysis Population ... 15
 7.2 Statistical Methods ... 15
 7.3 Planned Subgroup Analyses ... 15
 7.4 Interim Analysis ... 15
 7.5 Sample Size Calculation ... 15
8. Data Management ... 16
9. Study Organization ... 16
10. Ethical Standards ... 16
 10.1 Ethical Considerations ... 16
 10.2 Waiver of Individual Patient Consent ... 17
 10.3 Ethics Review and Approval ... 17
11. Investigator and Administrative Requirements ... 17

11.1 Investigator Responsibilities ... 17
12. Ownership of Data and Use of Study Results ... 18
13. Publication Policy ... 18
REFERENCES ... 19

ABBREVIATIONS

CAM-ICU	Confusion Assessment Method-ICU
CI	Confidence Interval
CNS	Central Nervous System
CPB	Cardiopulmonary Bypass
DSMB	Data Safety Monitoring Board
eCRF	Electronic Case Report Form
EMR	Electronic Medical Record
FDA	United States Food and Drug Administration
GABA	Gamma-aminobutyric Acid
GCP	Good Clinical Practice
ICC	Intra-cluster Correlation Coefficient
ICDSC	Intensive Care Delirium Screening Checklist
ICH	International Council for Harmonisation
ICU	Intensive Care Unit
IEC	Independent Ethics Committee
IPC	Interperiod Correlation Coefficient
IRB	Institutional Review Board
LOS	Length of Stay
PHRI	Population Health Research Institute
PRE	Government of Canada Panel on Research Ethics
REB	Research Ethics Board
SCCM	Society for Critical Care Medicine
TCPS	Tri-Council Policy Statement

STUDY SYNOPSIS

**Title**	Benzodiazepine-free cardiac anesthesia for the reduction of postoperative delirium (B-Free)
**Study Objectives**	The primary objective of the B-Free trial is to evaluate the impact of an institutional policy of limited benzodiazepine use during cardiac surgery, as compared to a policy of liberal benzodiazepine use during cardiac surgery, on the incidence of delirium during the initial 72 hours after cardiac surgery.<br><br>The secondary objectives are to evaluate the impact of these policies on:<br>1. ICU length of stay (LOS)<br>2. Hospital LOS<br>3. All cause in-hospital mortality
**Study Design**	A multi-centre, randomized cluster crossover trial.
**Hospital Eligibility:**	1. Major surgical center with a minimum of 500 cases of cardiac surgery per year<br>2. Equipoise by the hospital physicians regarding the use of benzodiazepines during surgery (≥ 95% of hospital cardiac anesthesia group agrees to manage patients as per the benzodiazepine policy in place during a given crossover period)<br>3. Patients are routinely assessed for postoperative delirium at least once every 12 hours during the initial 72 hours after cardiac surgery as a part of routine clinical care using either the Confusion Assessment Method-ICU (CAM-ICU) or the Intensive Care Delirium Screening Checklist (ICDSC).
**Total number of hospitals (clusters)**	16 hospitals, each with an average annual case volume of 1,000 patients, will be included in the trial.
**Expected number of subjects**	Approximately 16,000 adult patients undergoing cardiac surgery.
**Study Intervention**	The 'Limited Benzodiazepine Policy' consists of the following:<br>1. No routine use of any intraoperative benzodiazepines.<br>2. Accepted benzodiazepine use in the case of seizure, alcohol withdrawal, severe anxiety, history of awareness during anesthesia, or known benzodiazepine dependence.<br>3. Accepted benzodiazepine use in patients who are hemodynamically unstable and/or have cardiac anatomy that puts them at high risk of developing ischemia on induction of anesthesia using other agents.
**Study Comparator**	The 'Liberal Benzodiazepine Policy' consists of the following:<br>1. Administration of benzodiazepine as per clinical guidelines but no lower than 0.03 mg/kg (ideal body weight midazolam equivalent)

	to all patients undergoing cardiac surgery. Any benzodiazepine may be used.<br>2. Accepted avoidance of benzodiazepine use in patients who have contraindications to the administration of these medications (i.e. documented allergy, previous adverse reaction to benzodiazepine).
**Primary Outcome**	The primary study outcome is the percentage of patients with delirium assessed during the initial 72 hours following cardiac surgery.
**Statistical Analysis**	The primary analysis will be based on the intention to treat principle, all patients (aged ≥18 years) undergoing cardiac surgery will be included in the analysis treated during a period regardless of whether or not they were managed according to the policy in place during the period. Analyses will be carried out comparing event rates in patients managed during the benzodiazepine liberal and limited arms). The primary and secondary outcomes will be estimated between the treatment groups using a hierarchical mixed model for binary outcomes (i.e. GLIMMIX) adjusted for cluster and cluster-by-period as random effects terms (Turner et al., 2007). A sensitivity analysis will be conducted to evaluate the primary outcome in patients managed per protocol. The odds ratios and 95% confidence intervals will be reported. Statistical significance will be claimed if the p value is less than 0.05 for the primary outcome for treatment effectiveness.<br>Carryover effects will be assessed for the primary outcomes using tests for interactions between periods and treatment effects using hierarchical mixed models.
**Duration of Study Period (per cluster)**	Each hospital (cluster) will participate in the trial for twelve, four-week crossover periods.

1. INTRODUCTION AND RATIONALE

1.1 Delirium is a Serious Complication of Cardiac Surgery

Delirium is an acute disorder of cognition and attention that has been likened to 'acute brain failure.'[1] It is the most common surgical complication in older adults,[2,3] affecting up to 50% of post-operative patients aged 65 years and older.[1,4] Delirium has significant consequences for patients, families, and communities, both in the short- and long-term. Patients who experience delirium after surgery have longer hospital stays, and are more likely to be readmitted within 30-days or discharged to an institution.[5] Those who get post-operative delirium are also significantly more likely to experience cognitive decline, both at 1 and 12 months after surgery, and are less likely to return to their preoperative cognitive baseline.[6] Finally, even though many consider delirium to be a purely neuropsychiatric entity, patients who experience delirium are more likely to die, with reported adjusted relative risks ranging from 1.4 to 13.0.[1] In the United States alone, delirium in patients over the age of 65 is estimated to cost more than $164 billion per year.[7] Compared to the general population of hospitalized elderly, cardiac surgery patients are among those at greatest risk of delirium, a risk 8-fold higher than for patients undergoing non-cardiac surgery.[1]

1.2 Benzodiazepines and Delirium

Observational studies have identified multiple non-modifiable risk factors for delirium, including age, preoperative cognitive impairment, and hearing/vision problems. Modifiable risk factors include polypharmacy, use of psychoactive medications, and application of physical restraints. Among the modifiable precipitants of delirium, benzodiazepine administration is repeatedly identified. Both preoperative[8] and postoperative[2,9-12] benzodiazepine use is associated with delirium. In a nested case-control study in non-cardiac surgery patients, those who were administered benzodiazepines after surgery had an odds ratio for the development of delirium of 3.0 (95% CI 1.3 - 6.8) compared to those who did not.[2] A dose-response effect was observed in prospective observational data, suggesting that for every mg of midazolam administered after cardiac surgery, patients were 7-8% more likely to develop delirium.[11]

The pathophysiologic basis for the relationship between benzodiazepines and delirium has been speculatively attributed to gamma-aminobutyric acid (GABA), the primary inhibitory neurotransmitter in the central nervous system (CNS).[13] Imbalances in the release, synthesis, and degradation of GABA – as well as other neurotransmitters – are linked to the development of delirium.[13] Because of their high affinity for GABAergic receptors in brain regions responsible for consciousness, benzodiazepine-induced decreases in CNS arousal may cause unpredictable neurotransmission and resultant delirium.[13]

1.3 Assessment of Delirium after Cardiac Surgery

Delirium after cardiac surgery is such a serious problem that the incidence of delirium in the cardiovascular intensive care unit (ICU) is now often used as a quality metric.[14] The two validated delirium assessment scales most commonly used in the intensive care unit are the 'Confusion Assessment Method-ICU' (CAM-ICU) and the 'Intensive Care Delirium Screening Checklist' (ICDSC).[15] These scales allow accurate diagnosis of delirium and both can be administered in 2-5 minutes.[16,17] Multiple studies in ICU and non-ICU settings have shown that without validated screening tools, bedside nurses and physicians may fail to recognize when patients are experiencing delirium.[18-20] As such, the most recent Society of Critical Care Medicine guidelines recommend that critically ill adults be regularly assessed using a validated screening tool.[15] This recommendation has been incorporated into routine

nursing practice in the majority of hospitals providing care to critically ill and cardiac surgery patients in Canada and, in some centres, all patients after noncardiac surgery.[3,15]

1.4 Benzodiazepine Use during and after Cardiac Surgery

Benzodiazepines are used in two specific and different settings in cardiac surgery patients: (i) After cardiac surgery in the ICU for sedation and (ii) During cardiac surgery to induce amnesia and treat anxiety.

1.5 Benzodiazepines After Surgery

Several randomized controlled trials have compared benzodiazepines to the alpha-2 agonist dexmedetomidine for sedation after cardiac surgery in the ICU.[10,12,21] In a 106-patient randomized trial, sedation with lorazepam (a benzodiazepine) resulted in fewer days alive without delirium (median days 7.0 vs. 3.0; p = .01) than sedation with dexmedetomidine.[10] In another randomized trial of 366 patients, patients sedated with a benzodiazepine infusion had a relative risk of delirium of 1.41 (95% CI 1.20-1.64) when compared to a dexmedetomidine infusion.[12] Overall, the meta-analyzed results were likely underpowered, but showed a trend towards increased delirium with benzodiazepine sedation, with a relative risk of 1.23 (95% CI 0.93-1.67).[15] This evidence has led to recommendations, from the American Geriatric Society[3] and Society for Critical Care Medicine[22] that benzodiazepine use be minimized in elderly and ICU patients. As a result, except in special circumstances, benzodiazepine for sedation after cardiac surgery is no longer considered consistent with the standard of care in Canada.[3,22,23] This is not true for benzodiazepine use during cardiac surgery, where the practice remains common.[24]

1.6 Benzodiazepine during Cardiac Surgery

Although benzodiazepine use in the ICU has decreased, postoperative delirium remains a serious problem after cardiac surgery, affecting 15-20%.[25,26] This may be in part related to the fact that benzodiazepine use during cardiac surgery (where the indication is different from in the ICU) remains common. Benzodiazepine use remains common during cardiac surgery, because of the favourable hemodynamic and amnestic properties of these medications, as well as their anxiolytic effects. While one study has identified higher doses of intraoperative benzodiazepines as a risk factor for postoperative delirium,[27] no studies have evaluated the effect of a benzodiazepine-free anesthetic regime on delirium after cardiac surgery.

A key reason for ongoing benzodiazepine use in the OR is based on the concept that benzodiazepines decrease the risk of awareness during cardiac anesthesia.[28,29] The concern about awareness among cardiac surgery patients arises because of patient comorbidities and hemodynamic instability, which can make it difficult to avoid periods of light anesthesia, which in turn increases the risk of awareness.[30] In addition, the physiologic normalization of blood pressure and absence of heart rate associated with cardiopulmonary bypass may mask some of the changes that would normally alert anesthetists to the need for a greater depth of anesthesia.[30] However concern about awareness is a weak reason to use a benzodiazepine during surgery. Awareness of intraoperative events during surgery is actually rare, with an incidence of 0.1-0.2% in the general surgical population[31] and only 1.1%[32] to 1.5% in cardiac surgery patients.[33] Benzodiazepines appear to be less effective against awareness than many people believe as several large studies have reported awareness in patients who have received benzodiazepines.[31,34] Effective methods to monitor for intraoperative awareness (e.g. processed electroencephalographic monitoring) are now routinely used and reduce the risk of awareness. Alternative medications with similar hemodynamic profiles to benzodiazepines, but without

the effects on GABA (e.g. etomidate), are available. As such, the need to rely on benzodiazepines during cardiac surgery is minimal. Nonetheless, benzodiazepine use remains common due to the absence of evidence supporting a benzodiazepine limited approach.

Because there are alternatives to the use of benzodiazepines during cardiac surgery, there is considerable variation in the use of benzodiazepines during cardiac surgery in Canada. We conducted a survey (70% response rate) of cardiac anesthesia practice regarding benzodiazepine use in Canada and documented this variability in practice[24] (See Figure 1): During cardiac surgery 11% of respondents never gave benzodiazepines and 21% always gave benzodiazepines. When benzodiazepines were given to patients during cardiac surgery, midazolam was the drug most commonly used, with a mean (standard deviation) dose of 4.9 (3.8) mg given to an average patient. When respondents were asked the proportion of patients to whom they gave benzodiazepines during cardiac surgery, the response was bimodal (see Figure 1). The most common considerations that made respondents more likely to give benzodiazepines during cardiac surgery use were young patient age (73%), patient anxiety (63%), history of alcohol/drug/benzodiazepine use (60%), and the presence of risk factors for intraoperative awareness (44%).[24]

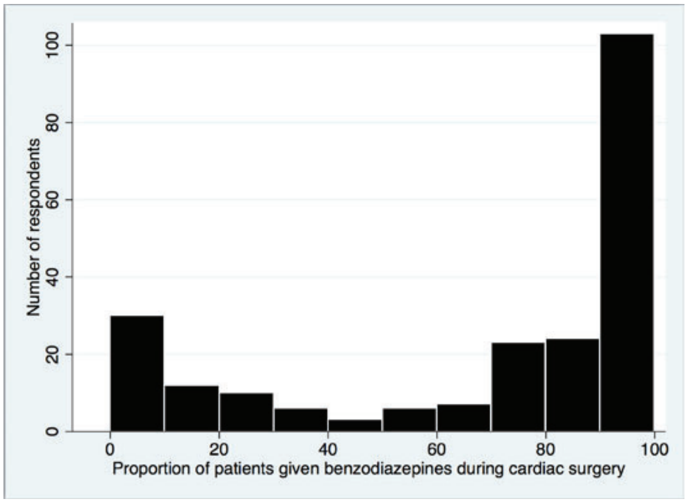

Figure 1: Proportion of patients given benzodiazepines during cardiac surgery by survey respondents

In summary, delirium continues to occur in 15-20% of patients in the ICU after cardiac surgery.[25,26] It is associated with significant morbidity and mortality[1,5,6] and may be due to ongoing use of benzodiazepines during surgery. However, good alternatives to benzodiazepines exist, and there is now uncertainty as to whether or not benzodiazepines should be used during surgery, as demonstrated by the large variation in clinical practice in Canada.[15] Whether benzodiazepines should be avoided during cardiac surgery in most patients is a question that urgently needs to be answered. This gives rise to our specific research question: Does a policy of limited intraoperative benzodiazepine use during adult cardiac surgery, compared to a policy of liberal intraoperative benzodiazepine use, reduce the incidence of post-operative delirium?

1.7 <u>Rationale for a Cluster trial</u>

Individual patient efficacy trials are useful to establish the clinical efficacy of an intervention amongst a carefully selected population under optimal conditions following detailed protocols. However, such trials do not address questions of clinical effectiveness, which are questions about how well an intervention or policy actually works in clinical practice.

Cardiac surgery is done in specialized institutions performing high volumes of surgery in order to reduce complications and increase efficiency. The surgical care of patients in these high-volume cardiac surgery centres is undertaken using standardized procedures that optimize outcomes, such as standard preoperative assessment and pre-and postoperative care pathways. Because cardiac surgical care is delivered through standard institutional policies, it is appropriate to address whether an institutional policy of limiting benzodiazepine use during surgery would reduce delirium. Testing the effects of different institutional policies mandates a pragmatic trial done with randomization of institutions rather than patients. Thus, this study will use a cluster design; specifically addressing whether an institutional policy of limited use of benzodiazepines during surgery (compared to liberal use) reduces post-operative delirium. The main challenge of a cluster-randomized trial is the substantial loss of statistical power that occurs as a result of clustering. This is because individuals being studied within a cluster are usually more similar than individuals across clusters, a phenomenon described statistically by the intra-cluster correlation coefficient (ICC).[35] This study will use multiple crossovers between intervention arms within each cluster to minimize the loss of statistical power, with a design known as the randomized cluster crossover trial. [36]

In the planned trial, each institution is randomized to one of the two policies of benzodiazepine use (limited or liberal) which they use as standard institutional policy for four weeks. At the end of each period, sites are re-randomized to a new standard policy that is used for the subsequent four weeks. Each site thus acts as its own control group, which mitigates the effect of imbalance between sites in patient and provider characteristics.[35] This design is methodologically rigorous and tests the effect of a change in standard policy, as it would actually be used in the clinical setting.

2. OBJECTIVES

2.1 Primary Objective

The primary objective of the B-Free trial is to evaluate the impact of an institutional policy of limited benzodiazepine use during cardiac surgery, as compared to a policy of liberal benzodiazepine use during cardiac surgery, on the incidence of delirium during the initial 72 hours after cardiac surgery.

2.2 Secondary Objectives

The secondary objectives of the B-Free trial are to evaluate the impact of these policies on:

4. ICU length of stay (LOS)
5. Hospital LOS
6. All cause in-hospital mortality

3. STUDY DESIGN

3.1 Type of Study

B-Free is a multicenter, cluster-randomized, crossover trial.

3.2 Expected Number of Clusters and Patients

The expected number of hospitals for the trial is 16, with an average annual case volume of 1,000, for a total of approximately 16,000 patients.

3.3 Method of Intervention Allocation

Each site will be randomized to one of the two policies to be used as per institutional policy by all anesthetists. The policies to be tested do not require that all patients be treated identically as they allow for reasonable exceptions. Sites will be randomized to one policy and then cross-over to the other policy at set times. Sites will be randomized to twelve, 4-week crossover periods, blocking in periods of 2 to minimize period effects (See Appendix 1: Sample Site Randomization Schedule). Randomization for all periods will take place in advance of site start-up, but clusters will only be notified of the subsequent period's standard policy during the last week of each crossover period.

3.4 Methods for Protecting Against Bias

As this is a pragmatic trial all patients treated during each period of study will be included in all analyses. Individuals collecting delirium data after cardiac surgery in the ICU will not be informed of the institutional policy of benzodiazepine currently in use in the operating room at the institution. By including a large number of clusters undergoing multiple crossovers and blocking in periods of two, we will minimize the bias that may occur because of cluster or period effects.

3.5 Duration of Each Intervention Period

The duration of each intervention period is approximately 4 weeks; each cluster will be randomized to 12 such 4-week periods.

3.6 Duration of Follow-up

Patients will be followed until their hospital discharge.

4. CRITERIA FOR INCLUSION OF A HOSPITAL

1. Major surgical center with a minimum of 500 cases of cardiac surgery per year
2. Equipoise by the hospital physicians regarding the use of benzodiazepines during surgery (≥ 95% of hospital cardiac anesthesia group agrees to manage patients as per the benzodiazepine policy in place during a given crossover period)
3. Patients are routinely assessed for postoperative delirium at least once every 12 hours during the initial 72 hours after cardiac surgery as a part of routine clinical care using either the Confusion Assessment Method-ICU (CAM-ICU) or the Intensive Care Delirium Screening Checklist (ICDSC)

5. BENZODIAZEPINE POLICIES TO BE STUDIED

Limited Benzodiazepine Policy*:*

1. No routine use of any intraoperative benzodiazepines.
2. Accepted benzodiazepine use in the case of seizure, alcohol withdrawal, severe anxiety, history of awareness during anesthesia, or known benzodiazepine dependence.
3. Accepted benzodiazepine use in patients who are hemodynamically unstable and/or have cardiac anatomy that puts them at high risk of developing ischemia on induction of anesthesia using other agents.

Liberal Benzodiazepine Policy:

1. Administration of benzodiazepine as per clinical guidelines but no lower than 0.03 mg/kg (ideal body weight midazolam equivalent) to all patients undergoing cardiac surgery. Any benzodiazepine may be used.
2. Accepted avoidance of benzodiazepine use in patients who have contraindications to the administration of these medications (i.e. documented allergy, previous adverse reaction to benzodiazepine).

5.1 Pre- and Post-operative Benzodiazepine Administration

Benzodiazepine administration before and after surgery will be minimized during both policy implementation periods in accordance with current guidelines. Recognized exceptions to this include patients who are benzodiazepine dependent, alcohol dependent, or having seizures.

5.2 Study Data Collection

Data will be collected from hospital administrative databases, chart reviews and/or electronic medical records. Data collected will include key baseline characteristics such as demographics, details of surgery, postoperative delirium, and pre- and postoperative medications. Encryption of patient identifiers will be used to ensure confidentiality. Investigative sites will transfer the data to PHRI for central data management. Data transfer specification and specific data points to be collected will be detailed in a separate Study Operations Manual.

6. STUDY OUTCOMES

6.1 Primary Outcome

The primary study outcome is the percentage of patients with delirium assessed during the initial 72 hours following cardiac surgery.

6.2 Secondary Outcomes

1. ICU LOS
2. Hospital LOS
3. In-hospital mortality

6.3 Outcome Definitions

1. Assessment of delirium: Delirium will be measured using either the Confusion Assessment Method-ICU (CAM-ICU) or the Intensive Care Delirium Screening Checklist (ICDSC).
2. ICU LOS: This is defined as the number of hours in the cardiac surgical ICU following index cardiac surgery until ICU discharge.
3. Hospital LOS: This is defined as the number of days from index cardiac surgery until hospital discharge.
4. In-hospital mortality: This is defined as death from any cause after the index cardiac surgical procedure and until hospital discharge.

7. STATISTICAL CONSIDERATIONS

7.1 Analysis Population

All adult (≥ 18 years) patients undergoing cardiac surgery at each included cluster will be included in the analysis according to the crossover period policy in place at the time they undergo surgery, regardless of how they are managed. Each cluster will apply each of the two policies six times during twelve, 4-week crossover periods.

7.2 Statistical Methods

The primary analyses will be based on the intention-to-treat principle, i.e. participants will be analyzed according to the policy in use when they underwent surgery, regardless of whether or not they were managed according to the policy. All analyses will take place at the individual level. Analyses will be carried out comparing event rates in patients managed during the benzodiazepine liberal and limited arms. The primary and secondary outcomes will be estimated between the treatment groups using a hierarchical mixed model for binary outcomes (i.e. GLIMMIX) adjusted for cluster and cluster-by-period as random effects terms (Turner et al., 2007). A sensitivity analysis will be conducted to evaluate the primary outcome in patients managed per protocol. The odds ratios and 95% confidence intervals will be reported. Statistical significance will be claimed if the p value is less than 0.05 for the primary outcome for treatment effectiveness. Carryover effects will be assessed for the primary outcomes using tests for interactions between periods and treatment effects using hierarchical mixed models. SAS 9.4 for UNIX (SAS Institute Inc., Cary, North Carolina, USA) or other validated software will be used for all analyses. A comprehensive plan for analysis will be detailed in a separate Statistical Analysis Plan (SAP).

7.3 Planned Subgroup Analyses

The following subgroups of interest will be evaluated: sex, age, benzodiazepine dose, patients who receive no preoperative benzodiazepines, and urgent/emergent surgery.

7.4 Interim Analysis

One interim analysis will be undertaken when half the sites have completed at least 6 periods. For efficacy, a modified Haybittle-Peto approach will be used to evaluate both the primary outcome (delirium incidence) and the secondary outcome of in-hospital mortality. An independent Data Monitoring Committee (DMC) will review the interim analysis data and make recommendations to the study leadership about the conduct of the trial, integrity of the data and trial discontinuation to ensure the overall safety of patients. The guiding policies and operating procedures governing the DMC will be described in a separate DMC charter.

7.5 Sample Size Calculation

In B-Free, the intracluster correlation coefficient (ICC) will depend on the incidence of delirium in each participating cluster and the interperiod correlation coefficient (IPC) will be assumed to be half of ICC. Given an estimated prevalence of delirium of 15% (derived from local administrative data and confirmed in our pilot study at two centres), we estimated a conservative ICC of 0.02, based on values determined by Gulliford et al. using several large administrative data sets. Based on the assumption of an ICC = 0.02, an IPC = 0.5*ICC, a relative risk reduction of 15%, an adherence rate for each policy of 80%, and an average total cluster size of 1,000, we will require a total sample of 15,886 patients studied within 16 participating hospitals each with 12 periods (see Table 1).

Table 1: Total patients (N) and number of hospitals (N/ m̃) required to be randomized for each of two intervention groups to assure a sufficient power of 80% and a Type I error rate of 5% (2-sided) with 12 periods, a coefficient of variation of 0.63 for an assumed unequal cluster size, an anticipated control event rate (p_1) of 15% for different combinations of average cluster size per cluster (m̃), intracluster correlation (ICC) and relative risk reduction (RRR).

RRR	Overall Cluster size ($\overline{m}$)	# periods	ICC	Total N (inflated)	Sample size for an individual patient RCT	# hospitals (N/$\overline{m}$)
15%	1000	12	0.01	11648	7409	12
		12	0.02	**15886**	**7409**	**16**
	800	12	0.01	10785	7409	14
		12	0.02	14161	7409	18
20%	1000	12	0.01	6402	4072	7
		12	0.02	8731	4072	9
	800	12	0.01	5928	4072	8
		12	0.02	7783	4072	10

8. DATA MANAGEMENT

Data management will be performed by the PHRI in accordance with PHRI standards and procedures for collection and validation of data. Data will be collected directly through the hospital electronic medical record (EMR) database and on electronic case report forms (eCRFs) using an electronic data capture system, based on the availability of data in the site's EMR. Data for each patient that is entered into the eCRF will be verified by the Investigator. It is the Investigator's responsibility to ensure the accuracy, completeness, legibility, and timeliness of the data reported on the patient's eCRF. All data will be kept secure and confidentiality of all study patients will be carefully protected. Data will be validated, managed, and stored in a de-identified database on a secure server at PHRI.

9. STUDY ORGANIZATION

The Population Health Research Institute (PHRI), a joint institute of McMaster University and Hamilton Health Sciences, is the study Sponsor and will be the study coordination centre responsible for the trial organization, coordination of the international centres, as well as data management and analysis.

The Steering Committee will be responsible for the design, execution, analysis, and reporting of the study. This committee will convene regularly by telephone conference or meetings to address issues and to monitor study progress, execution, and management. The Steering Committee will include the Principal Investigators, several key investigators, and two patient partners. The Steering Committee will hold the primary responsibility for publication of the study results on behalf of the B-Free investigators.

10. ETHICAL STANDARDS

10.1 Ethical Considerations

The study will be conducted in accordance with Good Clinical Practice (GCP), all applicable subject privacy requirements, and the guiding principles of the Declaration of Helsinki, including, but not limited to:

- Institutional Review Board (IRB)/Research Ethics Board (REB) review and approval of study protocol and any subsequent amendments.
- A modified consent process approved by the IRB.

10.2 Waiver of Individual Patient Consent

The cluster design challenges conventional approaches to clinical research because patients cannot choose to avoid the intervention, nor consent for the study because the intervention is applied at the level of the health care environment and not the patient. B-Free evaluates two different cardiac anesthesia policies related to the use of benzodiazepines (minimized versus liberal intraoperative administration). Centres will only be included if there is physician equipoise regarding the benzodiazepine policies being tested in this study. The different approaches to the use of benzodiazepine, embodied in the two study policies being evaluated, are both of minimal risk and are commonly used in Canada and other countries[10]. We will obtain waiver of individual patient consent at each participating site before study initiation, according to criteria proposed by the Tri-Council Policy Statement (TCPS 2): Ethical Conduct for Research Involving Humans[37,38]. This study fulfills TCPS 2 criteria in that: (i) the study poses minimal risk to patients; (ii) waiver of consent will not adversely affect patient rights and welfare; (iii) it would be impracticable to carry out the research if prior consent is required; and (iv) patients and/or families will be provided with information about the study using an information letter and/or poster in ICU waiting areas.

10.3 Ethics Review and Approval

Local investigators must submit this protocol to an Ethics Review Committee or a similar body (IRB, REB) and is required to forward a copy of the written approval/advice signed by the Chair to PHRI. On the approval/advice sheet, the trial name and protocol version, the study documents (protocol and letter of information), and the date of the review should be clearly stated.

11. INVESTIGATOR AND ADMINISTRATIVE REQUIREMENTS

11.1 Investigator Responsibilities

The Investigator will allow representatives of PHRI to periodically conduct relevant audits of the investigative site. The monitoring visits provide PHRI with the opportunity to evaluate the progress of the study; ensure that all protocol requirements, applicable regulations, and Investigator's obligations are being fulfilled; and resolve any issues that may arise.

The Investigator(s) undertake(s) to conduct the study in accordance with Good Clinical Practice. The Investigator is required to ensure adherence with respect to implementation of the two policies according to the randomization schedule and procedures required by the protocol.

11.2 Confidentiality and Disclosure of Data

All goods, materials, information (oral or written) and unpublished documentation provided to the Investigators, inclusive of this protocol, are the exclusive property of the Sponsor. They may not be given or disclosed by the Investigator or by any person within their authority either in part or in totality to any unauthorized person without the prior written formal consent of the Sponsor. It is specified that

the submission of this protocol and other necessary documentation to the IRB or a like body is expressly permitted, the IRB members having the same obligation of confidentiality.

The Investigator shall consider as confidential and shall take all necessary measures to ensure that there is no breach of confidentiality in respect of all information accumulated, acquired or deduced in the course of the trial, other than that information to be disclosed by law.

Any personal health information obtained as a result of this study is considered confidential and disclosure to third parties other than those noted below is prohibited. The study personnel, employees of the regulatory agencies, including Health Canada and the study sponsor, PHRI, and its agents may need to review patient medical records in order to accurately record information for this study. If results of this study are reported in medical journals or at meetings, the patient's identity will remain confidential.

11.3 Record Retention

The Investigator must retain trial records for the amount of time specified by applicable laws and regulations or by ICH E6 Good Clinical Practice guidelines, whichever is longer. In Canada, retention of all records created during the conduct of a clinical trial is 25 years. All trial documents shall be made available upon request from relevant health authorities. Any investigative center will consult the PHRI Coordinating Centre before discarding trial and/or subject files.

12. OWNERSHIP OF DATA AND USE OF STUDY RESULTS

The Sponsor and B-Free Steering Committee of the study have the ownership of all data and results collected during this study. In consequence, the Sponsor reserves the right to use the data of the present study, with or without comments and with or without analysis, in order to submit them to the Health Authorities of any country. Full publication rights of the study data solely reside with the Sponsor and Steering Committee.

13. PUBLICATION POLICY

All study presentations and/or publication of the results will be based on clean, checked and validated data in order to ensure the accuracy of the results. All analyses for publication will be provided by the PHRI Coordinating Centre. The responsibility for presentations and/or publications belongs to the Steering Committee. The final content of the manuscript is the responsibility of the Steering Committee. Publication of the main findings of this study will be made jointly in the name of all collaborators. Other papers will be authored based on the contributions of the individuals to the overall study. All the trial participants (Investigators and committee members) make a prior delegation of responsibility for primary presentation and/or primary publication of the results to the Steering Committee. No other publication is allowed before the primary publication. Any presentation or publication by any trialist must mention the trial and has to be approved by the Steering Committee. Moreover, it is mandatory to make reference to the primary publication.

REFERENCES

1. Inouye SK, Westendorp RG, Saczynski JS. Delirium in elderly people. Lancet 2014;383:911-22.
2. Marcantonio ER, Juarez G, Goldman L, et al. The relationship of postoperative delirium with psychoactive medications. JAMA 1994;272:1518-22.
3. American Geriatrics Society Expert Panel on Postoperative Delirium in Older Adults. Postoperative delirium in older adults: best practice statement from the American Geriatrics Society. J Am Coll Surg 2015;220:136-48 e1.
4. Dasgupta M, Dumbrell AC. Preoperative risk assessment for delirium after noncardiac surgery: a systematic review. J Am Geriatr Soc 2006;54:1578-89.
5. Gleason LJ, Schmitt EM, Kosar CM, et al. Effect of Delirium and Other Major Complications on Outcomes After Elective Surgery in Older Adults. JAMA surg 2015:1-7.
6. Saczynski JS, Marcantonio ER, Quach L, et al. Cognitive trajectories after postoperative delirium. N Engl J Med 2012;367:30-9.
7. Leslie DL, Marcantonio ER, Zhang Y, Leo-Summers L, Inouye SK. One-year health care costs associated with delirium in the elderly population. Arch Intern Med 2008;168:27-32.
8. Redelmeier DA, Thiruchelvam D, Daneman N. Delirium after elective surgery among elderly patients taking statins. Can Med Assoc J 2008;179:645-52.
9. Pandharipande P, Shintani A, Peterson J, et al. Lorazepam is an independent risk factor for transitioning to delirium in intensive care unit patients. Anesthesiology 2006;104:21-6.
10. Pandharipande PP, Pun BT, Herr DL, et al. Effect of sedation with dexmedetomidine vs lorazepam on acute brain dysfunction in mechanically ventilated patients: the MENDS randomized controlled trial. JAMA 2007;298:2644-53.
11. Taipale PG, Ratner PA, Galdas PM, et al. The association between nurse-administered midazolam following cardiac surgery and incident delirium: an observational study. Int J Nurs Stud 2012;49:1064-73.
12. Riker RR, Shehabi Y, Bokesch PM, et al. Dexmedetomidine vs midazolam for sedation of critically ill patients: a randomized trial. JAMA 2009;301:489-99.
13. Gunther ML, Morandi A, Ely EW. Pathophysiology of delirium in the intensive care unit. Crit Care Clin 2008;24:45-65, viii.
14. Berian JR, Zhou L, Russell MM, et al. Postoperative Delirium as a Target for Surgical Quality Improvement. Ann Surg 2018;268:93-9.
15. Devlin JW, Skrobik Y, Gelinas C, et al. Clinical Practice Guidelines for the Prevention and Management of Pain, Agitation/Sedation, Delirium, Immobility, and Sleep Disruption in Adult Patients in the ICU. Crit Care Med 2018;46:e825-e73.
16. Andrews L, Silva SG, Kaplan S, Zimbro K. Delirium monitoring and patient outcomes in a general intensive care unit. Am J Crit Care 2015;24:48-56.
17. van den Boogaard M, Pickkers P, van der Hoeven H, Roodbol G, van Achterberg T, Schoonhoven L. Implementation of a delirium assessment tool in the ICU can influence haloperidol use. Crit Care 2009;13:R131.
18. Bigatello LM, Amirfarzan H, Haghighi AK, et al. Effects of routine monitoring of delirium in a surgical/trauma intensive care unit. J Trauma Acute Care Surg 2013;74:876-83.
19. Devlin JW, Fong JJ, Schumaker G, O'Connor H, Ruthazer R, Garpestad E. Use of a validated delirium assessment tool improves the ability of physicians to identify delirium in medical intensive care unit patients. Crit Care Med 2007;35:2721-4; quiz 5.

20. Spronk PE, Riekerk B, Hofhuis J, Rommes JH. Occurrence of delirium is severely underestimated in the ICU during daily care. Intens Care Med 2009;35:1276-80.
21. Maldonado JR, Wysong A, van der Starre PJ, Block T, Miller C, Reitz BA. Dexmedetomidine and the reduction of postoperative delirium after cardiac surgery. Psychosom 2009;50:206-17.
22. Barr J, Fraser GL, Puntillo K, et al. Clinical practice guidelines for the management of pain, agitation, and delirium in adult patients in the intensive care unit. Crit Care Med 2013;41:263-306.
23. Ferrell BA, Girard TD. Sedative choice: a critical decision. Am J Respir Crit Care Med 2014;189:1295-7.
24. Spence J, Belley-Cote E, Devereaux PJ, et al. Benzodiazepine administration during adult cardiac surgery: a survey of current practice among Canadian anesthesiologists working in academic centres. Can J Anesth 2018;65:263-71.
25. Arenson BG, MacDonald LA, Grocott HP, Hiebert BM, Arora RC. Effect of intensive care unit environment on in-hospital delirium after cardiac surgery. J Thorac Cardiovasc Surg 2013;146:172-8.
26. Pauley E, Lishmanov A, Schumann S, Gala GJ, van Diepen S, Katz JN. Delirium is a robust predictor of morbidity and mortality among critically ill patients treated in the cardiac intensive care unit. Am Heart J 2015;170:79-86, e1.
27. Kazmierski J, Kowman M, Banach M, et al. Incidence and predictors of delirium after cardiac surgery: Results from The IPDACS Study. J Psychosom Res 2010;69:179-85.
28. American Society of Anesthesiologists Task Force on Intraoperative Awareness. Practice advisory for intraoperative awareness and brain function monitoring: a report by the american society of anesthesiologists task force on intraoperative awareness. Anesthesiology 2006;104:847-64.
29. Myles PS, Symons JA, Leslie K. Anaesthetists' attitudes towards awareness and depth-of-anaesthesia monitoring. Anaesthesia 2003;58:11-6.
30. Serfontein L. Awareness in cardiac anesthesia. Curr Opin Anesthesiol 2010;23:103-8.
31. Sandin RH, Enlund G, Samuelsson P, Lennmarken C. Awareness during anaesthesia: a prospective case study. Lancet 2000;355:707-11.
32. Phillips AA, McLean RF, Devitt JH, Harrington EM. Recall of intraoperative events after general anaesthesia and cardiopulmonary bypass. Can J Anesth 1993;40:922-6.
33. Ranta S, Jussila J, Hynynen M. Recall of awareness during cardiac anaesthesia: influence of feedback information to the anaesthesiologist. Acta anaesthesiol Scand 1996;40:554-60.
34. Myles PS, Leslie K, McNeil J, Forbes A, Chan MT. Bispectral index monitoring to prevent awareness during anaesthesia: the B-Aware randomised controlled trial. Lancet 2004;363:1757-63.
35. Arnup SJ, Forbes AB, Kahan BC, Morgan KE, McKenzie JE. Appropriate statistical methods were infrequently used in cluster-randomized crossover trials. J Clin Epidemiol 2016;74:40-50.
36. Connolly SJ, Philippon F, Longtin Y, et al. Randomized cluster crossover trials for reliable, efficient, comparative effectiveness testing: design of the Prevention of Arrhythmia Device Infection Trial (PADIT). Can J Cardiol 2013;29:652-8.
37. Canadian Institutes of Health Research, Natural Sciences and Engineering Research Council of Canada, and Social Sciences and Humanities Research Council of Canada. Tri-Council Policy Statement: Ethical Conduct for Research Involving Humans, December 2014. Available from: http://www.pre.ethics.gc.ca/eng/policy-politique/initiatives/tcps2-eptc2/Default/. Accessed May 23, 2018.
38. United States Food and Drug Administration (USFDA). IRB Waiver or Alteration of Informed Consent for Clinical Investigations Involving No More Than Minimal Risk to Human Subjects. Available

from: https://www.fda.gov/downloads/RegulatoryInformation/Guidances/UCM566948.pdf. Accessed May 23, 2018.

APPENDIX I. Sample Site Randomization Schedule

Site 1	Ad Lib	B-Free	Ad Lib	B-Free	Ad lib	B-Free	Ad lib	B-Free	Ad lib	B-Free	Ad lib	B-Free
Site 2	B-Free	Ad Lib	B-Free	Ad Lib	B-Free	Ad Lib	B-Free	B-Free	Ad Lib	Ad Lib	B-Free	Ad Lib
Site 3	B-Free	Ad Lib	B-Free	Ad Lib	B-Free	Ad Lib	B-Free	Ad Lib	B-Free	Ad Lib	B-Free	Ad Lib
Site 4	Ad Lib	B-Free	Ad Lib	B-Free	Ad Lib	Ad Lib	B-Free	B-Free	Ad Lib	Ad Lib	B-Free	B-Free
Site 5	B-Free	Ad Lib	B-Free	Ad Lib	B-Free	Ad Lib	B-Free	Ad Lib	B-Free	B-Free	Ad Lib	Ad Lib
Site 6	Ad Lib	B-Free	Ad Lib	B-Free	B-Free	Ad Lib	B-Free	Ad Lib	Ad Lib	B-Free	B-Free	Ad Lib
Site 7	Ad Lib	B-Free	Ad Lib	B-Free	Ad Lib	Ad Lib	B-Free	B-Free	Ad Lib	B-Free	B-Free	Ad Lib
Site 8	B-Free	Ad Lib	B-Free	Ad Lib	Ad Lib	B-Free	B-Free	Ad Lib	B-Free	B-Free	Ad Lib	Ad Lib
Site 9	Ad Lib	B-Free	Ad Lib	B-Free	Ad Lib	B-Free	Ad Lib	B-Free	Ad Lib	B-Free	Ad Lib	B-Free
Site 10	B-Free	Ad Lib	B-Free	Ad Lib	B-Free	Ad Lib	Ad Lib	B-Free	Ad Lib	B-Free	Ad Lib	B-Free
Site 11	B-Free	Ad Lib	B-Free	Ad Lib	Ad Lib	B-Free	B-Free	Ad Lib	Ad Lib	B-Free	Ad Lib	B-Free
Site 12	B-Free	Ad Lib	B-Free	B-Free	Ad Lib	Ad Lib	B-Free	Ad Lib	Ad Lib	B-Free	B-Free	Ad Lib
Site 13	Ad Lib	B-Free	Ad Lib	B-Free	Ad Lib	B-Free	Ad Lib	B-Free	B-Free	Ad Lib	Ad Lib	B-Free
Site 14	Ad Lib	B-Free	Ad Lib	B-Free	B-Free	Ad Lib	B-Free	Ad Lib	Ad Lib	B-Free	B-Free	Ad Lib
Site 15	B-Free	Ad Lib	B-Free	Ad Lib	B-Free	B-Free	Ad Lib	Ad Lib	B-Free	B-Free	Ad Lib	Ad Lib
Site 16	Ad Lib	B-Free	Ad Lib	B-Free	Ad Lib	Ad Lib	B-Free	B-Free	Ad Lib	B-Free	Ad Lib	B-Free
	Period 1	Period 2	Period 3	Period 4	Period 5	Period 6	Period 7	Period 8	Period 9	Period 10	Period 11	Period 12
	Run - in Period											

Clinical Trial Protocol

Benzodiazepine-free Cardiac Anesthesia for Reduction of Postoperative Delirium (B-Free)

Final Version 5.0
2023-02-13

This protocol is the confidential intellectual property of the Sponsor and B-Free Steering Committee.
Acceptance implies an agreement not to disclose information contained herein that is not otherwise publicly available, with the exception that it may be disclosed to a Research Ethics Board (REB) for the purpose of obtaining approval to conduct the study.
The REB is requested and expected to maintain confidentiality.
This document may not be used or published without the consent of consent of the Sponsor or Steering Committee.

INVESTIGATOR'S AGREEMENT

I, Jessica Spence ___________________________, the investigator, have examined this protocol:

B-Free

and I have fully discussed the objectives of this trial and the contents of this amended protocol with the B-Free Coordinating Center representative(s) from the Population Health Research Institute.

I agree to conduct the study according to this protocol and to comply with its requirements, subject to ethical and safety considerations.

I agree to comply with the International Council for Harmonisation Tripartite Guideline on Good Clinical Practice (GCP) and applicable regulations/guidelines and all locally applicable laws.

I agree to ensure that the confidential information contained in this document will not be used for any purpose other than the evaluation or conduct of the clinical investigation without the prior written consent of the sponsor.

Investigator Name: Jessica Spence

Investigator Signature: ____________________ **Date:** February 13, 2023

PROTOCOL APPROVAL

Signature:	Date:
	February 13, 2023

Jessica Spence, MD FRCPC
McMaster University, Hamilton Health Sciences
Hamilton, ON Canada

TRIAL LEADERSHIP AND MANAGEMENT

**Coordinating Center**	Population Health Research Institute (PHRI)<br>A Joint Institute of Hamilton Health Sciences and McMaster University<br><br>David Braley Cardiac, Vascular and Stroke Research Institute<br>Hamilton General Hospital<br>237 Barton Street East<br>Hamilton, ON L8L 2X2; Canada<br><br>B-FREE@phri.ca
**Principal Investigator**	**Jessica Spence MD FRCPC**<br>McMaster University, Hamilton Health Sciences<br>Hamilton, ON Canada
**Co-Principal Investigator**	**Eric Jacobsohn MBChB MPHE FRCPC**<br>University of Manitoba,<br>Winnipeg, MB Canada
**Steering Committee Chair**	**Stuart Connolly MD FRCPC**<br>McMaster University, Hamilton Health Sciences<br>Population Health Research Institute (PHRI)<br>Hamilton, ON Canada
**Study Sponsor**	Hamilton Health Sciences, through the Population Health Research Institute<br>237 Barton Street East<br>Hamilton, ON L8L 2X2; Canada

TABLE OF CONTENTS

ABBREVIATIONS 7
STUDY SYNOPSIS 8
1. Introduction and Rationale 10
2. Objectives 13
2.1 Primary Objective 13
2.2 Secondary Objectives 13
3. Study Design 13
3.1 Type of Study 13
3.2 Expected Number of Clusters and Patients 14
3.3 Method of Intervention Allocation 14
3.4 Methods for Protecting Against Bias 14
3.5 Duration of Each Intervention Period 14
3.6 Duration of Follow-up 14
4. Criteria for Inclusion of a Hospital 14
5. Benzodiazepine policies to be studied 15
6. Study Outcomes 15
6.1 Primary Outcome 15
6.2 Secondary Outcomes 15
6.3 Outcome Definitions 16
7. Statistical Considerations 16
7.1 Analysis Population 16
7.2 Statistical Methods 16
7.3 Planned Subgroup Analyses 16
7.4 Original Sample Size Calculation 17
7.5 Interim analysis 17
7.6 Adaptation to original design based on results of interim analysis 17
8. Data Management 18
9. Study Organization 18
10. Ethical Standards 19
10.1 Ethical Considerations 19
10.2 Waiver of Individual Patient Consent 19
10.3 Ethics Review and Approval 19

11. Investigator and Administrative Requirements 19
11.1 Investigator Responsibilities 19
12. Ownership of Data and Use of Study Results 20
13. Publication Policy 20
REFERENCES 22

ABBREVIATIONS

CAM-ICU	Confusion Assessment Method-ICU
CI	Confidence Interval
CNS	Central Nervous System
CPB	Cardiopulmonary Bypass
CVICU	Cardiovascular Intensive Care Unit
DSMB	Data Safety Monitoring Board
eCRF	Electronic Case Report Form
EMR	Electronic Medical Record
FDA	United States Food and Drug Administration
GABA	Gamma-aminobutyric Acid
GCP	Good Clinical Practice
ICC	Intra-cluster Correlation Coefficient
ICDSC	Intensive Care Delirium Screening Checklist
ICH	International Council for Harmonisation
ICU	Intensive Care Unit
IEC	Independent Ethics Committee
IPC	Interperiod Correlation Coefficient
IRB	Institutional Review Board
LOS	Length of Stay
PHRI	Population Health Research Institute
PRE	Government of Canada Panel on Research Ethics
REB	Research Ethics Board
SCCM	Society for Critical Care Medicine
TCPS	Tri-Council Policy Statement

STUDY SYNOPSIS

**Title**	Benzodiazepine-free cardiac anesthesia for the reduction of postoperative delirium (B-Free)
**Study Objectives**	The primary objective of the B-Free trial is to evaluate the impact of an institutional policy of limited benzodiazepine use during cardiac surgery, as compared to a policy of liberal benzodiazepine use during cardiac surgery, on the incidence of delirium up to 72 hours after surgery.<br><br>The secondary objectives are to evaluate the impact of these policies on:<br>1. ICU length of stay (LOS)<br>2. Hospital LOS<br>3. All cause in-hospital mortality
**Study Design**	A multi-centre, randomized cluster crossover trial.
**Hospital Eligibility:**	1. Tertiary cardiac surgical center<br>2. Equipoise by the hospital physicians regarding the use of benzodiazepines during surgery (≥ 95% of hospital cardiac anesthesia group agrees to manage patients as per the benzodiazepine policy in place during a given crossover period)<br>3. Patients are routinely assessed for postoperative delirium at least once every 12 hours after ICU admission as a part of routine clinical care using either the Confusion Assessment Method-ICU (CAM-ICU) or the Intensive Care Delirium Screening Checklist (ICDSC).
**Original sample size**	16 hospitals, with an average annual case volume of 1000 patients
**Observed sample size at time of interim analysis**	20 hospitals, with an average case volume of 750 patients
**Final sample size based on trial adaptation**	20 hospitals, with an average annual case volume of 900 patients
**Expected number of subjects**	Approximately 18,000 adult patients undergoing cardiac surgery.
**Study Intervention**	The 'Limited Benzodiazepine Policy' consists of the following:<br>1. No routine use of any intraoperative benzodiazepines.<br>2. Accepted benzodiazepine use in the case of seizure, alcohol withdrawal, severe anxiety, history of awareness during anesthesia, or known benzodiazepine dependence.<br>3. Accepted benzodiazepine use in patients who are hemodynamically unstable and/or have cardiac anatomy that puts

	them at high risk of developing ischemia on induction of anesthesia using other agents.
**Study Comparator**	The 'Liberal Benzodiazepine Policy' consists of the following:<br>1. Administration of benzodiazepine as per clinical guidelines but no lower than 0.03 mg/kg (ideal body weight midazolam equivalent) to all patients undergoing cardiac surgery. Any benzodiazepine may be used.<br>2. Accepted avoidance of benzodiazepine use in patients who have contraindications to the administration of these medications (i.e. documented allergy, previous adverse reaction to benzodiazepine).
**Primary Outcome**	The primary study outcome is the proportion of patients with delirium up to 72 hours after cardiac surgery.
**Statistical Analysis**	The primary analysis will be based on a modified intention to treat principle, all patients (aged ≥18 years) undergoing cardiac surgery who are assessed for delirium will be included in the analysis treated during a period regardless of whether or not they were managed according to the policy in place during the period. Analyses will be carried out comparing event rates in patients managed during the benzodiazepine liberal and limited arms). The primary and secondary outcomes will be estimated between the treatment groups using a hierarchical mixed model for binary outcomes (i.e. GLIMMIX) adjusted for cluster and cluster-by-period as random effects terms (Turner et al., 2007). A sensitivity analysis will be conducted to evaluate the primary outcome in patients managed per protocol. The odds ratios and 95% confidence intervals will be reported. Statistical significance will be claimed if the p value is less than 0.05 for the primary outcome for treatment effectiveness.<br>Carryover effects will be assessed for the primary outcomes using tests for interactions between periods and treatment effects using hierarchical mixed models.
**Duration of Study Period (per cluster)**	All included hospitals (clusters) will participate in the trial for a minimum of twelve, four-week crossover periods. 11/20 hospitals will participate for an additional 5 or 6, four-week crossover periods.

1. INTRODUCTION AND RATIONALE

1.1 Delirium is a Serious Complication of Cardiac Surgery

Delirium is an acute disorder of cognition and attention that has been likened to 'acute brain failure.'[1] It is the most common surgical complication in older adults,[2,3] affecting up to 50% of post-operative patients aged 65 years and older.[1,4] Delirium has significant consequences for patients, families, and communities, both in the short- and long-term. Patients who experience delirium after surgery have longer hospital stays, and are more likely to be readmitted within 30-days or discharged to an institution.[5] Those who get post-operative delirium are also significantly more likely to experience cognitive decline, both at 1 and 12 months after surgery, and are less likely to return to their preoperative cognitive baseline.[6] Finally, even though many consider delirium to be a purely neuropsychiatric entity, patients who experience delirium are more likely to die, with reported adjusted relative risks ranging from 1.4 to 13.0.[1] In the United States alone, delirium in patients over the age of 65 is estimated to cost more than $164 billion per year.[7] Compared to the general population of hospitalized elderly, cardiac surgery patients are among those at greatest risk of delirium, a risk 8-fold higher than for patients undergoing non-cardiac surgery.[1]

1.2 Benzodiazepines and Delirium

Observational studies have identified multiple non-modifiable risk factors for delirium, including age, preoperative cognitive impairment, and hearing/vision problems. Modifiable risk factors include polypharmacy, use of psychoactive medications, and application of physical restraints. Among the modifiable precipitants of delirium, benzodiazepine administration is repeatedly identified. Both preoperative[8] and postoperative[2,9-12] benzodiazepine use is associated with delirium. In a nested case-control study in non-cardiac surgery patients, those who were administered benzodiazepines after surgery had an odds ratio for the development of delirium of 3.0 (95% CI 1.3 - 6.8) compared to those who did not.[2] A dose-response effect was observed in prospective observational data, suggesting that for every mg of midazolam administered after cardiac surgery, patients were 7-8% more likely to develop delirium.[11]

The pathophysiologic basis for the relationship between benzodiazepines and delirium has been speculatively attributed to gamma-aminobutyric acid (GABA), the primary inhibitory neurotransmitter in the central nervous system (CNS).[13] Imbalances in the release, synthesis, and degradation of GABA – as well as other neurotransmitters – are linked to the development of delirium.[13] Because of their high affinity for GABAergic receptors in brain regions responsible for consciousness, benzodiazepine-induced decreases in CNS arousal may cause unpredictable neurotransmission and resultant delirium.[13]

1.3 Assessment of Delirium after Cardiac Surgery

Delirium after cardiac surgery is such a serious problem that the incidence of delirium in the cardiovascular intensive care unit (ICU) is now often used as a quality metric.[14] The two validated delirium assessment scales most commonly used in the intensive care unit are the 'Confusion Assessment Method-ICU' (CAM-ICU) and the 'Intensive Care Delirium Screening Checklist' (ICDSC).[15] These scales allow accurate diagnosis of delirium and both can be administered in 2-5 minutes.[16,17] Multiple studies in ICU and non-ICU settings have shown that without validated screening tools, bedside nurses and physicians may fail to recognize when patients are experiencing delirium.[18-20] As such, the most recent Society of Critical Care Medicine guidelines recommend that critically ill adults be regularly assessed using a validated screening tool.[15] This recommendation has been incorporated into routine

nursing practice in the majority of hospitals providing care to critically ill and cardiac surgery patients in Canada and, in some centres, all patients after noncardiac surgery.[3,15]

1.4 Benzodiazepine Use During and After Cardiac Surgery

Benzodiazepines are used in two specific and different settings in cardiac surgery patients: (i) After cardiac surgery in the ICU for sedation and (ii) During cardiac surgery to induce amnesia and treat anxiety.

1.5 Benzodiazepines After Surgery

Several randomized controlled trials have compared benzodiazepines to the alpha-2 agonist dexmedetomidine for sedation after cardiac surgery in the ICU.[10,12,21] In a 106-patient randomized trial, sedation with lorazepam (a benzodiazepine) resulted in fewer days alive without delirium (median days 7.0 vs. 3.0; p = .01) than sedation with dexmedetomidine.[10] In another randomized trial of 366 patients, patients sedated with a benzodiazepine infusion had a relative risk of delirium of 1.41 (95% CI 1.20-1.64) when compared to a dexmedetomidine infusion.[12] Overall, the meta-analyzed results were likely underpowered, but showed a trend towards increased delirium with benzodiazepine sedation, with a relative risk of 1.23 (95% CI 0.93-1.67).[15] This evidence has led to recommendations, from the American Geriatric Society[3] and Society for Critical Care Medicine[22] that benzodiazepine use be minimized in elderly and ICU patients. As a result, except in special circumstances, benzodiazepine for sedation after cardiac surgery is no longer considered consistent with the standard of care in Canada.[3,22,23] This is not true for benzodiazepine use during cardiac surgery, where the practice remains common.[24]

1.6 Benzodiazepine during Cardiac Surgery

Although benzodiazepine use in the ICU has decreased, postoperative delirium remains a serious problem after cardiac surgery, affecting 15-20%.[25,26] This may be in part related to the fact that benzodiazepine use during cardiac surgery (where the indication is different from in the ICU) remains common. Benzodiazepine use remains common during cardiac surgery, because of the favourable hemodynamic and amnestic properties of these medications, as well as their anxiolytic effects. While one study has identified higher doses of intraoperative benzodiazepines as a risk factor for postoperative delirium,[27] no studies have evaluated the effect of a benzodiazepine-free anesthetic regime on delirium after cardiac surgery.

A key reason for ongoing benzodiazepine use in the OR is based on the concept that benzodiazepines decrease the risk of awareness during cardiac anesthesia.[28,29] The concern about awareness among cardiac surgery patients arises because of patient comorbidities and hemodynamic instability, which can make it difficult to avoid periods of light anesthesia, which in turn increases the risk of awareness.[30] In addition, the physiologic normalization of blood pressure and absence of heart rate associated with cardiopulmonary bypass may mask some of the changes that would normally alert anesthetists to the need for a greater depth of anesthesia.[30] However, concern about awareness is a weak reason to use a benzodiazepine during surgery. Awareness of intraoperative events during surgery is actually rare, with an incidence of 0.1-0.2% in the general surgical population[31] and only 1.1%[32] to 1.5% in cardiac surgery patients.[33] Benzodiazepines appear to be less effective against awareness than many people believe as several large studies have reported awareness in patients who have received benzodiazepines.[31,34] Effective methods to monitor for intraoperative awareness (e.g. processed electroencephalographic monitoring) are now routinely used and reduce the risk of awareness. Alternative medications with similar hemodynamic profiles to benzodiazepines, but without

the effects on GABA (e.g. etomidate), are available. As such, the need to rely on benzodiazepines during cardiac surgery is minimal. Nonetheless, benzodiazepine use remains common due to the absence of evidence supporting a benzodiazepine limited approach.

Because there are alternatives to the use of benzodiazepines during cardiac surgery, there is considerable variation in the use of benzodiazepines during cardiac surgery in Canada. We conducted a survey (70% response rate) of cardiac anesthesia practice regarding benzodiazepine use in Canada and documented this variability in practice[24] (See Figure 1): During cardiac surgery 11% of respondents never gave benzodiazepines and 21% always gave benzodiazepines. When benzodiazepines were given to patients during cardiac surgery, midazolam was the drug most commonly used, with a mean (standard deviation) dose of 4.9 (3.8) mg given to an average patient. When respondents were asked the proportion of patients to whom they gave benzodiazepines during cardiac surgery, the response was bimodal (see Figure 1). The most common considerations that made respondents more likely to give benzodiazepines during cardiac surgery use were young patient age (73%), patient anxiety (63%), history of alcohol/drug/benzodiazepine use (60%), and the presence of risk factors for intraoperative awareness (44%).[24]

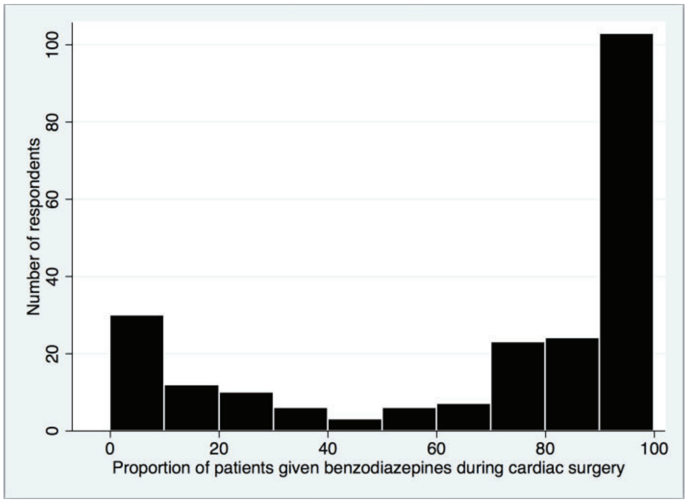

Figure 1: Proportion of patients given benzodiazepines during cardiac surgery by survey respondents

In summary, delirium continues to occur in 15-20% of patients in the ICU after cardiac surgery.[25,26] It is associated with significant morbidity and mortality[1,5,6] and may be due to ongoing use of benzodiazepines during surgery. However, good alternatives to benzodiazepines exist, and there is now uncertainty as to whether or not benzodiazepines should be used during surgery, as demonstrated by the large variation in clinical practice in Canada.[15] Whether benzodiazepines should be avoided during cardiac surgery in most patients is a question that urgently needs to be answered. This gives rise to our specific research question: Does a policy of limited intraoperative benzodiazepine use during adult cardiac surgery, compared to a policy of liberal intraoperative benzodiazepine use, reduce the incidence of post-operative delirium?

1.7 Rationale for a Cluster Trial

Individual patient efficacy trials are useful to establish the clinical efficacy of an intervention amongst a carefully selected population under optimal conditions following detailed protocols. However, such

trials do not address questions of clinical effectiveness, which are questions about how well an intervention or policy actually works in clinical practice.

Cardiac surgery is done in specialized institutions performing high volumes of surgery to reduce complications and increase efficiency. The surgical care of patients in these high-volume cardiac surgery centres is undertaken using standardized procedures that optimize outcomes, such as standard preoperative assessment and pre-and postoperative care pathways. Because cardiac surgical care is delivered through standard institutional policies, it is appropriate to address whether an institutional policy of limiting benzodiazepine use during surgery would reduce delirium. Testing the effects of different institutional policies mandates a pragmatic trial done with randomization of institutions rather than patients. Thus, this study will use a cluster design; specifically addressing whether an institutional policy of limited use of benzodiazepines during surgery (compared to liberal use) reduces post-operative delirium. The main challenge of a cluster-randomized trial is the substantial loss of statistical power that occurs as a result of clustering. This is because individuals being studied within a cluster are usually more similar than individuals across clusters, a phenomenon described statistically by the intra-cluster correlation coefficient (ICC).[35] This study will use multiple crossovers between intervention arms within each cluster to minimize the loss of statistical power, with a design known as the randomized cluster crossover trial. [36]

In the planned trial, each institution is randomized to one of the two policies of benzodiazepine use (limited or liberal) which they use as standard institutional policy for four weeks. At the end of each period, sites are re-randomized to a new standard policy that is used for the subsequent four weeks. Each site thus acts as its own control group, which mitigates the effect of imbalance between sites in patient and provider characteristics.[35] This design is methodologically rigorous and tests the effect of a change in standard policy, as it would actually be used in the clinical setting.

2. OBJECTIVES

2.1 Primary Objective

The primary objective of the B-Free trial is to evaluate the impact of an institutional policy of limited benzodiazepine use during cardiac surgery, as compared to a policy of liberal benzodiazepine use during cardiac surgery, on the incidence of delirium up to 72 hours after cardiac surgery as measured in routine clinical care using either the Confusion Assessment Method-ICU (CAM-ICU) or the Intensive Care Delirium Screening Checklist (ICDSC).

2.2 Secondary Objectives

The secondary objectives of the B-Free trial are to evaluate the impact of these policies on:

1. ICU length of stay (LOS)
2. Hospital LOS
3. All cause in-hospital mortality

3. STUDY DESIGN

3.1 Type of Study

B-Free is a multicenter, cluster-randomized, crossover trial.

3.2 Expected Number of Clusters and Patients
The expected number of hospitals for the trial is 20, with an average annual case volume of 900, for a total of approximately 18,000 patients.

3.3 Method of Intervention Allocation
Each site will be randomized to one of the two policies to be used as per institutional policy by all anesthetists. The policies to be tested do not require that all patients be treated identically as they allow for reasonable exceptions. Sites will be randomized to one policy and then cross-over to the other policy at set times. Sites will be randomized to twelve, 4-week crossover periods, blocking in periods of 2 to minimize period effects (See Appendix 1: Sample Site Randomization Schedule). Based on the results of our interim analysis, select sites will be randomized to as many as 6 additional crossover periods, dependent on site-level feasibility of doing so. Randomization for all periods will take place in advance of site start-up, but clusters will only be notified of the subsequent period's standard policy during the last week of each crossover period.

3.4 Methods for Protecting Against Bias
As this is a pragmatic trial all patients treated during each period of study will be included in all analyses. Individuals collecting delirium data after cardiac surgery will not be informed of the institutional policy of benzodiazepine currently in use in the operating room at the institution. By including a large number of clusters undergoing multiple crossovers and blocking in periods of two, we will minimize the bias that may occur because of cluster or period effects.

3.5 Duration of Each Intervention Period
The duration of each intervention period is approximately 4 weeks, and all 20 sites complete a total of 12 such 4-week periods (such that each policy is applied 6 times). Based on a mid-study adaptation, 11/20 sites will complete 17 or 18 crossover periods (an additional 5 or 6 periods). Once data is available for 50% of the original sample size, we will undertake a blinded interim analysis that considers the observed event rate, ICC, and IPC. We will use the results of this interim analysis to determine whether an additional period of data collection is required to ensure statistical power.

3.6 Duration of Follow-up
Patients will be followed until their hospital discharge.

4. CRITERIA FOR INCLUSION OF A HOSPITAL

1. Tertiary cardiac surgical center
2. Equipoise by the hospital physicians regarding the use of benzodiazepines during surgery (≥ 95% of hospital cardiac anesthesia group agrees to manage patients as per the benzodiazepine policy in place during a given crossover period)
3. Patients are routinely assessed for postoperative delirium at least once every 12 hours in the ICU after cardiac surgery as a part of routine clinical care using either the Confusion Assessment Method-ICU (CAM-ICU) or the Intensive Care Delirium Screening Checklist (ICDSC)

5. BENZODIAZEPINE POLICIES TO BE STUDIED

Limited Benzodiazepine Policy*:*

1. No routine use of any intraoperative benzodiazepines.
2. Accepted benzodiazepine use in the case of seizure, alcohol withdrawal, severe anxiety, history of awareness during anesthesia, or known benzodiazepine dependence.
3. Accepted benzodiazepine use in patients who are hemodynamically unstable and/or have cardiac anatomy that puts them at high risk of developing ischemia on induction of anesthesia using other agents.

Liberal Benzodiazepine Policy:

1. Administration of benzodiazepine as per clinical guidelines but no lower than 0.03 mg/kg (ideal body weight midazolam equivalent) to all patients undergoing cardiac surgery. Any benzodiazepine may be used.
2. Accepted avoidance of benzodiazepine use in patients who have contraindications to the administration of these medications (i.e., documented allergy, previous adverse reaction to benzodiazepine).

5.1 Pre- and Post-operative Benzodiazepine Administration

Benzodiazepine administration before and after surgery will be minimized during both policy implementation periods in accordance with current guidelines. Recognized exceptions to this include patients who are benzodiazepine dependent, alcohol dependent, or having seizures.

5.2 Study Data Collection

Data will be collected from hospital administrative databases, chart reviews and/or electronic medical records. Data collected will include key baseline characteristics such as demographics, details of surgery, postoperative delirium, and pre- and postoperative medications. Post-operative delirium data will be collected up to 72 hours after cardiac surgery. Encryption of patient identifiers will be used to ensure confidentiality. Investigative sites will transfer the data to PHRI for central data management. Data transfer specification and specific data points to be collected will be detailed in a separate Study Operations Manual.

6. STUDY OUTCOMES

6.1 Primary Outcome

The primary study outcome is the percentage of patients who develop delirium up to 72 hours after cardiac surgery.

6.2 Secondary Outcomes

1. ICU LOS
2. Hospital LOS
3. In-hospital mortality

6.3 Outcome Definitions

1. Assessment of delirium: Delirium will be measured using either the Confusion Assessment Method-ICU (CAM-ICU) or the Intensive Care Delirium Screening Checklist (ICDSC).
2. ICU LOS: This is defined as the number of hours in the cardiac surgical ICU following index cardiac surgery until ICU discharge.
3. Hospital LOS: This is defined as the number of days from index cardiac surgery until hospital discharge.
4. In-hospital mortality: This is defined as death from any cause after the index cardiac surgical procedure and until hospital discharge.

7. STATISTICAL CONSIDERATIONS

7.1 Analysis Population

The modified intention-to-treat population will include all adult (≥ 18 years) patients who underwent cardiac surgery during the trial period at each included cluster and were assessed at least once for delirium in the 72 hours after surgery. The per-protocol population is defined at both the period level (all periods where the allocated policy to ≥80% of patients during every crossover period) and the individual level (all patients who received or did not receive benzodiazepines pre-, intra-, and postoperatively according to the policy period in which they underwent surgery).

7.2 Statistical Methods

The primary analyses will be based on a modified intention-to-treat principle, i.e., participants will be analyzed according to the policy in use when they underwent surgery, regardless of whether or not they were managed according to the policy. Missing values will be treated as 'missing;' no attempt will be made to impute post-randomization values and only observed values will be used for analysis. Standard methods will be used to report tabular and graphical summaries as appropriate for continuous and categorical variables. Summaries of continuous variables will include the number of subjects (N), mean (standard deviation), and median (25th and 75th percentiles). Frequency distributions (N and %) will be reported for categorical data.

All analyses will take place at the individual-patient level. Analyses will be carried out comparing event rates in patients managed during the restricted compared to the liberal benzodiazepine policy periods. Primary and secondary outcomes will be compared between treatment allocation using a logistic mixed model for binary outcomes and linear mixed model for continuous outcomes adjusted for cluster and cluster-by-period as random effect terms.[17] We will report odds ratios and 95% confidence intervals. We will claim statistical significance for treatment effectiveness if the p value is less than 0.05 for the primary outcome. For sensitivity analyses, we will assess for treatment effect heterogeneity across periods, clusters, and clusters with a different number of periods for the primary outcome. We will use SAS 9.4 for UNIX (SAS Institute Inc., Cary, North Carolina, USA) or other validated software for all analyses.

7.3 Planned Subgroup Analyses

We will evaluate the following subgroups of interest: sex, age, benzodiazepine dose, patients who receive no preoperative benzodiazepines, urgent/emergent surgery, and patients with a history of benzodiazepine use or alcohol abuse, both separately and together. The subgroup analyses will be

conducted using tests for interactions in a mixed regression model for the primary and secondary outcomes. We will consider subgroup effects potentially credible if an interaction p value <0.05.

7.4 Original Sample Size Calculation

Based on our initial sample size calculation, we required 16 hospitals with an overall average annual case volume of 1000 patients to complete 12, 4-week crossover periods to achieve statistical power >80%. This number of clusters and patients would the detection of a relative risk reduction of 15% based on our assumptions of a control delirium rate of 15%, an intracluster correlation coefficient (ICC) of 0.02, interperiod correlation (IPC) of 0.5*ICC (i.e., 0.01), and type I error of 5%.

The intracluster correlation coefficient describes the similarity in outcome within a cluster, and the variance in outcome that exists across clusters. The ICC has previously been shown to be related to outcome prevalence in clustered, binary data.[13] We thus used local delirium rates to estimate an ICC of 0.02, based on values determined by Gulliford et al.[13] The interperiod correlation coefficient describes the variance in outcome between individuals from the same cluster across different periods. It is typically difficult to obtain estimates of the IPC,[14] and, as has previously been described, we used an assumed value that was half the magnitude of the ICC, consistent with the recommended standard.[13,15,16]

7.5 Interim analysis

An independent Data Safety Monitoring Board performed an interim analysis to assess efficacy and safety was completed on May 25, 2022, based on data obtained as of May 9, 2022. The interim analysis was originally planned for when 50% of data was available but, due to scheduling conflicts and data entry delays, was completed when 70% of patients had been enrolled. A modified Haybittle-Peto approach was used to evaluate both the primary outcome (delirium incidence) and the secondary outcome of in-hospital mortality. The DSMB was instructed to recommend early trial termination if there was a reduction in delirium in favour of either policy that met the statistical criterion of 3 standard deviations.

7.6 Adaptation to original design based on results of interim analysis

The parameters which play a critical role for accuracy of the B-Free sample size estimation are the control event rate and variance in event rate across clusters and periods (i.e., ICC and IPC). To take emerging data into account, we undertook blinded sample size re-estimation (bSSR) without unblinding the treatment effect. This approach is considered effective to adjust the sample size to achieve the desired power at the end of trial without seriously inflating type I error. Specifically, we used data collected prior to interim analysis to estimate the uncertain parameters used in the original sample size calculation based on a proportion (50%) of the *a priori* required sample size. The steps we undertook can be summarized as follows: 1) calculate sample size; 2) collect a proportion of data; 3) estimate the parameters; 4) re-calculate sample size; 5) increase the number of periods if needed.

Modified sample size: Given 50% of the required sample size, the overall delirium rate was 16.2%, the ICC was 0.04, and the IPC was 0.25*ICC (i.e., 0.01). Taking into consideration of a treatment effect of 15% reduction on the delirium rate proposed at the design stage, we assumed a control rate of 17.5%. Furthermore, because of decreases in cardiac surgery case volume related to the COVID-19 pandemic, the average size of each cluster was 750. With current sample size (i.e., 20 clusters, 12 periods, 750

patients per cluster), the trial would be underpowered with 64% power to detect a 15% reduction. To re-estimate the sample size based observed parameters, we propose for 11 clusters to complete up to 6 additional crossover periods dependent on local feasibility. Based on the results of our adaptive analysis, our modified sample size includes 9 hospitals completing 18 periods, 2 hospitals completing 17 periods, and 9 hospitals completing 12 periods, with an overall average of 900 cardiac surgery patients per cluster and a control delirium rate of 17%, assuming an ICC of 0.06, IPC of 0.03, and type I error of 5%.

Table 1: Assumed, Observed, and Recalculated Sample Size Calculations

	RRR	Average cluster size	Control delirium incidence	# Periods	ICC	IPC	Total n (projected)	# clusters	Alpha	Power
Assumed	15%	1000	0.15	12	0.02	0.01	16000	16	5%	0.8
Observed	-	750	0.17	12	0.06	0.03	15000	20	-	0.70
Recalculated	15%	1000	0.17	12	0.06	0.03	20000	20	5%	0.74[1]
	15%	900	0.17	15	0.06	0.03	18000	20	5%	0.81[2]
	15%	1250	0.17	18	0.06	0.03	22500	20	5%	0.88[3]

[1]Reflects statistical power with pre-pandemic case volumes and observed ICC/IPC.
[2]Proposed adaptation. Number of crossover periods reflects average across all clusters (i.e., 18 in 9 sites, 17 in 2 sites, 12 in 9 sites).
[3]Statistical power using observed ICC/IPC if all sites completed 18 periods

8. DATA MANAGEMENT

Data management will be performed by the PHRI in accordance with PHRI standards and procedures for collection and validation of data. Data will be collected directly through the hospital electronic medical record (EMR) database and on electronic case report forms (eCRFs) using an electronic data capture system, based on the availability of data in the site's EMR. Data for each patient that is entered into the eCRF will be verified by the Investigator. It is the Investigator's responsibility to ensure the accuracy, completeness, legibility, and timeliness of the data reported on the patient's eCRF. All data will be kept secure and confidentiality of all study patients will be carefully protected. Data will be validated, managed, and stored in a de-identified database on a secure server at PHRI.

9. STUDY ORGANIZATION

The Population Health Research Institute (PHRI), a joint institute of McMaster University and Hamilton Health Sciences, is the study Sponsor and will be the study coordination centre responsible for the trial organization, coordination of the international centres, as well as data management and analysis.

The Steering Committee will be responsible for the design, execution, analysis, and reporting of the study. This committee will convene regularly by telephone conference or meetings to address issues and to monitor study progress, execution, and management. The Steering Committee will include the Principal Investigators, several key investigators, and two patient partners. The Steering Committee will hold the primary responsibility for publication of the study results on behalf of the B-Free investigators.

10. ETHICAL STANDARDS

10.1 Ethical Considerations

The study will be conducted in accordance with Good Clinical Practice (GCP), all applicable subject privacy requirements, and the guiding principles of the Declaration of Helsinki, including, but not limited to:

- Institutional Review Board (IRB)/Research Ethics Board (REB) review and approval of study protocol and any subsequent amendments.
- A modified consent process approved by the IRB.

10.2 Waiver of Individual Patient Consent

The cluster design challenges conventional approaches to clinical research because patients cannot choose to avoid the intervention, nor consent for the study because the intervention is applied at the level of the health care environment and not the patient. B-Free evaluates two different cardiac anesthesia policies related to the use of benzodiazepines (minimized versus liberal intraoperative administration). Centres will only be included if there is physician equipoise regarding the benzodiazepine policies being tested in this study. The different approaches to the use of benzodiazepine, embodied in the two study policies being evaluated, are both of minimal risk and are commonly used in Canada and other countries[10]. We will obtain waiver of individual patient consent at each participating site before study initiation, according to criteria proposed by the Tri-Council Policy Statement (TCPS 2): Ethical Conduct for Research Involving Humans[37,38]. This study fulfills TCPS 2 criteria in that: (i) the study poses minimal risk to patients; (ii) waiver of consent will not adversely affect patient rights and welfare; (iii) it would be impracticable to carry out the research if prior consent is required; and (iv) patients and/or families will be provided with information about the study using an information letter and/or poster in ICU waiting areas.

10.3 Ethics Review and Approval

Local investigators must submit this protocol to an Ethics Review Committee or a similar body (IRB, REB) and is required to forward a copy of the written approval/advice signed by the Chair to PHRI. On the approval/advice sheet, the trial name and protocol version, the study documents (protocol and letter of information), and the date of the review should be clearly stated.

11. INVESTIGATOR AND ADMINISTRATIVE REQUIREMENTS

11.1 Investigator Responsibilities

The Investigator will allow representatives of PHRI to periodically conduct relevant audits of the investigative site. The monitoring visits provide PHRI with the opportunity to evaluate the progress of the study; ensure that all protocol requirements, applicable regulations, and Investigator's obligations are being fulfilled; and resolve any issues that may arise.

The Investigator(s) undertake(s) to conduct the study in accordance with Good Clinical Practice. The Investigator is required to ensure adherence with respect to implementation of the two policies according to the randomization schedule and procedures required by the protocol.

11.2 Confidentiality and Disclosure of Data

All goods, materials, information (oral or written) and unpublished documentation provided to the Investigators, inclusive of this protocol, are the exclusive property of the Sponsor. They may not be given or disclosed by the Investigator or by any person within their authority either in part or in totality to any unauthorized person without the prior written formal consent of the Sponsor. It is specified that the submission of this protocol and other necessary documentation to the IRB or a like body is expressly permitted, the IRB members having the same obligation of confidentiality.

The Investigator shall consider as confidential and shall take all necessary measures to ensure that there is no breach of confidentiality in respect of all information accumulated, acquired or deduced in the course of the trial, other than that information to be disclosed by law.

Any personal health information obtained as a result of this study is considered confidential and disclosure to third parties other than those noted below is prohibited. The study personnel, employees of the regulatory agencies, including Health Canada and the study sponsor, PHRI, and its agents may need to review patient medical records in order to accurately record information for this study. If results of this study are reported in medical journals or at meetings, the patient's identity will remain confidential.

11.3 Record Retention

The Investigator must retain trial records for the amount of time specified by applicable laws and regulations or by ICH E6 Good Clinical Practice guidelines, whichever is longer. In Canada, retention of all records created during the conduct of a clinical trial is 25 years. All trial documents shall be made available upon request from relevant health authorities. Any investigative center will consult the PHRI Coordinating Centre before discarding trial and/or subject files.

12. OWNERSHIP OF DATA AND USE OF STUDY RESULTS

The Sponsor and B-Free Steering Committee of the study have the ownership of all data and results collected during this study. In consequence, the Sponsor reserves the right to use the data of the present study, with or without comments and with or without analysis, in order to submit them to the Health Authorities of any country. Full publication rights of the study data solely reside with the Sponsor and Steering Committee.

13. PUBLICATION POLICY

All study presentations and/or publication of the results will be based on clean, checked and validated data in order to ensure the accuracy of the results. All analyses for publication will be provided by the PHRI Coordinating Centre. The responsibility for presentations and/or publications belongs to the Steering Committee. The final content of the manuscript is the responsibility of the Steering Committee. Publication of the main findings of this study will be made jointly in the name of all collaborators. Other papers will be authored based on the contributions of the individuals to the overall study. All the trial participants (Investigators and committee members) make a prior delegation of responsibility for primary presentation and/or primary publication of the results to the Steering Committee. No other publication is allowed before the primary publication. Any presentation or publication by any trialist

must mention the trial and has to be approved by the Steering Committee. Moreover, it is mandatory to make reference to the primary publication.

REFERENCES

1. Inouye SK, Westendorp RG, Saczynski JS. Delirium in elderly people. Lancet 2014;383:911-22.
2. Marcantonio ER, Juarez G, Goldman L, et al. The relationship of postoperative delirium with psychoactive medications. JAMA 1994;272:1518-22.
3. American Geriatrics Society Expert Panel on Postoperative Delirium in Older Adults. Postoperative delirium in older adults: best practice statement from the American Geriatrics Society. J Am Coll Surg 2015;220:136-48 e1.
4. Dasgupta M, Dumbrell AC. Preoperative risk assessment for delirium after noncardiac surgery: a systematic review. J Am Geriatr Soc 2006;54:1578-89.
5. Gleason LJ, Schmitt EM, Kosar CM, et al. Effect of Delirium and Other Major Complications on Outcomes After Elective Surgery in Older Adults. JAMA surg 2015:1-7.
6. Saczynski JS, Marcantonio ER, Quach L, et al. Cognitive trajectories after postoperative delirium. N Engl J Med 2012;367:30-9.
7. Leslie DL, Marcantonio ER, Zhang Y, Leo-Summers L, Inouye SK. One-year health care costs associated with delirium in the elderly population. Arch Intern Med 2008;168:27-32.
8. Redelmeier DA, Thiruchelvam D, Daneman N. Delirium after elective surgery among elderly patients taking statins. Can Med Assoc J 2008;179:645-52.
9. Pandharipande P, Shintani A, Peterson J, et al. Lorazepam is an independent risk factor for transitioning to delirium in intensive care unit patients. Anesthesiology 2006;104:21-6.
10. Pandharipande PP, Pun BT, Herr DL, et al. Effect of sedation with dexmedetomidine vs lorazepam on acute brain dysfunction in mechanically ventilated patients: the MENDS randomized controlled trial. JAMA 2007;298:2644-53.
11. Taipale PG, Ratner PA, Galdas PM, et al. The association between nurse-administered midazolam following cardiac surgery and incident delirium: an observational study. Int J Nurs Stud 2012;49:1064-73.
12. Riker RR, Shehabi Y, Bokesch PM, et al. Dexmedetomidine vs midazolam for sedation of critically ill patients: a randomized trial. JAMA 2009;301:489-99.
13. Gunther ML, Morandi A, Ely EW. Pathophysiology of delirium in the intensive care unit. Crit Care Clin 2008;24:45-65, viii.
14. Berian JR, Zhou L, Russell MM, et al. Postoperative Delirium as a Target for Surgical Quality Improvement. Ann Surg 2018;268:93-9.
15. Devlin JW, Skrobik Y, Gelinas C, et al. Clinical Practice Guidelines for the Prevention and Management of Pain, Agitation/Sedation, Delirium, Immobility, and Sleep Disruption in Adult Patients in the ICU. Crit Care Med 2018;46:e825-e73.
16. Andrews L, Silva SG, Kaplan S, Zimbro K. Delirium monitoring and patient outcomes in a general intensive care unit. Am J Crit Care 2015;24:48-56.
17. van den Boogaard M, Pickkers P, van der Hoeven H, Roodbol G, van Achterberg T, Schoonhoven L. Implementation of a delirium assessment tool in the ICU can influence haloperidol use. Crit Care 2009;13:R131.
18. Bigatello LM, Amirfarzan H, Haghighi AK, et al. Effects of routine monitoring of delirium in a surgical/trauma intensive care unit. J Trauma Acute Care Surg 2013;74:876-83.
19. Devlin JW, Fong JJ, Schumaker G, O'Connor H, Ruthazer R, Garpestad E. Use of a validated delirium assessment tool improves the ability of physicians to identify delirium in medical intensive care unit patients. Crit Care Med 2007;35:2721-4; quiz 5.

20. Spronk PE, Riekerk B, Hofhuis J, Rommes JH. Occurrence of delirium is severely underestimated in the ICU during daily care. Intens Care Med 2009;35:1276-80.
21. Maldonado JR, Wysong A, van der Starre PJ, Block T, Miller C, Reitz BA. Dexmedetomidine and the reduction of postoperative delirium after cardiac surgery. Psychosom 2009;50:206-17.
22. Barr J, Fraser GL, Puntillo K, et al. Clinical practice guidelines for the management of pain, agitation, and delirium in adult patients in the intensive care unit. Crit Care Med 2013;41:263-306.
23. Ferrell BA, Girard TD. Sedative choice: a critical decision. Am J Respir Crit Care Med 2014;189:1295-7.
24. Spence J, Belley-Cote E, Devereaux PJ, et al. Benzodiazepine administration during adult cardiac surgery: a survey of current practice among Canadian anesthesiologists working in academic centres. Can J Anesth 2018;65:263-71.
25. Arenson BG, MacDonald LA, Grocott HP, Hiebert BM, Arora RC. Effect of intensive care unit environment on in-hospital delirium after cardiac surgery. J Thorac Cardiovasc Surg 2013;146:172-8.
26. Pauley E, Lishmanov A, Schumann S, Gala GJ, van Diepen S, Katz JN. Delirium is a robust predictor of morbidity and mortality among critically ill patients treated in the cardiac intensive care unit. Am Heart J 2015;170:79-86, e1.
27. Kazmierski J, Kowman M, Banach M, et al. Incidence and predictors of delirium after cardiac surgery: Results from The IPDACS Study. J Psychosom Res 2010;69:179-85.
28. American Society of Anesthesiologists Task Force on Intraoperative Awareness. Practice advisory for intraoperative awareness and brain function monitoring: a report by the american society of anesthesiologists task force on intraoperative awareness. Anesthesiology 2006;104:847-64.
29. Myles PS, Symons JA, Leslie K. Anaesthetists' attitudes towards awareness and depth-of-anaesthesia monitoring. Anaesthesia 2003;58:11-6.
30. Serfontein L. Awareness in cardiac anesthesia. Curr Opin Anesthesiol 2010;23:103-8.
31. Sandin RH, Enlund G, Samuelsson P, Lennmarken C. Awareness during anaesthesia: a prospective case study. Lancet 2000;355:707-11.
32. Phillips AA, McLean RF, Devitt JH, Harrington EM. Recall of intraoperative events after general anaesthesia and cardiopulmonary bypass. Can J Anesth 1993;40:922-6.
33. Ranta S, Jussila J, Hynynen M. Recall of awareness during cardiac anaesthesia: influence of feedback information to the anaesthesiologist. Acta anaesthesiol Scand 1996;40:554-60.
34. Myles PS, Leslie K, McNeil J, Forbes A, Chan MT. Bispectral index monitoring to prevent awareness during anaesthesia: the B-Aware randomised controlled trial. Lancet 2004;363:1757-63.
35. Arnup SJ, Forbes AB, Kahan BC, Morgan KE, McKenzie JE. Appropriate statistical methods were infrequently used in cluster-randomized crossover trials. J Clin Epidemiol 2016;74:40-50.
36. Connolly SJ, Philippon F, Longtin Y, et al. Randomized cluster crossover trials for reliable, efficient, comparative effectiveness testing: design of the Prevention of Arrhythmia Device Infection Trial (PADIT). Can J Cardiol 2013;29:652-8.
37. Canadian Institutes of Health Research, Natural Sciences and Engineering Research Council of Canada, and Social Sciences and Humanities Research Council of Canada. Tri-Council Policy Statement: Ethical Conduct for Research Involving Humans, December 2014. Available from: http://www.pre.ethics.gc.ca/eng/policy-politique/initiatives/tcps2-eptc2/Default/. Accessed May 23, 2018.
38. United States Food and Drug Administration (USFDA). IRB Waiver or Alteration of Informed Consent for Clinical Investigations Involving No More Than Minimal Risk to Human Subjects. Available

from: https://www.fda.gov/downloads/RegulatoryInformation/Guidances/UCM566948.pdf. Accessed May 23, 2018.

Protocol Change Summary

Documentation of revisions made to **Protocol v3.0 2019-04-25** that became **Protocol v4.0 2019-08-08**

Principal Investigator:
Dr. Jessica Spence, MD, PhD
Population Health Research Institute
DBCVSRI, 237 Barton Street East
Hamilton, Ontario, Canada L8L 2X2

Study Coordinating Group:
B-Free Project Office
Population Health Research Institute
20 Copeland Av
Hamilton, Ontario, Canada L8L 2X2

Protocol Number: 2019.08.08
ClinicalTrials.gov Identifier: NCT03928236

This protocol has been developed by the B-Free Project Office and its contents are the intellectual property of this group. It is an offence to reproduce or use the information and data in this protocol for any purpose other than the B-Free Trial without prior approval from the Project Office of the B-Free Trial.

B-Free Protocol Amendment v4.0 Approval:

By signing the below, I designate my approval of the above-named version of the B-Free protocol amendment.

Dr. Jessica Spence<br>Principal Investigator<br>Population Health Research Institute	DocuSigned by:<br>Jessica Spence<br>Signature<br>Signer Name: Jessica Spence<br>Signing Reason: I approve this document<br>Signing Time: 2024-01-24 | 12:04:12 PM EST<br>E06CC11CE8984853983B0EAD7101A574	2024-01-24 | 12:04:18 PM EST<br>Date (yyyy-mm-dd)

1. RATIONALE FOR CHANGES BETWEEN PROTOCOL v3.0 and v4.0

1. The primary objective was updated to capture postoperative delirium in the ICU up to 72 hours. This was to provide further clarification of study procedures and study definitions and to specify where delirium will be assessed.

2. The primary outcome was updated to state the proportion rather than the percentage of patients with delirium during the postoperative ICU stay up to 72 hours. This was to provide further clarification of study procedures and study definitions and to clarify that delirium will be categorized via the ICU visit.

3. Updated section 5.2 to further define how data will be collected, specifically stating that postoperative delirium data will be collected up to 72 hours after ICU admission or until ICU discharge. This was updated to provide further clarification on how data is collected and for how long.

2. DESCRIPTION OF CHANGES BETWEEN v3.0 and v4.0

PAGES 9-10
THE FOLLOWING TEXT IS MODIFIED (STUDY SYNOPSIS)

**Title**	Benzodiazepine-free cardiac anesthesia for the reduction of postoperative delirium (B-Free)
**Study Objectives**	The primary objective of the B-Free trial is to evaluate the impact of an institutional policy of limited benzodiazepine use during cardiac surgery, as compared to a policy of liberal benzodiazepine use during cardiac surgery, on the incidence of delirium during the ~~initial~~postoperative intensive care unit (ICU) stay up to 72 hours~~ after cardiac surgery~~.<br><br>The secondary objectives are to evaluate the impact of these policies on:<br>1. ICU length of stay (LOS)<br>2. Hospital LOS<br>3. All cause in-hospital mortality
**Study Design**	A multi-centre, randomized cluster crossover trial.
**Hospital Eligibility:**	1. Major surgical center with a minimum of 500 cases of cardiac surgery per year<br>2. Equipoise by the hospital physicians regarding the use of benzodiazepines during surgery (≥ 95% of hospital cardiac anesthesia group agrees to manage patients as per the benzodiazepine policy in place during a given crossover period)<br>3. Patients are routinely assessed for postoperative delirium at least once every 12 hours ~~during the initial 72 hours after cardiac~~

	~~surgery~~after ICU admission as a part of routine clinical care using either the Confusion Assessment Method-ICU (CAM-ICU) or the Intensive Care Delirium Screening Checklist (ICDSC).
**Total number of hospitals (clusters)**	16 hospitals, each with an average annual case volume of 1,000 patients, will be included in the trial.
**Expected number of subjects**	Approximately 16,000 adult patients undergoing cardiac surgery.
**Study Intervention**	The 'Limited Benzodiazepine Policy' consists of the following:<br>1. No routine use of any intraoperative benzodiazepines.<br>2. Accepted benzodiazepine use in the case of seizure, alcohol withdrawal, severe anxiety, history of awareness during anesthesia, or known benzodiazepine dependence.<br>3. Accepted benzodiazepine use in patients who are hemodynamically unstable and/or have cardiac anatomy that puts them at high risk of developing ischemia on induction of anesthesia using other agents.
**Study Comparator**	The 'Liberal Benzodiazepine Policy' consists of the following:<br>1. Administration of benzodiazepine as per clinical guidelines but no lower than 0.03 mg/kg (ideal body weight midazolam equivalent) to all patients undergoing cardiac surgery. Any benzodiazepine may be used.<br>2. Accepted avoidance of benzodiazepine use in patients who have contraindications to the administration of these medications (i.e. documented allergy, previous adverse reaction to benzodiazepine).
**Primary Outcome**	The primary study outcome is the ~~percentage~~proportion of patients with delirium ~~assessed~~ during the ~~initial~~postoperative intensive care unit (ICU) stay up to 72 hours~~ following cardiac surgery~~.
**Statistical Analysis**	The primary analysis will be based on the intention to treat principle, all patients (aged ≥18 years) undergoing cardiac surgery will be included in the analysis treated during a period regardless of whether or not they were managed according to the policy in place during the period. Analyses will be carried out comparing event rates in patients managed during the benzodiazepine liberal and limited arms). The primary and secondary outcomes will be estimated between the treatment groups using a hierarchical mixed model for binary outcomes (i.e. GLIMMIX) adjusted for cluster and cluster-by-period as random effects terms (Turner et al., 2007). A sensitivity analysis will be conducted to evaluate the primary outcome in patients managed per protocol. The odds ratios and 95% confidence intervals will be reported. Statistical significance will be claimed if the p value is less than 0.05 for the primary outcome for treatment effectiveness.<br>Carryover effects will be assessed for the primary outcomes using tests for interactions between periods and treatment effects using hierarchical mixed models.

**Duration of Study Period (per cluster)**	Each hospital (cluster) will participate in the trial for twelve, four-week crossover periods.

PAGE 8
THE FOLLOWING TEXT IS MODIFIED (ABBREVIATIONS):

CAM-ICU	Confusion Assessment Method-ICU
CI	Confidence Interval
CNS	Central Nervous System
CPB	Cardiopulmonary Bypass
CVICU	Cardiovascular Intensive Care Unit
DSMB	Data Safety Monitoring Board
eCRF	Electronic Case Report Form
EMR	Electronic Medical Record
FDA	United States Food and Drug Administration
GABA	Gamma-aminobutyric Acid
GCP	Good Clinical Practice
ICC	Intra-cluster Correlation Coefficient
ICDSC	Intensive Care Delirium Screening Checklist
ICH	International Council for Harmonisation
ICU	Intensive Care Unit
IEC	Independent Ethics Committee
IPC	Interperiod Correlation Coefficient
IRB	Institutional Review Board
LOS	Length of Stay
PHRI	Population Health Research Institute
PRE	Government of Canada Panel on Research Ethics
REB	Research Ethics Board
SCCM	Society for Critical Care Medicine
TCPS	Tri-Council Policy Statement

PAGE 14
THE FOLLOWING TEXT IS MODIFIED (2 OBJECTIVES)

Primary Objective
The primary objective of the B-Free trial is to evaluate the impact of an institutional policy of limited benzodiazepine use during cardiac surgery, as compared to a policy of liberal benzodiazepine use during

cardiac surgery, on the incidence of delirium during the ~~initial~~postoperative intensive care unit (ICU) stay up to 72 hours~~ after cardiac surgery~~.

PAGE 15
THE FOLLOWING TEXT IS MODIFIED (4 CRITERIA FOR INCLUSION OF A HOSPITAL)

1. Major surgical center with a minimum of 500 cases of cardiac surgery per year
2. Equipoise by the hospital physicians regarding the use of benzodiazepines during surgery (≥ 95% of hospital cardiac anesthesia group agrees to manage patients as per the benzodiazepine policy in place during a given crossover period)
3. Patients are routinely assessed for postoperative delirium at least once every 12 hours ~~during~~in the ~~initial 72 hours~~ICU after cardiac surgery as a part of routine clinical care using either the Confusion Assessment Method-ICU (CAM-ICU) or the Intensive Care Delirium Screening Checklist (ICDSC)

PAGE 16
THE FOLLOWING TEXT IS MODIFIED (5.2 DATA COLLECTION)

Data will be collected from hospital administrative databases, chart reviews and/or electronic medical records. Data collected will include key baseline characteristics such as demographics, details of surgery, postoperative delirium, and pre- and postoperative medications. Post-operative delirium data will be collected up to 72 hours after ICU admission or until ICU discharge. Encryption of patient identifiers will be used to ensure confidentiality. Investigative sites will transfer the data to PHRI for central data management. Data transfer specification and specific data points to be collected will be detailed in a separate Study Operations Manual.

PAGE 16
THE FOLLOWING TEXT IS MODIFIED (6 STUDY OUTCOMES)

Primary Outcome
The primary study outcome is the percentage of patients with delirium assessed during the ~~initial~~postoperative intensive care unit (ICU) stay up to 72 hours~~ following cardiac surgery~~.

Protocol Change Summary

Documentation of revisions made to **Protocol v4.0 2019-08-08** that became **Protocol v5.0 2023-02-13**

Principal Investigator:
Dr. Jessica Spence, MD, PhD
Population Health Research Institute
20 Copeland Av
Hamilton, Ontario, Canada L8L 2X2

Study Coordinating Group:
B-Free Project Office
Population Health Research Institute
20 Copeland Av
Hamilton, Ontario, Canada L8L 2X2

Protocol Number: 2023.02.13
ClinicalTrials.gov Identifier: NCT03928236

This protocol has been developed by the B-Free Project Office and its contents are the intellectual property of this group. It is an offence to reproduce or use the information and data in this protocol for any purpose other than the B-Free Trial without prior approval from the Project Office of the B-Free Trial.

B-Free Protocol Amendment v5.0 Approval:

By signing the below, I designate my approval of the above-named version of the B-Free protocol amendment.

Dr. Jessica Spence<br>Principal Investigator<br>Population Health Research Institute	DocuSigned by:<br>Jessica Spence<br>Signature<br>Signer Name: Jessica Spence<br>Signing Reason: I approve this document<br>Signing Time: 2024-01-25 | 2:25:10 PM EST<br>E06CC11CE8984853983B0EAD7101A574	2024-01-25 | 2:25:15 PM EST<br>Date (yyyy-mm-dd)

1. RATIONALE FOR CHANGES BETWEEN PROTOCOL v4.0 and v5.0

1. The primary objective was updated to include the capture of postoperative delirium up to 72 hours post-surgery, not to be capture only during ICU. This was to provide further clarification of study procedures and study definitions and to include delirium assessments outside of the ICU.

2. Hospital eligibility was updated to include tertiary cardiac surgical centers, to account for some hospitals whose status changed as a result of a decrease in cases due to COVID. This did not result in a change in hospitals who participated; their individual classification changed throughout the course of the trial.

3. The primary outcome was updated to remove the specificity of the ICU. This was to reflect the delirium captured inside AND outside of the ICU.

4. Section 7.1 Analysis Population was updated to reflect intention-to-treat population.

5. Section 7.2 was updated to further define the statistical methods used for analysis.

6. Section 7.6 was added to explain the adaptation to the original design based on the results of the interim analysis.

2. DESCRIPTION OF CHANGES BETWEEN v4.0 and v5.0

PAGES 10-12
THE FOLLOWING TEXT IS MODIFIED (STUDY SYNOPSIS)

**Title**	Benzodiazepine-free cardiac anesthesia for the reduction of postoperative delirium (B-Free)
**Study Objectives**	The primary objective of the B-Free trial is to evaluate the impact of an institutional policy of limited benzodiazepine use during cardiac surgery, as compared to a policy of liberal benzodiazepine use during cardiac surgery, on the incidence of delirium ~~during the postoperative intensive care unit (ICU) stay~~ up to 72 hours after surgery.<br><br>The secondary objectives are to evaluate the impact of these policies on:<br>1. ICU length of stay (LOS)<br>2. Hospital LOS<br>3. All cause in-hospital mortality
**Study Design**	A multi-centre, randomized cluster crossover trial.
**Hospital Eligibility:**	1. ~~Major~~ Tertiary cardiac surgical center ~~with a minimum of 500 cases of cardiac surgery per year~~

	2. Equipoise by the hospital physicians regarding the use of benzodiazepines during surgery (≥ 95% of hospital cardiac anesthesia group agrees to manage patients as per the benzodiazepine policy in place during a given crossover period)<br>3. Patients are routinely assessed for postoperative delirium at least once every 12 hours after ICU admission as a part of routine clinical care using either the Confusion Assessment Method-ICU (CAM-ICU) or the Intensive Care Delirium Screening Checklist (ICDSC).
**Original sample size**	16 hospitals, with an average annual case volume of 1000 patients
**Observed sample size at time of interim analysis**	20 hospitals, with an average case volume of 750 patients
**~~Total number of hospitals (clusters)~~Final sample size based on trial adaptation**	~~16~~20 hospitals, ~~each~~ with an average annual case volume of ~~1,000~~900 patients~~, will be included in the trial.~~
**Expected number of subjects**	Approximately ~~16~~18,000 adult patients undergoing cardiac surgery.
**Study Intervention**	The ‘Limited Benzodiazepine Policy’ consists of the following:<br>1. No routine use of any intraoperative benzodiazepines.<br>2. Accepted benzodiazepine use in the case of seizure, alcohol withdrawal, severe anxiety, history of awareness during anesthesia, or known benzodiazepine dependence.<br>3. Accepted benzodiazepine use in patients who are hemodynamically unstable and/or have cardiac anatomy that puts them at high risk of developing ischemia on induction of anesthesia using other agents.
**Study Comparator**	The ‘Liberal Benzodiazepine Policy’ consists of the following:<br>1. Administration of benzodiazepine as per clinical guidelines but no lower than 0.03 mg/kg (ideal body weight midazolam equivalent) to all patients undergoing cardiac surgery. Any benzodiazepine may be used.<br>2. Accepted avoidance of benzodiazepine use in patients who have contraindications to the administration of these medications (i.e. documented allergy, previous adverse reaction to benzodiazepine).
**Primary Outcome**	The primary study outcome is the proportion of patients with delirium ~~during the postoperative intensive care unit (ICU) stay~~ up to 72 hours after cardiac surgery.
**Statistical Analysis**	The primary analysis will be based on ~~the~~a modified intention to treat principle, all patients (aged ≥18 years) undergoing cardiac surgery who are assessed for delirium will be included in the analysis treated during a period regardless of whether or not they were managed according to

	the policy in place during the period. Analyses will be carried out comparing event rates in patients managed during the benzodiazepine liberal and limited arms). The primary and secondary outcomes will be estimated between the treatment groups using a hierarchical mixed model for binary outcomes (i.e. GLIMMIX) adjusted for cluster and cluster-by-period as random effects terms (Turner et al., 2007). A sensitivity analysis will be conducted to evaluate the primary outcome in patients managed per protocol. The odds ratios and 95% confidence intervals will be reported. Statistical significance will be claimed if the p value is less than 0.05 for the primary outcome for treatment effectiveness.<br>Carryover effects will be assessed for the primary outcomes using tests for interactions between periods and treatment effects using hierarchical mixed models.
**Duration of Study Period (per cluster)**	~~Each hospital (cluster~~All included hospitals (clusters) will participate in the trial for a minimum of twelve, four-week crossover periods. 11/20 hospitals will participate for an additional 5 or 6, four-week crossover periods.

PAGE 16
THE FOLLOWING TEXT IS MODIFIED (2 OBJECTIVES)

2.1 Primary Objective
The primary objective of the B-Free trial is to evaluate the impact of an institutional policy of limited benzodiazepine use during cardiac surgery, as compared to a policy of liberal benzodiazepine use during cardiac surgery, on the incidence of delirium ~~during the postoperative intensive care unit (ICU) stay up to 72 hours.~~ up to 72 hours after cardiac surgery as measured in routine clinical care using either the Confusion Assessment Method-ICU (CAM-ICU) or the Intensive Care Delirium Screening Checklist (ICDSC).

PAGE 17
THE FOLLOWING TEXT IS MODIFIED (3 STUDY DESIGN)

3.2 Expected Number of Clusters and Patients
The expected number of hospitals for the trial is ~~16~~20, with an average annual case volume of ~~1,000~~900, for a total of approximately ~~16~~18,000 patients.

3.3 Method of Intervention Allocation
Each site will be randomized to one of the two policies to be used as per institutional policy by all anesthetists. The policies to be tested do not require that all patients be treated identically as they allow for reasonable exceptions. Sites will be randomized to one policy and then cross-over to the other policy at set times. Sites will be randomized to twelve, 4-week crossover periods, blocking in periods of 2 to minimize period effects (See Appendix 1: Sample Site Randomization Schedule). Based on the results of our interim analysis, select sites will be randomized to as many as 6 additional crossover periods, dependent on site-level feasibility of doing so. Randomization for all periods will take place in advance

of site start-up, but clusters will only be notified of the subsequent period's standard policy during the last week of each crossover period.

3.4 Methods for Protecting Against Bias

As this is a pragmatic trial all patients treated during each period of study will be included in all analyses. Individuals collecting delirium data after cardiac surgery in the ICU will not be informed of the institutional policy of benzodiazepine currently in use in the operating room at the institution. By including a large number of clusters undergoing multiple crossovers and blocking in periods of two, we will minimize the bias that may occur because of cluster or period effects.

3.5 Duration of Each Intervention Period

The duration of each intervention period is approximately 4 weeks; each cluster will be randomized to , and all 20 sites complete a total of 12 such 4-week periods (such that each policy is applied 6 times). Based on a mid-study adaptation, 11/20 sites will complete 17 or 18 crossover periods (an additional 5 or 6 periods). Once data is available for 50% of the original sample size, we will undertake a blinded interim analysis that considers the observed event rate, ICC, and IPC. We will use the results of this interim analysis to determine whether an additional period of data collection is required to ensure statistical power.

PAGE 17

THE FOLLOWING TEXT IS MODIFIED (4 CRITERIA FOR INCLUSION OF A HOSPITAL)

1. Major Tertiary cardiac surgical center with a minimum of 500 cases of cardiac surgery per year

PAGE 18

THE FOLLOWING TEXT IS MODIFIED (5.2 STUDY DATA COLLECTION)

5.2 Study Data Collection

Data will be collected from hospital administrative databases, chart reviews and/or electronic medical records. Data collected will include key baseline characteristics such as demographics, details of surgery, postoperative delirium, and pre- and postoperative medications. Post-operative delirium data will be collected up to 72 hours after ICU admission or until ICU discharge.cardiac surgery. Encryption of patient identifiers will be used to ensure confidentiality. Investigative sites will transfer the data to PHRI for central data management. Data transfer specification and specific data points to be collected will be detailed in a separate Study Operations Manual.

PAGES 18-19

THE FOLLOWING TEXT IS MODIFIED (6.1 PRIMARY OUTCOME)

6.1 Primary Outcome

The primary study outcome is the percentage of patients withwho develop delirium assessed during the postoperative intensive care unit (ICU) stay up to 72 hours after cardiac surgery.

PAGES 19-22
THE FOLLOWING TEXT IS MODIFIED (7 STATISTICAL CONSIDERATIONS)

7.1 Analysis Population
~~All~~The modified intention-to-treat population will include all adult (≥ 18 years) patients ~~undergoing~~who underwent cardiac surgery during the trial period at each included cluster ~~will be included~~and were assessed at least once for delirium in the ~~analysis~~72 hours after surgery. The per-protocol population is defined at both the period level (all periods where the allocated policy to ≥80% of patients during every crossover period) and the individual level (all patients who received or did not receive benzodiazepines pre-, intra-, and postoperatively according to the ~~crossover~~policy period ~~policy~~ in ~~place at the time~~which they ~~undergo surgery, regardless of how they are managed. Each cluster will apply each of the two policies six times during twelve, 4-week crossover periods.~~ underwent surgery).

7.2 Statistical Methods
The primary analyses will be based on ~~the~~a modified intention-to-treat principle, i.e~~.~~., participants will be analyzed according to the policy in use when they underwent surgery, regardless of whether or not they were managed according to the policy. Missing values will be treated as 'missing;' no attempt will be made to impute post-randomization values and only observed values will be used for analysis. Standard methods will be used to report tabular and graphical summaries as appropriate for continuous and categorical variables. Summaries of continuous variables will include the number of subjects (N), mean (standard deviation), and median (25th and 75th percentiles). Frequency distributions (N and %) will be reported for categorical data.

All analyses will take place at the individual-patient level. Analyses will be carried out comparing event rates in patients managed during the restricted compared to the liberal benzodiazepine ~~liberal and limited arms. The primary~~policy periods. Primary and secondary outcomes will be ~~estimated~~compared between ~~the~~ treatment ~~groups~~allocation using a ~~hierarchical~~logistic mixed model for binary outcomes ~~(i.e. GLIMMIX)~~ and linear mixed model for continuous outcomes adjusted for cluster and cluster-by-period as random ~~effects~~effect terms ~~(Turner et al., 2007). A sensitivity analysis will be conducted to evaluate the primary outcome in patients managed per protocol. The~~.[17] We will report odds ratios and 95% confidence intervals. We will ~~be reported. Statistical~~claim statistical significance ~~will be claimed~~for treatment effectiveness if the p value is less than 0.05 for the primary outcome ~~for treatment effectiveness. Carryover effects will be assessed for the primary outcomes using tests for interactions between periods and~~ . For sensitivity analyses, we will assess for treatment ~~effects using hierarchical mixed models.~~effect heterogeneity across periods, clusters, and clusters with a different number of periods for the primary outcome. We will use SAS 9.4 for UNIX (SAS Institute Inc., Cary, North Carolina, USA) or other validated software ~~will be used~~ for all analyses. ~~A comprehensive plan for analysis will be detailed in a separate Statistical Analysis Plan (SAP).~~

7.3 Planned Subgroup Analyses
~~The~~ We will evaluate the following subgroups of interest ~~will be evaluated~~: sex, age, benzodiazepine dose, patients who receive no preoperative benzodiazepines, ~~and~~ urgent/emergent surgery, and patients with a history of benzodiazepine use or alcohol abuse, both separately and together. The subgroup analyses will be conducted using tests for interactions in a mixed regression model for the primary and

secondary outcomes. We will consider subgroup effects potentially credible if an interaction p value <0.05.

7.4 Original Sample Size Calculation

Based on our initial sample size calculation, we required 16 hospitals with an overall average annual case volume of 1000 patients to complete 12, 4-week crossover periods to achieve statistical power >80%. This number of clusters and patients would the detection of a relative risk reduction of 15% based on our assumptions of a control delirium rate of 15%, an intracluster correlation coefficient (ICC) of 0.02, interperiod correlation (IPC) of 0.5*ICC (i.e., 0.01), and type I error of 5%.

The intracluster correlation coefficient describes the similarity in outcome within a cluster, and the variance in outcome that exists across clusters. The ICC has previously been shown to be related to outcome prevalence in clustered, binary data.[13] We thus used local delirium rates to estimate an ICC of 0.02, based on values determined by Gulliford et al.[13] The interperiod correlation coefficient describes the variance in outcome between individuals from the same cluster across different periods. It is typically difficult to obtain estimates of the IPC,[14] and, as has previously been described, we used an assumed value that was half the magnitude of the ICC, consistent with the recommended standard.[13,15,16]

7.4 7.5 Interim Analysisanalysis

OneAn independent Data Safety Monitoring Board performed an interim analysis will be undertaken when half the sites haveto assess efficacy and safety was completed at least 6 periods. For efficacy, aon May 25, 2022, based on data obtained as of May 9, 2022. The interim analysis was originally planned for when 50% of data was available but, due to scheduling conflicts and data entry delays, was completed when 70% of patients had been enrolled. A modified Haybittle-Peto approach will bewas used to evaluate both the primary outcome (delirium incidence) and the secondary outcome of in-hospital mortality. An independent Data Monitoring Committee (DMC) will review the interim analysis data and make recommendations to the study leadership about the conduct of the trial, integrity of the data and trial discontinuation to ensure the overall safety of patients. The guiding policies and operating procedures governing the DMC will be described in a separate DMC charterThe DSMB was instructed to recommend early trial termination if there was a reduction in delirium in favour of either policy that met the statistical criterion of 3 standard deviations.

Sample Size Calculation

In B-Free, the intracluster correlation coefficient (ICC) will depend on the incidence of delirium in each participating cluster and the interperiod correlation coefficient (IPC) will be assumed to be half of ICC. Given an estimated prevalence of delirium of 15% (derived from local administrative data and confirmed in our pilot study at two centres), we estimated a conservative ICC of 0.02, based on values determined by Gulliford et al. using several large administrative data sets. Based on the assumption of an ICC = 0.02, an IPC = 0.5*ICC, a relative risk reduction of 15%, an adherence rate for each policy of 80%, and an average total cluster size of 1,000, we will require a total sample of 15,886 patients studied within 16 participating hospitals each with 12 periods (see Table 1).

Table 1: Total patients (N) and number of hospitals (N/ m̃) required to be randomized for each of two intervention groups to assure a sufficient power of 80% and a Type I error rate of 5% (2-sided) with 12 periods, a coefficient of variation of 0.63 for an assumed unequal cluster size,

~~an anticipated control event rate (p_1) of 15% for different combinations of average cluster size per cluster ($\tilde{m}$), intracluster correlation (ICC) and relative risk reduction (RRR).~~

RRR	Overall Cluster size ($\bar{m}$)	# periods	ICC	Total N (inflated)	Sample size for an individual patient RCT	# hospitals (N/$\bar{m}$)
15%	1000	12	0.01	11648	7409	12
		12	0.02	**15886**	**7409**	**16**
	~~800~~	~~12~~	~~0.01~~	~~10785~~	~~7409~~	~~14~~
		12	0.02	14161	7409	18
20%	1000	12	0.01	6402	4072	7
		12	0.02	8731	4072	9
	800	12	0.01	5928	4072	8
		12	0.02	7783	4072	10

7.6 Adaptation to original design based on results of interim analysis

The parameters which play a critical role for accuracy of the B-Free sample size estimation are the control event rate and variance in event rate across clusters and periods (i.e., ICC and IPC). To take emerging data into account, we undertook blinded sample size re-estimation (bSSR) without unblinding the treatment effect. This approach is considered effective to adjust the sample size to achieve the desired power at the end of trial without seriously inflating type I error. Specifically, we used data collected prior to interim analysis to estimate the uncertain parameters used in the original sample size calculation based on a proportion (50%) of the *a priori* required sample size. The steps we undertook can be summarized as follows: 1) calculate sample size; 2) collect a proportion of data; 3) estimate the parameters; 4) re-calculate sample size; 5) increase the number of periods if needed.

Modified sample size: Given 50% of the required sample size, the overall delirium rate was 16.2%, the ICC was 0.04, and the IPC was 0.25*ICC (i.e., 0.01). Taking into consideration of a treatment effect of 15% reduction on the delirium rate proposed at the design stage, we assumed a control rate of 17.5%. Furthermore, because of decreases in cardiac surgery case volume related to the COVID-19 pandemic, the average size of each cluster was 750. With current sample size (i.e., 20 clusters, 12 periods, 750 patients per cluster), the trial would be underpowered with 64% power to detect a 15% reduction. To re-estimate the sample size based observed parameters, we propose for 11 clusters to complete up to 6 additional crossover periods dependent on local feasibility. Based on the results of our adaptive analysis, our modified sample size includes 9 hospitals completing 18 periods, 2 hospitals completing 17 periods, and 9 hospitals completing 12 periods, with an overall average of 900 cardiac surgery patients per cluster and a control delirium rate of 17%, assuming an ICC of 0.06, IPC of 0.03, and type I error of 5%.

Table 1: Assumed, Observed, and Recalculated Sample Size Calculations

	**RRR**	**Average cluster size**	**Control delirium incidence**	**# Periods**	**ICC**	**IPC**	**Total n (projected)**	**# clusters**	**Alpha**	**Power**
**Assumed**	**15%**	**1000**	**0.15**	**12**	**0.02**	**0.01**	**16000**	**16**	**5%**	**0.8**
**Observed**	**-**	**750**	**0.17**	**12**	**0.06**	**0.03**	**15000**	**20**	**-**	**0.70**
**Recalculated**	**15%**	**1000**	**0.17**	**12**	**0.06**	**0.03**	**20000**	**20**	**5%**	**0.74[1]**
	**15%**	**900**	**0.17**	**15**	**0.06**	**0.03**	**18000**	**20**	**5%**	**0.81[2]**
	**15%**	**1250**	**0.17**	**18**	**0.06**	**0.03**	**22500**	**20**	**5%**	**0.88[3]**

[1]Reflects statistical power with pre-pandemic case volumes and observed ICC/IPC.

Supplemental Material

This supplement contains the following items:

1. Original and only statistical analysis plan

STATISTICAL ANALYSIS PLAN

Version 1.0
2023-08-03

TABLE OF CONTENTS

LIST OF ABBREVIATIONS 3
1. INTRODUCTION 4
2. STUDY OBJECTIVES 4
Primary Objective 4
Secondary Objectives 4
3. POPULATIONS TO BE ANALYZED 4
4. OUTCOMES 5
5. SAMPLE SIZE 5
6. STATISTICAL ANALYSES 6
7. SUBGROUP ANALYSIS 7
REFERENCES 9
APPROVAL 10

LIST OF ABBREVIATIONS

bSSR	blinded Sample Size Re-estimation
CAM-ICU	Confusion Assessment Method-ICU
CI	Confidence Interval
CNS	Central Nervous System
CPB	Cardiopulmonary Bypass
CVICU	Cardiovascular Intensive Care Unit
DSMB	Data Safety Monitoring Board
eCRF	Electronic Case Report Form
EMR	Electronic Medical Record
FDA	United States Food and Drug Administration
GABA	Gamma-aminobutyric Acid
GCP	Good Clinical Practice
ICC	Intra-cluster Correlation Coefficient
ICDSC	Intensive Care Delirium Screening Checklist
ICEMAN	Instrument for assessing the Credibility of Effect Modification Analyses
ICH	International Council for Harmonisation
ICU	Intensive Care Unit
IEC	Independent Ethics Committee
IPC	Interperiod Correlation Coefficient
IRB	Institutional Review Board
LOS	Length of Stay
PHRI	Population Health Research Institute
PRE	Government of Canada Panel on Research Ethics
REB	Research Ethics Board
SAP	Statistical Analysis Plan
SCCM	Society for Critical Care Medicine
TCPS	Tri-Council Policy Statement

1. INTRODUCTION

Benzodiazepine-free cardiac anesthesia for the reduction of postoperative delirium (B-Free; Protocol Final Version 5.01 dated 2022-10-17) is a multicenter, cluster-randomized, crossover trial to evaluate the impact of an institutional policy of limited benzodiazepine use during cardiac surgery, as compared to a policy of liberal benzodiazepine use during cardiac surgery, on the incidence of delirium during the postoperative intensive care unit (ICU) stay up to 72 hours.

This Statistical Analysis Plan (SAP) describes the statistical methods for the B-FREE Trial. It contains definitions of analysis sets, key derived variables, and provides a technical and detailed elaboration of the principal features of the planned analyses.

The SAP was finalized without knowledge of any emerging results by trial treatment group. The final version of the SAP was be signed off before database freeze.

The SAP was written fully blinded to the treatment allocation with the intent to fully preserve the integrity of the statistical analyses that have been outlined in the final clinical trial protocol.

2. STUDY OBJECTIVES

Primary Objective

The primary objective of the B-Free trial is to evaluate the impact of an institutional policy of limited benzodiazepine use during cardiac surgery, as compared to a policy of liberal benzodiazepine use during cardiac surgery, on the incidence of delirium up to 72 hours after cardiac surgery.

Secondary Objectives

The secondary objectives of the B-Free trial are to evaluate the impact of these policies on:

1. ICU length of stay (LOS)
2. Hospital LOS
3. All cause in-hospital mortality

3. POPULATIONS TO BE ANALYZED

The intention-to-treat population will include all adult (≥ 18 years) patients who underwent cardiac surgery during active trial periods at each included cluster.

4. OUTCOMES

Primary Outcome

The primary study outcome is delirium up to 72 hours after cardiac surgery.

Secondary Outcomes

1. Intensive Care Unit (ICU) length-of-stay (LOS)
2. Hospital LOS
3. In-hospital mortality

Outcome Definitions

1. Delirium: Delirium was be measured using either the Confusion Assessment Method-ICU (CAM-ICU)[1] or the Intensive Care Delirium Screening Checklist (ICDSC)[2] in routine clinical care. We will consider patients to have delirium if they fulfill the criteria for delirium (binary) on either of the two scales up to 72 hours after cardiac surgery.
2. ICU LOS: This is defined as the number of hours in the cardiac surgical ICU following index cardiac surgery until ICU discharge.
3. Hospital LOS: This is defined as the number of days from index cardiac surgery until hospital discharge.
4. In-hospital mortality: This is defined as death from any cause after the start of the index cardiac surgical procedure and until hospital discharge.

All secondary outcomes (i.e., ICU LOS, Hospital LOS, and In-hospital mortality) will be censored on the date of SAP finalization. For the outcome of in-hospital mortality, patients will be considered to be alive at the time of censoring if they remain alive in hospital.

5. SAMPLE SIZE

Based on our initial sample size calculation, we required 16 hospitals with an overall average annual case volume of 1000 patients to complete 12, 4-week crossover periods to achieve statistical power >80%. This number of clusters and patients would allow for the detection of a relative risk reduction of 15% based on a control delirium rate of 15%, and our assumptions of an intracluster correlation coefficient (ICC) of 0.02, interperiod correlation (IPC) equal to one half of the ICC (i.e., 0.01), and type I error of 5%.

Given that the sample size depends on the assumption of ICC and the control delirium rate, we performed blinded sample size re-estimation (bSSR) without unblinding the treatment effect at the interim analysis when 70% of patients had been enrolled. This approach is considered effective to adjust the sample size to achieve the desired power at the end of trial with limited effects on type I error.[7,8] We conducted a simulation to

evaluate the performance. Our simulation results demonstrated an improvement in empirical power from 70% to 81%, and a type I error of 5% within the reasonable range.

Thus, our modified sample size based on the adaptive analysis includes 9 hospitals completing 18 periods, 2 hospitals completing 17 periods, and 9 hospitals completing 12 periods, with an overall average of 900 cardiac surgery patients contributed per cluster. This will give 81% power to detect a relative reduction of 15% based on a control delirium rate of 17%, with an anticipated higher ICC of 0.06, IPC of 0.03, and type I error of 5%.

Table 1: Assumed, Observed, and Recalculated Sample Size Calculations

	RRR	Average cluster size	Control delirium incidence	# Periods	ICC	IPC	Total n (projected)	# clusters	Alpha	Power
Assumed	15%	1000	0.15	12	0.02	0.01	16000	16	5%	0.8
Observed	-	750	0.17	12	0.06	0.03	15000	20	-	0.70
Recalculated	15%	1000	0.17	12	0.06	0.03	20000	20	5%	0.74[1]
	15%	900	0.17	15[2]	0.06	0.03	18000	20	5%	0.81
	15%	1250	0.17	18	0.06	0.03	22500	20	5%	0.88[3]

[1]Reflects statistical power with pre-pandemic case volumes and observed ICC/IPC.
[2]Proposed adaptation. Number of crossover periods reflects average across all clusters (i.e., 18 in 9 sites, 17 in 2 sites, 12 in 9 sites).
[3]Statistical power using observed ICC/IPC if all sites completed 18 periods

6. STATISTICAL ANALYSES

Main analysis: The primary analyses will be based on the intention-to-treat principle, i.e., participants will be analyzed according to the allocated policy in use when they underwent surgery, regardless of whether they were managed according to the policy. Standard methods will be used to report tabular and graphical summaries as appropriate for continuous and categorical variables. Summaries of continuous variables will include the number of subjects (N), mean (standard deviation), and median (25th and 75th percentiles). Frequency distributions (N and %) will be reported for categorical data. We will report the standardized difference between treatment allocations for baseline characteristics.

All analyses will take place at the individual-patient level but will account for clustering. Analyses will be carried out comparing event rates in patients managed during the restricted compared to the liberal benzodiazepine policy periods. Primary and secondary outcomes will be compared between treatment allocation using a logistic mixed model for binary outcomes and a mixed negative binomial model for non-normal continuous outcomes, including a term for period, and using random effects to account for within-period intracluster correlation and an exponential decay in the strength of the correlation over time.[11] In all models we will adjust for the following baseline covariates: age (years), sex, urgency of surgery (emergency vs elective), history of heavy alcohol consumption, and history of home benzodiazepine use. We will report odds ratios and 95% confidence intervals for binary outcomes and mean difference and 95% CI for non-normal continuous outcomes. We will claim statistical significance for treatment

effectiveness if the p value is less than 0.05 for the primary outcome. We will use SAS 9.4 for UNIX (SAS Institute Inc., Cary, North Carolina, USA) or other validated software for all analyses.

7. SUBGROUP ANALYSIS

The following patient-level subgroups of interest will be evaluated: age in years (<50, 50 to ≤60, 60 to ≤70, 70 to ≤80, >80), and patients with home benzodiazepine use or alcohol abuse together and separately.

Subgroup analyses will be conducted using tests for interactions in hierarchical mixed models for the primary outcome. We will use the Instrument for assessing the Credibility of Effect Modification Analyses (ICEMAN) criteria to assess the credibility of each subgroup.[12] Table 2 presents *a priori* hypotheses regarding direction of effect. We will consider subgroups to be significant if they are assessed to have moderate or high credibility.

Table 2: Prespecified subgroups and associated hypotheses to explain heterogeneity in effect of intraoperative benzodiazepine policy on postoperative delirium

Subgroup	Hypothesized effect of intraoperative benzodiazepine policy on delirium
Age (years)*	Restricted intraoperative benzodiazepines will be associated with a greater relative risk reduction in older compared to younger patients.
Patients with home benzodiazepine use and/or alcohol abuse	Restricted intraoperative benzodiazepine administration will be associated with a smaller relative risk reduction in patients with a history of home benzodiazepine and/or alcohol abuse compared to patients without a history of home benzodiazepine and/or alcohol abuse.

*grouped in decades as follows: <50, 50 to ≤60, 60 to ≤70, 70 to ≤80, >80

REFERENCES

1. Inouye SK, van Dyck CH, Alessi CA, Balkin S, Siegal AP, Horwitz RI. Clarifying confusion: the confusion assessment method. A new method for detection of delirium. *Annals of internal medicine*. Dec 15 1990;113(12):941-8.
2. Roberts B, Rickard CM, Rajbhandari D, et al. Multicentre study of delirium in ICU patients using a simple screening tool. *Aust Crit Care*. Feb 2005;18(1):6, 8-9, 11-4 passim.
3. Gulliford MC, Adams G, Ukoumunne OC, Latinovic R, Chinn S, Campbell MJ. Intraclass correlation coefficient and outcome prevalence are associated in clustered binary data. *J Clin Epidemiol*. Mar 2005;58(3):246-51. doi:10.1016/j.jclinepi.2004.08.012
4. Thompson SG, Pyke SD, Hardy RJ. The design and analysis of paired cluster randomized trials: an application of meta-analysis techniques. *Stat Med*. Sep 30 1997;16(18):2063-79.
5. Connolly SJ, Philippon F, Longtin Y, et al. Randomized cluster crossover trials for reliable, efficient, comparative effectiveness testing: design of the Prevention of Arrhythmia Device Infection Trial (PADIT). *The Canadian journal of cardiology*. Jun 2013;29(6):652-8. doi:10.1016/j.cjca.2013.01.020
6. Giraudeau B, Ravaud P, Donner A. Sample size calculation for cluster randomized cross-over trials. *Stat Med*. Nov 29 2008;27(27):5578-85. doi:10.1002/sim.3383
7. Wittes J, Schabenberger O, Zucker D, Brittain E, Proschan M. Internal pilot studies I: type I error rate of the naive t-test. *Stat Med*. Dec 30 1999;18(24):3481-91. doi:10.1002/(sici)1097-0258(19991230)18:24<3481::aid-sim301>3.0.co;2-c
8. Lake S, Kammann E, Klar N, Betensky R. Sample size re-estimation in cluster randomization trials. *Stat Med*. May 30 2002;21(10):1337-50. doi:10.1002/sim.1121
9. Morgan KE, Forbes AB, Keogh RH, Jairath V, Kahan BC. Choosing appropriate analysis methods for cluster randomised cross-over trials with a binary outcome. *Stat Med*. Jan 30 2017;36(2):318-333. doi:10.1002/sim.7137
10. Hemming K, Taljaard M, Forbes A. Modeling clustering and treatment effect heterogeneity in parallel and stepped-wedge cluster randomized trials. *Stat Med*. Mar 15 2018;37(6):883-898. doi:10.1002/sim.7553
11. Kasza J, Forbes AB. Inference for the treatment effect in multiple-period cluster randomised trials when random effect correlation structure is misspecified. *Stat Methods Med Res*. Oct-Nov 2019;28(10-11):3112-3122. doi:10.1177/0962280218797151
12. Schandelmaier S, Briel M, Varadhan R, et al. Development of the Instrument to assess the Credibility of Effect Modification Analyses (ICEMAN) in randomized controlled trials and meta-analyses. *CMAJ : Canadian Medical Association journal = journal de l'Association medicale canadienne*. Aug 10 2020;192(32):E901-E906. doi:10.1503/cmaj.200077

APPROVAL

Version #	**1.0**
Version Date	August 3, 2023

By signing the below, I designate my approval of the above-named version of the B-Free Trial Statistical Analysis Plan on behalf of all investigators.

**Name**	**Jessica Spence**
**Role**	**Principal Investigator**
**Signature**	DocuSigned by:<br>Jessica Spence<br>Signer Name: Jessica Spence<br>Signing Reason: I approve this document<br>Signing Time: 2023-08-04 | 3:34:23 PM EDT<br>E06CC11CE8984853983B0EAD7101A574
**Date**<br>(yyyy/mm/dd)	2023-08-04 | 3:34:28 PM EDT

By signing the below, I designate my approval of the above-named version of the B-Free Trial Statistical Analysis Plan on behalf of PHRI Statistics.

**Name**	**Shun Fu Lee**
**Role**	**Blinded Study Statistician**
**Signature**	DocuSigned by:<br>Shun-Fu Lee<br>Signer Name: Shun Fu Lee<br>Signing Reason: I approve this document<br>Signing Time: 2023-08-03 | 10:10:28 AM EDT<br>F16F9B626DEC4F2882A3B8C7434204D4
**Date**<br>(yyyy/mm/dd)	2023-08-03 | 10:10:32 AM EDT
