# Supplementary material for: Benzodiazepine-Free Cardiac Anesthesia for Reduction of Postoperative Delirium: A Cluster Randomized Crossover Trial
Source: JAMA Surg. 2025 Jan 29;160(3):286–94. doi: 10.1001/jamasurg.2024.6602 (PMC11780505; doi:10.1001/jamasurg.2024.6602)
Supplement: Supplement 2. — eAppendix. Study groups eMethods eTable 1. Assumed, Observed, and Recalculated Sample Size Calculations eTable 2. Conversion of opioids to Fentanyl equivalents eTable 3. Conversion of benzodiazepines to Midazolam equivalents eFigure 1. Randomization schedule for participating clusters eTable 4. Delirium assessments by treatment allocation by day eTable 5. Cluster level Characteristics eTable 6. Detailed surgical characteristics eFigure 2. Results for the incidence of delirium up to 72 hours after cardiac surgery by center eTable 7. Results for the incidence of delirium up to 72 hours after cardiac surgery by center reporting which scale used eFigure 3. Number of assessments documenting the presence of delirium per 12 hours after cardiac surgery by intervention allocation eTable 8. Sensitivity analysis excluding patients who were not assessed for delirium [file jamasurg-e246602-s002.pdf]

Supplemental Online Content

Spence J, Devereaux PJ, Shun-Fu Lee SF, et al; B-Free Investigators; Canadian Perioperative Anesthesia Clinical Trials Group. Benzodiazepine-free cardiac anesthesia for reduction of postoperative delirium: a cluster randomized clinical trial. *JAMA Surg*. Published online January 29, 2025. doi:10.1001/jamasurg.2024.6602

eAppendix. Study groups

eMethods

eTable 1. Assumed, Observed, and Recalculated Sample Size Calculations

eTable 2. Conversion of opioids to Fentanyl equivalents

eTable 3. Conversion of benzodiazepines to Midazolam equivalents

eFigure 1. Randomization schedule for participating clusters

eTable 4. Delirium assessments by treatment allocation by day

eTable 5. Cluster level Characteristics

eTable 6. Detailed surgical characteristics

eFigure 2. Results for the incidence of delirium up to 72 hours after cardiac surgery by center

eTable 7. Results for the incidence of delirium up to 72 hours after cardiac surgery by center reporting which scale used

eFigure 3. Number of assessments documenting the presence of delirium per 12 hours after cardiac surgery by intervention allocation

eTable 8. Sensitivity analysis excluding patients who were not assessed for delirium

This supplemental material has been provided by the authors to give readers additional information about their work.

© 2025 Spence J et al. *JAMA Surgery*.

eAppendix. Study groups

Steering Committee
Jessica Spence, Emilie Belley-Côté, Richard P Whitlock, Eric Jacobsohn, Frédérick D'Aragon, Michael S Avidan, Christopher Beaver, PJ Devereaux, Shelley Kloppenburg, Shun-Fu Lee, Simon JW Oczkowski, Stuart Connolly (deceased)

Data and Safety Monitor (DSM)
George Wyse (deceased), John Eikelboom

Project Office Staff
Emily Di Sante, Erin DeBorba, Tara Robinson, Gladys Marfo, Jessica Vincent

Study Statisticians and Programmers
Kumar Balasubramanian, Shun Fu Lee, Chinthanie F. Ramasundarahettige, Peter Koh

Patient Partners
Christopher Beaver, Shelley Kloppenburg

Participating Centres
CANADA (18) – *Hamilton General Hospital- Hamilton Health Sciences*: Emilie Belley-Côté, Richard P Whitlock, William F. McIntyre, Andre Lamy, Courtney Mullen, Leah Hayward, Matthew Ryan McFarling, Kim Botsford, Summer Syed, Maia Shen, Renée Fournier; *St. Boniface General Hospital:* Eric Jacobsohn, Morvarid Kavosh, Kate MacKenzie, Marita Monterola, Linda Girling; *Centre Hospitalier Universitaire de Sherbrooke*: Frédérick D'Aragon, Mathilde St-Pierre, Michel-Antoine Perreault, Étienne de Medicis, Jonathan Gaulin; *Jewish General Hospital:* Matthew J Cameron; *St. Paul's Hospital:* Raja Ramaswamy Rajamohan, Nicola Edward, Ron Ree, Iris Yao, Clement Chui, Michelle Biferie, Connie Game; *Vancouver General Hospital:* Rael Klein, Juliet Ann Atherstone, Darren Mullane, Juliet Atherstone; *Royal Columbian Hospital:* Michael W.Y. Law, Michelle Mozel, Mikaela Barton, Ashlee Hughes, Aiman Hasnat; *Kingston General Hospital- Kingston Health Sciences Centre:* Tarit Saha, Ramiro Arellano, Robert Tanzola, Debbie DuMerton, Michael Cummings; *Toronto General Hospital:* George Djaiani; *St. Michael's Hospital:* C. David Mazer, Ahmad Alli, Samson Moses, Niloufar Siadati-Fini, Kyle Chin, Greg Hare; *Mazankowski Heart Institute:* Wing Lam, Maliha Muneer; *Queen Elizabeth II Health Sciences Centre*: Edmund Tan, Braden J Dulong, Izabela M Panek, Kaela H Fraser, Flynn A Bonazza, Sharon E Amey; *University of Saskatchewan*: D Ryan Pikaluk, Shelley Roulston; *Montreal Heart Institute*: Nicolas Rousseau-Saine, Alain Deschamps, Sophie Robichaud, Marco Julien, Antoine Rochon, Marie-Eve Chamberland, Meggie Raymond, Jennifer Cogan, Georges Desjardins, Jean-Sébastien Lebon, Christian Ayoub, Pierre Couture, Athanase Courbe, Maria Rosal Martins, André Y. Denault, An Ni Wu, Kristofer Beggs, Stéphanie Jarry, Sarah Bendaoud, Ester Cisneros-Aguilera, Lana Agoian, Melissa Laurendeau, Elaine Duval, Anya Chabane, Martine Lacroix; *University Hospital - London Health Sciences Centre*: Raffael Pereira Cezar Zamper, Liam J Kennedy, Robert Christopher Mayer; *Royal*

© 2025 Spence J et al. *JAMA Surgery.*

University Hospital: Peter Hedlin, Michelle Clunie, Azeez Akinlade; *Institut universitaire de cardiologie et de pneumologie de Québec - Université Laval* : Etienne J Couture, Hugo Tremblay, Nathalie Gagné, Annie Bergeron, François Laforge, Audrey Grenier, Valérie Morin, Sandrine Bellavance, Valérie Lafrenière-Bessi, Béatrice Martin, Joanie Lachance, Olivier Audet, Léa Vachon-Zicat, Yasmine Babaki, Élizabeth St-Onge, Nelson Lavoie, Mathilde Bisson, Marie-Ève Charest; *Sunnybrook Health Sciences*: Stephen Choi, Angela Jerath, Lilia Kaustov, Andrew Fleet, Sophia Wong, Elizabeth Lappin, Saba Shaheen;

USA (2) – *Washington University School of Medicine:* Michael S Avidan, Mohammad Helwani, Thaddeus Budelier, Arianna Montes de Oca, Alex Kronzer*; Weill Cornell Medicine:* Kane O Pryor, Meghann M Fitzgerald, Natalia I Girardi, Leonard N Girardi, Michele L Steinkamp, Lisbeth A Evered, Hannah R Leibowitz, Dylan R Bitensky, Lisbeth Evered

The Canadian Perioperative Anesthesia Clinical Trials Group (PACT):
Steering Committee - Alexis Turgeon, André Denault, Daniel McIsaac, C. David Mazer, Jessica Spence, Kathryn Sparrow, Manoj Lalu, Stuart McCluskey, Richard Hall (Founding Director), W. Scott Beattie.

© 2025 Spence J et al. *JAMA Surgery*.

eMethods

Intervention Arm Policies

The "Restricted Benzodiazepine Policy" arm	The "Liberal Benzodiazepine Policy" arm
No routine use of any intraoperative benzodiazepines<br><br>Accepted administration of benzodiazepines when deemed mandatory by the attending anaesthetist (e.g., seizure, alcohol withdrawal, benzodiazepine dependence, history of awareness during anesthesia, hemodynamic instability)	Intraoperative administration of the equivalent of at least 0.03mg/kg midazolam ideal body weight (60 kg in women, 70 kg in men) equivalent to all patients undergoing cardiac surgery<br><br>Any benzodiazepine may be given intraoperatively<br><br>Accepted avoidance of benzodiazepines in patients who have contraindications to the administration of these medications (e.g., documented allergy, previous adverse reaction)

© 2025 Spence J et al. *JAMA Surgery*.

Outcome Definitions

Delirium within 72 hours of cardiac surgery: Delirium (dichotomous; yes/no) diagnosed using either the Confusion Assessment Method-ICU (CAM-ICU) or the Intensive Care Delirium Screening Checklist (ICDSC).

ICU LOS: This is defined as the number of hours in the cardiac surgical ICU following index cardiac surgery until ICU discharge.

Hospital LOS: This is defined as the number of days from index cardiac surgery until hospital discharge.

In-hospital mortality: This is defined as death from any cause after the index cardiac surgical procedure and until hospital discharge.

© 2025 Spence J et al. *JAMA Surgery*.

Detailed description of trial adaptation

Because of concerns about the impact of the COVID-19 pandemic on cardiac case volumes and the uncertainty of the assumptions used in our original sample size estimate, we used aggregate data to validate our original sample size estimation, with a view to extend the duration of the trial if needed to maintain statistical power at 80%. Based on data from 11,222 patients, we confirmed an overall incidence of delirium of 16.6%, greater than that assumed (13.9% overall [assuming an effect of intervention]; 15% in control arm). We also observed a projected average cluster size of 750, lower than originally anticipated based on pre-pandemic case volumes. As this was a cluster crossover randomized trial, the intra-cluster correlation (ICC) was the critical parameter for sample size estimation. By estimating this value with greater precision and considering it in light of the decrease in surgical volumes, we hoped to avoid an underpowered study. We performed sample size re-estimation without unblinding the treatment effect. This approach is considered effective to adjust the sample size to achieve the desired power at the end of trial with minimal inflation in type I error. [1,2] Specifically, we used complete data collected up to May 9, 2022 to estimate the uncertain parameters used in our sample size calculation based on a proportion (70%) of the *a priori* required sample size. The steps in our adaptive analysis can be summarized as follows: 1) calculate sample size; 2) collect a proportion of data; 3) estimate the ICC from available data; 4) re-calculate the sample size based on the observed ICC; 5) increase the number of cluster-periods if needed.

The data for our adaptive analysis were derived from 20 clusters with an average of 9.5 periods per cluster. In this data set, we observed an ICC = 0.06 with a bootstrap 95% confidence interval (CI) of (0.05, 0.07). Based on the observed ICC, our projected sample size (i.e., 20 clusters completing 12 periods, average cluster size 750) would be underpowered, with 70% power to detect a 15% relative risk reduction in the incidence of postoperative delirium.

There are no published data about the performance characteristics of sample size re-estimation for a cluster crossover trial based on a proportion of collected data. We thus conducted a simulation to assess the type I error and power associated with this internal pilot adaptive design. Simulation data were generated using a generalized logistic mixed model with both cluster and cluster-period as random-effects. [3,4] Based on our observed event rate, we assumed a control incidence of delirium of 17%, an average cluster size of 900 participants (which we sought to achieve through the addition of further crossover periods), and a total of 12,

15, and 18 periods per cluster (assuming all clusters completed all periods). The simulation had two steps. First, we generated data based on an ICC of 0.06, 20 clusters, an average cluster size of 900, and 12 crossover periods. We then estimated ICC using 70% of generated data and increased the number of periods (and thus average cluster size) if required to maintain power ≥80%. Second, we regenerated a dataset that included the number of cluster-periods required according to the estimated ICC. We repeated the process 1000 times to estimate type I error and power. The 95% confidence interval for nominal alpha level of 5% based on a simulation of 1000 runs was set to be 3.6% - 6.4%, corresponding to statistical tests with type I error rates less than 3.6% considered overly conservative and greater than 6.4% overly liberal. Our simulation results demonstrated an improvement in empirical power from 70% to 81%, and a type I error of 5% within the reasonable range. Based on observed case volumes, we determined that this could be achieved by having nine clusters complete an additional six periods, and two clusters complete an additional five periods (i.e., an average of 15 periods across 20 clusters). Sites were selected to take part in the trial extension according to local feasibility related to human resources.

i) Modified sample size: Based on the results of our adaptive analysis, our modified sample size includes nine hospitals completing 12 periods, nine hospitals completing 18 periods, and two hospitals completing 17 periods, with an overall average of 900 cardiac surgery patients contributed per cluster. This gave 81% power to detect a relative reduction of 15% based on a control delirium rate of 17%, assuming an ICC of 0.06, IPC of 0.03, and type I error of 5% (eTable1).

eTable 1: Assumed, Observed, and Recalculated Sample Size Calculations

	RRR	Average cluster size	Control delirium incidence	# Periods	ICC	IPC	Total n (projected)	# clusters	Alpha	Power
Assumed	15%	1000	0.15	12	0.02	0.01	16000	16	5%	0.8
Observed	-	750	0.7	12	0.06	0.03	15000	20	-	0.70
Recalculated	15%	1000	0.17	12	0.06	0.03	20000	20	5%	0.74[1]
	15%	900	0.17	15	0.06	0.03	1800	20	5%	0.81[2]
	15%	1250	0.17	18	0.06	0.03	22500	20	5%	0.88[3]

[1]Reflects statistical power with pre-pandemic case volumes and observed ICC/IPC.
[2]Proposed adaptation. Number of crossover periods reflects average across all clusters (i.e., 18 in 9 sites, 17 in 2 sites, 12 in 9 sites).
[3]Statistical power using observed ICC/IPC if all sites completed 18 periods

© 2025 Spence J et al. *JAMA Surgery*.

References

1. Wittes J, Schabenberger O, Zucker D, Brittain E, Proschan M. Internal pilot studies I: type I error rate of the naive t-test. *Stat Med* 1999; **18**(24): 3481-91.
2. Lake S, Kammann E, Klar N, Betensky R. Sample size re-estimation in cluster randomization trials. *Stat Med* 2002; **21**(10): 1337-50.
3. Morgan KE, Forbes AB, Keogh RH, Jairath V, Kahan BC. Choosing appropriate analysis methods for cluster randomised cross-over trials with a binary outcome. *Stat Med* 2017; **36**(2): 318-33.
4. Hemming K, Taljaard M, Forbes A. Modeling clustering and treatment effect heterogeneity in parallel and stepped-wedge cluster randomized trials. *Stat Med* 2018; **37**(6): 883-98.

© 2025 Spence J et al. *JAMA Surgery*.

Conversion factors used for opioids and benzodiazepines

eTable 2: Conversion of opioids to Fentanyl equivalents

Drug Dose	Equivalents
Sufentanil dose (mcg) X 10	Fentanyl equivalents (in mcg)
Remifentanil dose (mcg) X 1	Fentanyl equivalents (in mcg)
Hydromorphone dose (mg) X 70	Fentanyl equivalents (in mcg)
Morphine dose (mg) X 10	Fentanyl equivalents (in mcg)
Total opioid dose	Sum of all opioids in Fentanyl equivalents (mcg)

eTable 3: Conversion of benzodiazepines to Midazolam equivalents

Drug Dose	Equivalents
Clonazepam 1 mg	Midazolam 4 mg
Lorazepam 1 mg	Midazolam 2 mg
Diazepam 1 mg	Midazolam 0.25 mg
Total benzodiazepine dose	Sum of all benzodiazepines in Midazolam equivalents (mg)

© 2025 Spence J et al. *JAMA Surgery*.

eFigure 1: Randomization schedule for participating clusters

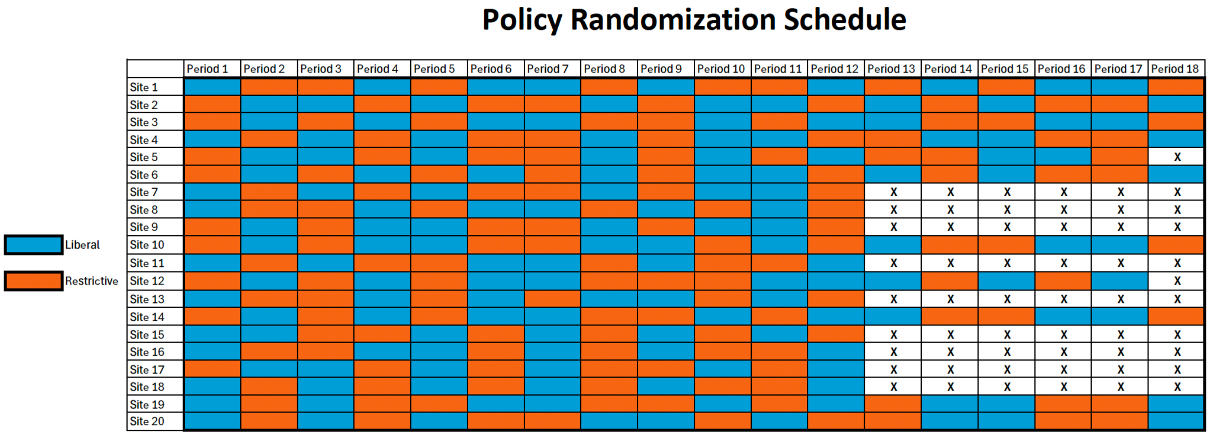

© 2025 Spence J et al. *JAMA Surgery*.

eTable 4: Delirium assessments by treatment allocation by day

	N	Overall	N	Restrictive benzodiazepine policy	N	Liberal benzodiazepine policy
**Randomized**	19768	19768 (100)	9827	9827 (100)	9941	9941 (100)
**Delirium assessed – no. (%)**	19768	18901 (95.6)	9827	9380 (95.5)	9941	9521 (95.8)
**Delirium not assessed – no. (%)**	19768	867 (4.4)	9827	447 (4.6)	9941	420 (4.2)
**Number of times delirium assessed within 72-hrs of surgery – mean (SD)**	18901	5.8 ±4.0	9380	5.8 ±4.0	9521	5.7 ±3.9
**Number of times delirium assessed using CAM-ICU within 72-hrs of surgery – mean (SD)**	18901	3.2 ±4.4	9380	3.2 ±4.5	9521	3.2 ±4.4
**Number of times delirium assessed using ICDSC within 72-hrs of surgery – mean (SD)**	18901	2.5 ±3.3	9380	2.5 ±3.3	9521	2.5 ±3.2
**Number of times delirium assessed within 72-hrs of surgery – median (IQR)**	18901	5 (3-7)	9380	5 (3-7)	9521	5 (3-7)
**Number of times delirium assessed using CAM-ICU within 72-hrs of surgery – median (IQR)**	18901	1 (0-5)	9380	1 (0-5)	9521	1 (0-5)
**Number of times delirium assessed using ICDSC within 72-hrs of surgery – median (IQR)**	18901	1 (0-4)	9380	1 (0-4)	9521	1 (0-4)
**Delirium assessed within 24-hours of surgery – no. (%)**	18901	16905 (89.4)	9380	8396 (89.5)	9521	8509 (89.4)
**Delirium present within 24-hours of surgery – no. (%)**	16905	1559 (9.2)	8396	742 (8.8)	8509	817 (9.6)
**Delirium assessed within 24 to 48 hours of surgery – no. (%)**	18901	13858 (73.3)	9380	6897 (73.5)	9521	6961 (73.1)
**Delirium present within 24 to 48 hours of surgery – no. (%)**	13858	1357 (9.8)	6897	632 (9.2)	6961	725 (10.4)
**Delirium assessed within 48 to 72 hours of surgery – no. (%)**	18901	11781 (62.3)	9380	5871 (62.6)	9521	5910 (62.1)
**Delirium present within 48 to 72 hours of surgery – no. (%)**	11781	1183 (10.0)	5871	573 (9.8)	5910	610 (10.3)

© 2025 Spence J et al. *JAMA Surgery*.

eTable 5: Cluster level Characteristics

Cluster characteristic	Median (IQR) or N (%)
Total cluster size - median (IQR)	972 (595-1199)
Cluster-period size - median (IQR)	63 (39-80)
Cluster size - no. (%)	
≤500	4 (20.0)
>500 to ≤1000	7 (35.0)
>1000	9 (45.0)
Year of trial initiation - no. (%)	
2019	2 (10.0)
2020	16 (80.0)
2021	2 (10.0)

Abbreviations: IQR: interquartile range

© 2025 Spence J et al. *JAMA Surgery*.

eTable 6: Detailed surgical characteristics

Characteristic	Restricted benzodiazepine policy (N=9827)	Liberal benzodiazepine policy (N=9941)	Standardized difference
Surgical procedure– no. (%)			
Single valve repair/replacement	1725 (17.6)	1734 (17.4)	<0.01
Double valve repair/replacement	347 (3.5)	356 (3.6)	<0.01
Isolated CABG	4765 (48.5)	4896 (49.3)	0.02
CABG with single valve repair/replacement	887 (9.0)	898 (9.0)	<0.01
CABG with double valve repair/replacement	97 (1.0)	86 (0.9)	0.01
CABG with ascending aorta replacement	58 (0.6)	73 (0.7)	0.02
Isolated aorta surgery	215 (2.2)	262 (2.6)	0.03
Aorta surgery, with aortic valve repair/replacement	398 (4.05)	371 (3.7)	0.02
Aorta surgery, with other procedure	188 (1.9)	179 (1.8)	<0.01
Cardiac transplant	41 (0.4)	68 (0.7)	0.04
Ventricular assist device insertion	66 (0.7)	64 (0.6)	<0.01
Isolated pericardial procedure	17 (0.2)	18 (0.2)	<0.01
Other cardiac surgery	1023 (10.4)	936 (9.4)	0.03
Use of hypothermic circulatory arrest – no. (%)	98 (1.0)	114 (1.1)	0.05

Abbreviations: CABG: Coronary artery bypass grafting

© 2025 Spence J et al. *JAMA Surgery*.

eFigure 2: Results for the incidence of delirium up to 72 hours after cardiac surgery by center

	Restrictive Benzodiazepine Event/Total(%)	Liberal Benzodiazepine Event/Total(%)	Unadjusted Odds Ratio (95% CI)	Adjusted Odds Ratio* (95% CI)	Interaction P value
Overall	1373/ 9827 (13.97)	1485/ 9941 (14.94)	0.92 (0.84 - 1.01)	0.92 (0.84 - 1.01)	
Site 1	168/ 858 (19.58)	173/ 845 (20.47)	0.93 (0.44 - 1.95)	0.95 (0.45 - 2.02)	0.625
Site 2	121/ 512 (23.63)	134/ 513 (26.12)	0.88 (0.66 - 1.17)	0.89 (0.66 - 1.18)	
Site 3	46/ 266 (17.29)	48/ 264 (18.18)	0.94 (0.56 - 1.59)	0.90 (0.53 - 1.52)	
Site 4	81/ 341 (23.75)	81/ 318 (25.47)	0.91 (0.61 - 1.36)	0.89 (0.59 - 1.32)	
Site 5	63/ 574 (10.98)	62/ 518 (11.97)	0.91 (0.62 - 1.32)	0.91 (0.63 - 1.33)	
Site 6	78/ 432 (18.06)	77/ 469 (16.42)	1.08 (0.63 - 1.85)	1.08 (0.61 - 1.90)	
Site 7	35/ 398 (8.79)	34/ 385 (8.83)	1.00 (0.61 - 1.63)	0.97 (0.59 - 1.60)	
Site 8	7/ 205 (3.41)	15/ 194 (7.73)	0.42 (0.17 - 1.06)	***	
Site 9	37/ 562 (6.58)	54/ 581 (9.29)	0.70 (0.42 - 1.16)	***	
Site 10	95/ 699 (13.59)	98/ 684 (14.33)	0.95 (0.62 - 1.45)	0.96 (0.61 - 1.52)	
Site 11	45/ 472 (9.53)	42/ 486 (8.64)	1.11 (0.72 - 1.73)	1.09 (0.70 - 1.71)	
Site 13	57/ 480 (11.88)	93/ 541 (17.19)	0.65 (0.45 - 0.93)	0.64 (0.44 - 0.91)	
Site 14	3/ 149 (2.01)	10/ 157 (6.37)	0.31 (0.07 - 1.45)	0.31 (0.06 - 1.60)	
Site 16	117/ 1191 (9.82)	106/ 1154 (9.19)	1.11 (0.75 - 1.62)	1.12 (0.77 - 1.63)	
Site 17	28/ 405 (6.91)	28/ 471 (5.94)	0.99 (0.32 - 3.11)	***	
Site 18	15/ 122 (12.30)	12/ 118 (10.17)	1.24 (0.41 - 3.72)	***	
Site 19	64/ 856 (7.48)	69/ 860 (8.02)	0.93 (0.59 - 1.47)	0.94 (0.59 - 1.48)	
Site 21	19/ 217 (8.76)	25/ 230 (10.87)	0.79 (0.42 - 1.47)	0.79 (0.42 - 1.50)	
Site 22	214/ 602 (35.55)	243/ 653 (37.21)	0.93 (0.71 - 1.22)	***	
Site 23	80/ 486 (16.46)	81/ 500 (16.20)	1.00 (0.64 - 1.57)	0.95 (0.60 - 1.49)	

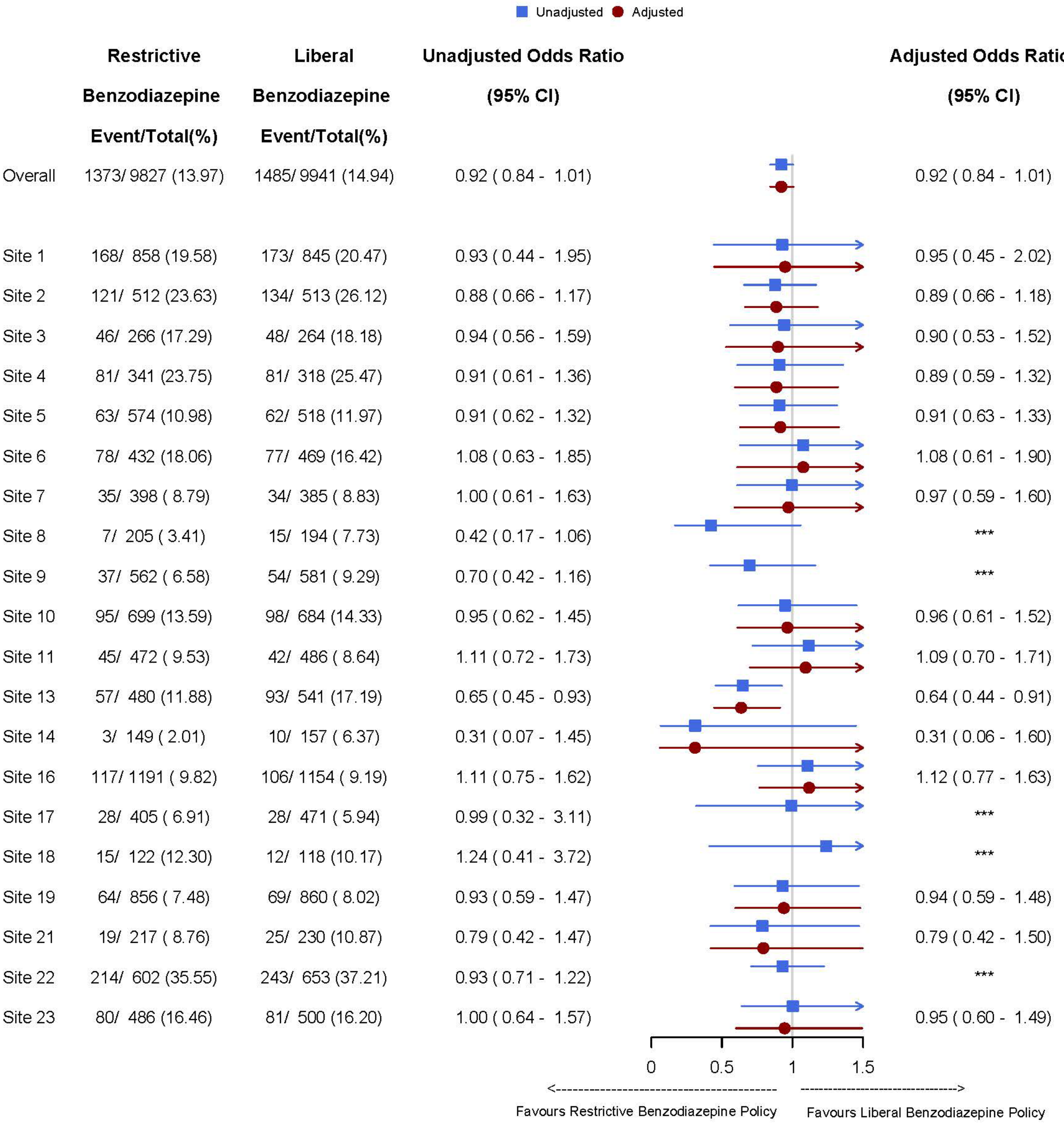

© 2025 Spence J et al. *JAMA Surgery*.

eTable 5: Results for the incidence of delirium up to 72 hours after cardiac surgery by center reporting which scale used

	Delirium scale used	Overall		Restrictive benzodiazepine policy		Liberal benzodiazepine policy		Unadjusted odds ratio (95% CI)	P for interaction
	ICDSC or CAM	N	n (%)	N	N (%)	N	N (%)		
Overall		19768	2858 (14.5)	9827	1373 (14.0)	9941	1485 (14.9)	0.92 (0.84 - 1.01)	0.63
Site 1	CAM	1703	341 (20.0)	858	168 (19.6)	845	173 (20.5)	0.93 (0.44 - 1.95)	
Site 2	CAM	1025	255 (24.9)	512	121 (23.6)	513	134 (26.1)	0.88 (0.66 - 1.17)	
Site 3	ICDSC	530	94 (17.7)	266	46 (17.3)	264	48 (18.2)	0.94 (0.56 - 1.59)	
Site 4	CAM	659	162 (24.6)	341	81 (23.8)	318	81 (25.5)	0.91 (0.61 - 1.36)	
Site 5	ICDSC	1092	125 (11.5)	574	63 (11.0)	518	62 (12.0)	0.91 (0.62 - 1.32)	
Site 6	ICDSC	901	155 (17.2)	432	78 (18.1)	469	77 (16.4)	1.08 (0.63 - 1.85)	
Site 7	CAM	783	69 (8.8)	398	35 (8.8)	385	34 (8.8)	1.00 (0.61 - 1.63)	
Site 8	CAM	399	22 (5.5)	205	7 (3.4)	194	15 (7.7)	0.42 (0.17 - 1.06)	
Site 9	CAM	1143	91 (8.0)	562	37 (6.6)	581	54 (9.3)	0.70 (0.42 - 1.16)	
Site 10	ICDSC in ICU; CAM on ward	1383	193 (14.0)	699	95 (13.6)	684	98 (14.3)	0.95 (0.62 - 1.45)	
Site 11	ICDSC	958	87 (9.1)	472	45 (9.5)	486	42 (8.6)	1.11 (0.72 - 1.73)	
Site 12	CAM	1021	150 (14.7)	480	57 (11.9)	541	93 (17.2)	0.65 (0.45 - 0.93)	
Site 13	CAM	306	13 (4.3)	149	3 (2.0)	157	10 (6.4)	0.31 (0.07 - 1.45)	
Site 14	ICDSC	2345	223 (9.5)	1191	117 (9.8)	1154	106 (9.2)	1.11 (0.75 - 1.62)	

© 2025 Spence J et al. *JAMA Surgery*.

Site 15	ICDSC	876	56 (6.4)	405	28 (6.9)	471	28 (5.9)	0.99 (0.32 - 3.11)	
Site 16	CAM	240	27 (11.3)	122	15 (12.3)	118	12 (10.2)	1.24 (0.41 - 3.72)	
Site 17	ICDSC	1716	133 (7.8)	856	64 (7.5)	860	69 (8.0)	0.93 (0.59 - 1.47)	
Site 18	ICDSC	447	44 (9.8)	217	19 (8.8)	230	25 (10.9)	0.79 (0.42 - 1.47)	
Site 19	CAM	1255	457 (36.4)	602	214 (35.6)	653	243 (37.2)	0.93 (0.71 - 1.22)	
Site 20	CAM	986	161 (16.3)	486	80 (16.5)	500	81 (16.2)	1.00 (0.64 - 1.57)	

© 2025 Spence J et al. *JAMA Surgery*.

eFigure 3: Number of assessments documenting the presence of delirium per 12 hours after cardiac surgery by intervention allocation

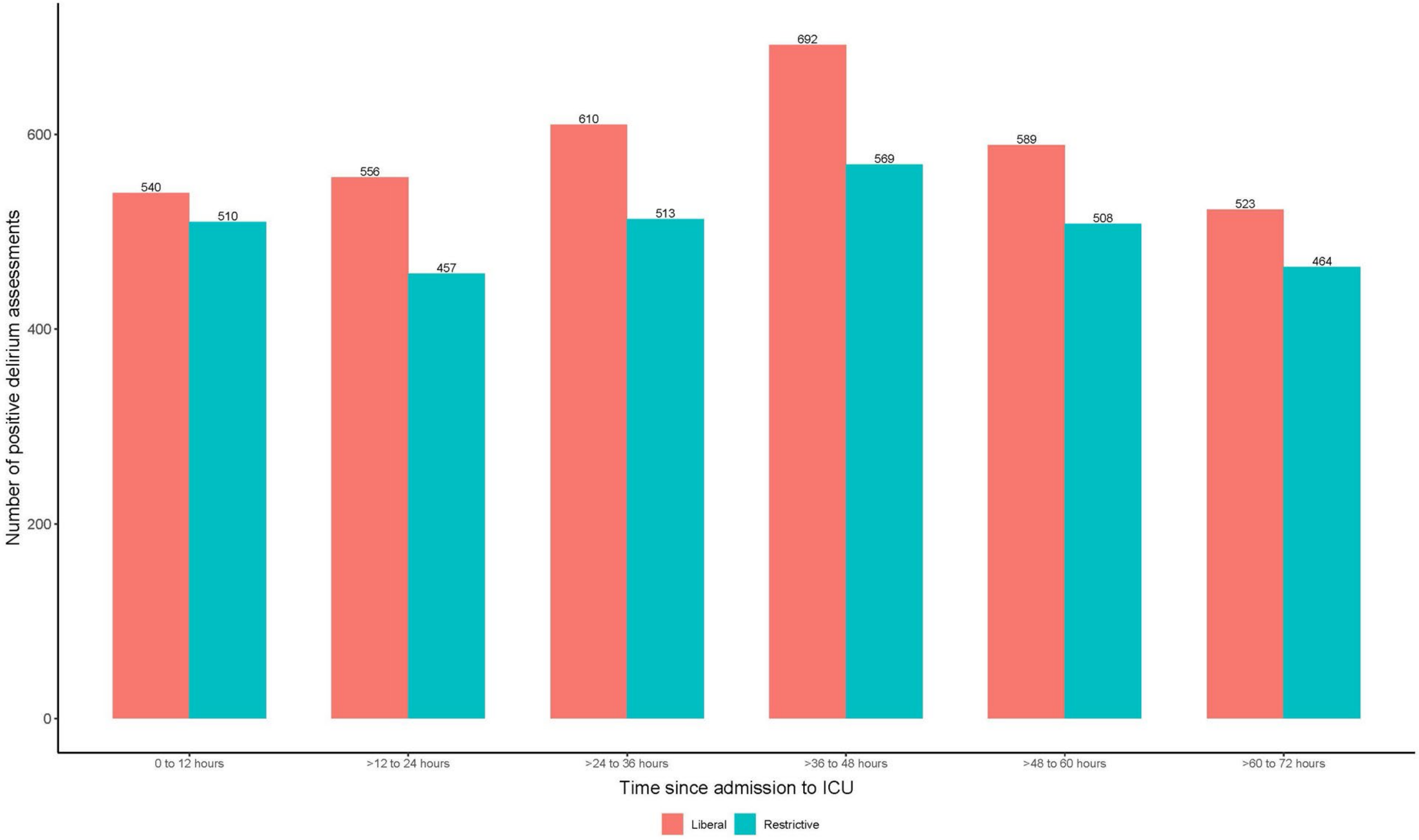

© 2025 Spence J et al. *JAMA Surgery*.

eTable 8: Sensitivity analysis excluding patients who were not assessed for delirium

Outcomes	Restricted benzodiazepine policy (N=9380)	Liberal benzodiazepine policy (N=9521)	Summary estimate (95% CI)	*P* value
Delirium up to 72h after cardiac surgery – no. (%)*	1373 (14.6)	1485 (15.6)	**aOR 0.92** (0.85 - 1.01)	0.08
Intensive care length of stay (days) - LSM (95% CI)*	3.3 (2.6, 4.2)	3.3 (2.6, 4.2)	**Mean difference 0.03** (-0.13 - 0.25)	0.74
Hospital length of stay (days) - LSM (95% CI)*	12.3 (11.2, 13.4)	12.4 (11.3, 13.6)	**Mean difference -0.10** (-0.61 – 0.18)	0.60
In-hospital mortality – no. (%)*	175 (1.9)	159 (1.7)	**aOR 1.15** (0.92 - 1.43)	0.22

Abbreviations: CI: confidence interval; aOR: adjusted odds ratio; LSM: least square means

*Analyses adjusted for age (years), sex, urgency of surgery (emergency vs elective), history of heavy alcohol consumption, and history of home benzodiazepine use

© 2025 Spence J et al. *JAMA Surgery*.
