# Supplementary material for: Benzodiazepine-Free Cardiac Anesthesia for Reduction of Postoperative Delirium: A Cluster Randomized Crossover Trial
Source: JAMA Surg. 2025 Jan 29;160(3):286–94. doi: 10.1001/jamasurg.2024.6602 (PMC11780505; doi:10.1001/jamasurg.2024.6602)
Supplement: Supplement 3. — B-Free Investigators and the Canadian Perioperative Anesthesia Clinical Trials Group [file jamasurg-e246602-s003.pdf]

Supplemental Online Content: Nonauthor Collaborators

*Indicates required information. Only first name, last name, and suffix will appear in PubMed.

*Group Name(s): B-Free Investigators and the Canadian Perioperative Anesthesia Clinical Trials group							
***First Name and Middle Initial(s)**	***Last Name**	***Suffix (eg, Jr, III)**	Academic Degrees	Institution	Location (city, state/province, country)	Role or Contribution, eg, chair, principal investigator	Group (if more than 1 Group listed in the byline) and/or Subgroup (eg, Steering Committee)
George	Wyse						
John	Eikelboom						
Tara	Robinson						
Gladys	Marfo						
Kumar	Balasubramanian						
Peter	Koh						
Courtney	Mullen			Hamilton General Hospital - Hamilton Health Sciences	Hamilton, Ontario, Canada		
Leah	Hayward			Hamilton General Hospital - Hamilton Health Sciences	Hamilton, Ontario, Canada		
Matthew Ryan	McFarling			Hamilton General Hospital - Hamilton Health Sciences	Hamilton, Ontario, Canada		
Kim	Botsford			Hamilton General Hospital - Hamilton Health Sciences	Hamilton, Ontario, Canada		
Summer	Syed			Hamilton General Hospital - Hamilton Health Sciences	Hamilton, Ontario, Canada		
Maia	Shen			Hamilton General Hospital - Hamilton Health Sciences	Hamilton, Ontario, Canada		
Renée	Fournier			Hamilton General Hospital - Hamilton Health Sciences	Hamilton, Ontario, Canada		
Morvarid	Kavosh			St. Boniface General Hospital	Winnipeg, Manitoba, Canada		
Kate	MacKenzie			St. Boniface General Hospital	Winnipeg, Manitoba, Canada		
Marita	Monterola			St. Boniface General Hospital	Winnipeg, Manitoba, Canada		
Linda	Girling			St. Boniface General Hospital	Winnipeg, Manitoba, Canada		

Supplemental Online Content: Nonauthor Collaborators

*Indicates required information. Only first name, last name, and suffix will appear in PubMed.

***First Name and Middle Initial(s)**	***Last Name**	***Suffix (eg, Jr, III)**	Academic Degrees	Institution	Location (city, state/province, country)	Role or Contribution, eg, chair, principal investigator	Group (if more than 1 Group listed in the byline) and/or Subgroup (eg, Steering Committee)
Mathilde	St-Pierre			Centre Hospitalier Universitaire de Sh	Sherbrooke, Québec, Canada		
Michel-Antoine	Perreault			Centre Hospitalier Universitaire de Sh	Sherbrooke, Québec, Canada		
Étienne	de Medicis			Centre Hospitalier Universitaire de Sh	Sherbrooke, Québec, Canada		
Jonathan	Gaulin			Centre Hospitalier Universitaire de Sh	Sherbrooke, Québec, Canada		
Nicola	Edward			St. Paul's Hospital	Vancouver, British Columbia, Canada		
Ron	Ree			St. Paul's Hospital	Vancouver, British Columbia, Canada		
Iris	Yao			St. Paul's Hospital	Vancouver, British Columbia, Canada		
Clement	Chui			St. Paul's Hospital	Vancouver, British Columbia, Canada		
Michelle	Biferie			St. Paul's Hospital	Vancouver, British Columbia, Canada		
Connie	Game			St. Paul's Hospital	Vancouver, British Columbia, Canada		
Juliet Ann	Atherstone			Vancouver General Hospital	Vancouver, British Columbia, Canada		
Darren	Mullane			Vancouver General Hospital	Vancouver, British Columbia, Canada		
Juliet	Atherstone			Vancouver General Hospital	Vancouver, British Columbia, Canada		
Michelle	Mozel			Royal Columbian Hospital	New Westminster, British Columbia, Canada		

Supplemental Online Content: Nonauthor Collaborators

*Indicates required information. Only first name, last name, and suffix will appear in PubMed.

***First Name and Middle Initial(s)**	***Last Name**	***Suffix (eg, Jr, III)**	Academic Degrees	Institution	Location (city, state/province, country)	Role or Contribution, eg, chair, principal investigator	Group (if more than 1 Group listed in the byline) and/or Subgroup (eg, Steering Committee)
Mikaela	Barton			Royal Columbian Hospital	New Westminster, British Columbia, Canada		
Ashley	Scott			Royal Columbian Hospital	New Westminster, British Columbia, Canada		
Aiman	Hasnat			Royal Columbian Hospital	New Westminster, British Columbia, Canada		
Ramiro	Arellano			Kingston General Hospital - Kingston Health Sciences Centre	Kingston, Ontario, Canada		
Robert	Tanzola			Kingston General Hospital - Kingston Health Sciences Centre	Kingston, Ontario, Canada		
Debbie	DuMerton			Kingston General Hospital - Kingston Health Sciences Centre	Kingston, Ontario, Canada		
Michael	Cummings			Kingston General Hospital - Kingston Health Sciences Centre	Kingston, Ontario, Canada		
Ahmad	Alli			St. Michael's Hospital	Toronto, Ontario, Canada		
Samson	Moses			St. Michael's Hospital	Toronto, Ontario, Canada		
Niloufar	Siadati-Fini			St. Michael's Hospital	Toronto, Ontario, Canada		
Kyle	Chin			St. Michael's Hospital	Toronto, Ontario, Canada		
Greg	Hare			St. Michael's Hospital	Toronto, Ontario, Canada		

Supplemental Online Content: Nonauthor Collaborators

*Indicates required information. Only first name, last name, and suffix will appear in PubMed.

***First Name and Middle Initial(s)**	***Last Name**	***Suffix (eg, Jr, III)**	Academic Degrees	Institution	Location (city, state/province, country)	Role or Contribution, eg, chair, principal investigator	Group (if more than 1 Group listed in the byline) and/or Subgroup (eg, Steering Committee)
Maliha	Muneer			Mazankowski Heart Institute	Edmonton, Alberta, Canada		
Izabela M	Panek			Queen Elizabeth II Health Sciences Centre	Halifax, Nova Scotia, Canada		
Kaela H	Fraser			Queen Elizabeth II Health Sciences Centre	Halifax, Nova Scotia, Canada		
Flynn A	Bonazza			Queen Elizabeth II Health Sciences Centre	Halifax, Nova Scotia, Canada		
Sharon E	Amey			Queen Elizabeth II Health Sciences Centre	Halifax, Nova Scotia, Canada		
Shelley	Roulston			University of Saskatchewan	Saskatoon, Saskatchewan, Canada		
Sophie	Robichaud			Montreal Heart Institute	Montreal, Québec, Canada		
Marco	Julien			Montreal Heart Institute	Montreal, Québec, Canada		
Antoine	Rochon			Montreal Heart Institute	Montreal, Québec, Canada		
Marie-Eve	Chamberland			Montreal Heart Institute	Montreal, Québec, Canada		
Meggie	Raymond			Montreal Heart Institute	Montreal, Québec, Canada		
Jennifer	Cogan			Montreal Heart Institute	Montreal, Québec, Canada		
Georges	Desjardins			Montreal Heart Institute	Montreal, Québec, Canada		
Jean-Sébastien	Lebon			Montreal Heart Institute	Montreal, Québec, Canada		
Christian	Ayoub			Montreal Heart Institute	Montreal, Québec, Canada		

*Indicates required information. Only first name, last name, and suffix will appear in PubMed.

***First Name and Middle Initial(s)**	***Last Name**	***Suffix (eg, Jr, III)**	Academic Degrees	Institution	Location (city, state/province, country)	Role or Contribution, eg, chair, principal investigator	Group (if more than 1 Group listed in the byline) and/or Subgroup (eg, Steering Committee)
Pierre	Couture			Montreal Heart Institute	Montreal, Québec, Canada		
Athanase	Courbe			Montreal Heart Institute	Montreal, Québec, Canada		
Maria Rosal	Martins			Montreal Heart Institute	Montreal, Québec, Canada		
André Y	Denault			Montreal Heart Institute	Montreal, Québec, Canada		
An Ni	Wu			Montreal Heart Institute	Montreal, Québec, Canada		
Kristofer	Beggs			Montreal Heart Institute	Montreal, Québec, Canada		
Stéphanie	Jarry			Montreal Heart Institute	Montreal, Québec, Canada		
Sarah	Bendaoud			Montreal Heart Institute	Montreal, Québec, Canada		
Ester	Cisneros-Aguilera			Montreal Heart Institute	Montreal, Québec, Canada		
Lana	Agoian			Montreal Heart Institute	Montreal, Québec, Canada		
Melissa	Laurendeau			Montreal Heart Institute	Montreal, Québec, Canada		
Elaine	Duval			Montreal Heart Institute	Montreal, Québec, Canada		
Anya	Chabane			Montreal Heart Institute	Montreal, Québec, Canada		
Martine	Lacroix			Montreal Heart Institute	Montreal, Québec, Canada		
Liam J	Kennedy			University Hospital - London Health Sciences Centre	London, Ontario, Canada		

Supplemental Online Content: Nonauthor Collaborators

*Indicates required information. Only first name, last name, and suffix will appear in PubMed.

***First Name and Middle Initial(s)**	***Last Name**	***Suffix (eg, Jr, III)**	Academic Degrees	Institution	Location (city, state/province, country)	Role or Contribution, eg, chair, principal investigator	Group (if more than 1 Group listed in the byline) and/or Subgroup (eg, Steering Committee)
Robert Christopher	Mayer			University Hospital - London Health Sciences Centre	London, Ontario, Canada		
Michelle	Clunie			Royal University Hospital	Saskatoon, Saskatchewan, Canada		
Azeez	Akinlade			Royal University Hospital	Saskatoon, Saskatchewan, Canada		
Hugo	Tremblay			Institut universitaire de cardiologie et de pneumologie de Québec - Université Laval	Québec City, Québec, Canada		
Nathalie	Gagné			Institut universitaire de cardiologie et de pneumologie de Québec - Université Laval	Québec City, Québec, Canada		
Annie	Bergeron			Institut universitaire de cardiologie et de pneumologie de Québec - Université Laval	Québec City, Québec, Canada		
François	Laforge			Institut universitaire de cardiologie et de pneumologie de Québec - Université Laval	Québec City, Québec, Canada		
Audrey	Grenier			Institut universitaire de cardiologie et de pneumologie de Québec - Université Laval	Québec City, Québec, Canada		
Valérie	Morin			Institut universitaire de cardiologie et de pneumologie de Québec - Université Laval	Québec City, Québec, Canada		
Sandrine	Bellavance			Institut universitaire de cardiologie et de pneumologie de Québec - Université Laval	Québec City, Québec, Canada		
Valérie	Lafrenière-Bessi			Institut universitaire de cardiologie et de pneumologie de Québec - Université Laval	Québec City, Québec, Canada		

Supplemental Online Content: Nonauthor Collaborators

*Indicates required information. Only first name, last name, and suffix will appear in PubMed.

***First Name and Middle Initial(s)**	***Last Name**	***Suffix (eg, Jr, III)**	Academic Degrees	Institution	Location (city, state/province, country)	Role or Contribution, eg, chair, principal investigator	Group (if more than 1 Group listed in the byline) and/or Subgroup (eg, Steering Committee)
Béatrice	Martin			Institut universitaire de cardiologie et de pneumologie de Québec - Université Laval	Québec City, Québec, Canada		
Joanie	Lachance			Institut universitaire de cardiologie et de pneumologie de Québec - Université Laval	Québec City, Québec, Canada		
Olivier	Audet			Institut universitaire de cardiologie et de pneumologie de Québec - Université Laval	Québec City, Québec, Canada		
Léa	Vachon-Zicat			Institut universitaire de cardiologie et de pneumologie de Québec - Université Laval	Québec City, Québec, Canada		
Yasmine	Babaki			Institut universitaire de cardiologie et de pneumologie de Québec - Université Laval	Québec City, Québec, Canada		
Élizabeth	St-Onge			Institut universitaire de cardiologie et de pneumologie de Québec - Université Laval	Québec City, Québec, Canada		
Nelson	Lavoie			Institut universitaire de cardiologie et de pneumologie de Québec - Université Laval	Québec City, Québec, Canada		
Mathilde	Bisson			Institut universitaire de cardiologie et de pneumologie de Québec - Université Laval	Québec City, Québec, Canada		
Marie-Ève	Charest			Institut universitaire de cardiologie et de pneumologie de Québec - Université Laval	Québec City, Québec, Canada		
Angela	Jerath			Sunnybrook Health Sciences	Toronto, Ontario, Canada		
Lilia	Kaustov			Sunnybrook Health Sciences	Toronto, Ontario, Canada		

Supplemental Online Content: Nonauthor Collaborators

*Indicates required information. Only first name, last name, and suffix will appear in PubMed.

***First Name and Middle Initial(s)**	***Last Name**	***Suffix (eg, Jr, III)**	Academic Degrees	Institution	Location (city, state/province, country)	Role or Contribution, eg, chair, principal investigator	Group (if more than 1 Group listed in the byline) and/or Subgroup (eg, Steering Committee)
Andrew	Fleet			Sunnybrook Health Sciences	Toronto, Ontario, Canada		
Sophia	Wong			Sunnybrook Health Sciences	Toronto, Ontario, Canada		
Elizabeth	Lappin			Sunnybrook Health Sciences	Toronto, Ontario, Canada		
Saba	Shaheen			Sunnybrook Health Sciences	Toronto, Ontario, Canada		
Mohammad	Helwani			Washington University School of Medicine	St. Louis, Missouri, United States		
Thaddeus	Budelier			Washington University School of Medicine	St. Louis, Missouri, United States		
Arianna	Montes de Oca			Washington University School of Medicine	St. Louis, Missouri, United States		
Alex	Kronzer			Washington University School of Medicine	St. Louis, Missouri, United States		
Meghann M	Fitzgerald			Weill Cornell Medicine	New York, New York, United States		
Natalia I	Girardi			Weill Cornell Medicine	New York, New York, United States		
Leonard N	Girardi			Weill Cornell Medicine	New York, New York, United States		
Michele L	Steinkamp			Weill Cornell Medicine	New York, New York, United States		
Lisbeth A	Evered			Weill Cornell Medicine	New York, New York, United States		
Hannah R	Leibowitz			Weill Cornell Medicine	New York, New York, United States		
Dylan R	Bitensky			Weill Cornell Medicine	New York, New York, United States		

Supplemental Online Content: Nonauthor Collaborators

*Indicates required information. Only first name, last name, and suffix will appear in PubMed.

***First Name and Middle Initial(s)**	***Last Name**	***Suffix (eg, Jr, III)**	Academic Degrees	Institution	Location (city, state/province, country)	Role or Contribution, eg, chair, principal investigator	Group (if more than 1 Group listed in the byline) and/or Subgroup (eg, Steering Committee)
Lisbeth	Evered			Weill Cornell Medicine	New York, New York, United States		
Alexis	Turgeon						
André	Denault						
Daniel	McIssac						
Kathryn	Sparrow						
Manoj	Lalu						
Stuart	McCluskey						
Richard	Hall						
William Scott	Beattie						
