# Supplementary material for: Benzodiazepine-Free Cardiac Anesthesia for Reduction of Postoperative Delirium: A Cluster Randomized Crossover Trial
Source: JAMA Surg. 2025 Jan 29;160(3):286–94. doi: 10.1001/jamasurg.2024.6602 (PMC11780505; doi:10.1001/jamasurg.2024.6602)
Supplement: Supplement 4. — Data sharing statement [file jamasurg-e246602-s004.pdf]

Data Sharing Statement

Spence. Benzodiazepine-Free Cardiac Anesthesia for Reduction of Postoperative Delirium. *JAMA Surg*. Published January 29, 2025. doi:10.1001/jamasurg.2024.6602

Data
Additional Information: Trial Registration: Clinicaltrials.gov registration number: NCT03928236; https://clinicaltrials.gov/study/NCT03928236
Data available: No

Additional Information
Explanation for why data not available: The Population Health Research Institute (PHRI) is the sponsor of this trial. The PHRI believes the dissemination of clinical research results is vital and sharing of data is important. PHRI prioritizes access to data analyses to researchers who have worked on the trial for a significant duration, have played substantial roles, and have participated in raising the funds to conduct the trial. PHRI balances the length of the research study and the intellectual and financial investments that made it possible with the need to allow wider access to the data collected. Data will be disclosed only upon request and approval of the proposed use of the data by a review committee. Data requests from other non-B-Free investigators will not be considered until 5 years after the close of the trial.
